# Supplementary material for: Linkage of maternity hospital episode statistics birth records to birth registration and notification records for births in England 2005–2006: quality assurance of linkage
Source: BMJ Open. 2020 Oct 26;10(10):e037885. doi: 10.1136/bmjopen-2020-037885 (PMC7592278; doi:10.1136/bmjopen-2020-037885)
Supplement: Supplementary data [file bmjopen-2020-037885supp001.pdf]

Supplementary information

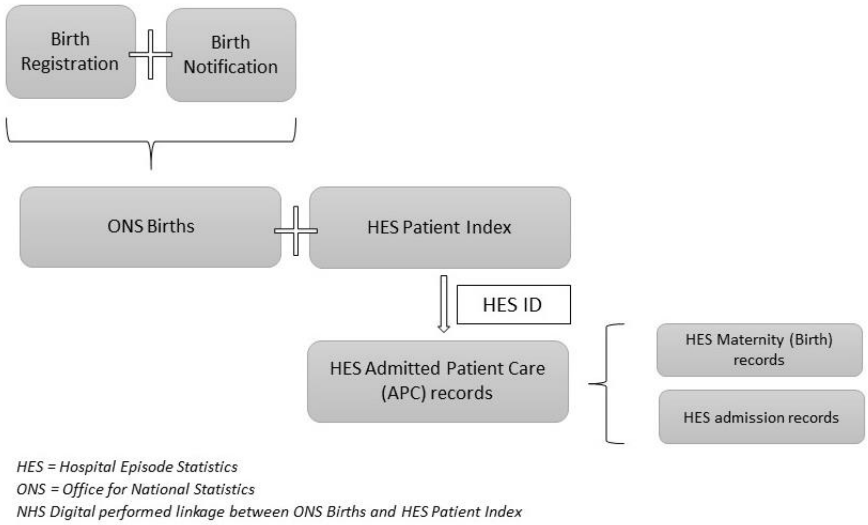

Figure S1. Overview of data sources

List of variables within the 'baby tail':

1. Anaesthetic given during labour or delivery (delpren)
2. Anaesthetic given post-labour or delivery (delposn)
3. Antenatal days of stay (antedur) (derived from other HES fields)
4. Baby's age in days (neodur) (derived from other HES fields)
5. Birth order (birorder)
6. Birth weight (birweit)
7. Delivery place change reason (delchang)
8. Delivery method (delmeth)
9. Delivery place (actual) (delplace)
10. Delivery place (intended) (delinten)
11. First antenatal assessment date (anasdate)
12. Gestation in weeks at first antenatal assessment (anagest)
13. Length of gestation (gestat)
14. Birth status (birstat)
15. Labour/delivery onset method (delonset)
16. Neonatel level of care (neocare)
17. Number of babies (numbaby)
18. Resuscitation method (biresus)
19. Status of person conducting delivery (delstat)

Coathup V, *et al. BMJ Open* 2020; 10:e037885. doi: 10.1136/bmjopen-2020-037885

Definitions of Episode, Spell and Continuous Inpatient Stay (CIP)

HES inpatient admissions are structured as 'episodes' of care, with an episode defined as a period of care under one consultant or midwife. Multiple episodes are referred to as a 'spell' and represent an uninterrupted period of care within one hospital. A new spell is generated when the patient is transferred to another hospital to continue care. A continuous inpatient stay (CIP) may consist of one episode or multiple episodes and spells, and ends when the patient is discharged from an NHS hospital. Hospital episodes are primarily collected for financial reimbursement, and therefore, the datasets are divided into financial years, beginning 1st April and ending 31st March. Episodes are labelled as 'finished' once the patient is discharged from hospital. However, if an episode begins in one financial year and ends during the next, two episodes will be generated – one in the financial year the episode begins and one in the financial year that the episode ends – therefore, the first episode will remain as 'unfinished'.

- Unfinished episodes identified using episode type (EPITYPE = 1)
- Transfers identified using admission method, admission source and discharge destination (ADMIMETH = 81; ADMISORC = 49/50/51/52/53/87; or DISDEST = 49/50/51/52/53/84).

Coathup V, *et al. BMJ Open* 2020; 10:e037885. doi: 10.1136/bmjopen-2020-037885

Table S1. NHS Digital algorithm used to link ONS Births with HES birth records

Match rank	Variables				Total		2005		2006	
	NHS number	Date of birth	Sex	Postcode	% Linked	% Broken	% Linked	% Broken	% Linked	% Broken
1	E	E	E	E	*80.43*	*28.23*	*75.68*	*24.61*	*85.01*	*34.45*
2	E	E	E	-	*19.20*	*9.41*	*24.02*	*8.76*	*14.52*	*10.42*
3	E	P°	E	E	*0.02*	*1.31*	*0.01*	*1.35*	*0.02*	*1.59*
4	E	P°	E	-	*0.01*	*1.03*	*0.01*	*1.12*	*0.01*	*0.71*
5	E	-	-	E	*0.02*	*2.61*	*0.02*	*1.57*	*0.03*	*4.59*
6	M	E*	E	E^	*0.31*	*53.50*	*0.22*	*58.76*	*0.40*	*44.52*
7	M	E*	E	E	*0.01*	*1.10*	*0.01*	*1.35*	*0.01*	*0.68*
8	E	-	-	-	*0.02*	*2.82*	*0.03*	*2.47*	*0.01*	*3.13*
Number of NHS births					1,073,114	1,456	525,826	890	100	566

E=Exact match, P=Partial match, M=No match, due to missing data
°Two components of data match or DOB matches, when two components are swapped
**Date of birth not 1st January*
^Postcode not on 'ignore' list

Coathup V, *et al. BMJ Open* 2020; 10:e037885. doi: 10.1136/bmjopen-2020-037885

Table S2. Sources of variables for quality assurance

Variable	Source	Rule
Baby's date of birth	BR & BN	Use BR unless BR = missing, then use BN
Mother's date of birth	BR & BN	Use BR unless BR = missing, then use BN
Birth weight	BR & BN	Use BR unless BR = missing, then use BN
Gestational age	BR & BN	use BN as BR reported for still births only
Sex	BR & BN	Use BR unless BR = missing, then use BN
Hospital of birth	BR & BN	Use BR unless BR = missing, then use BN

BR = Birth Registration
BN = Birth Notification (NN4B)

Table S3. Data preparation rules for quality assurance variables

Variable	Variable name: HES	Variable name: BR/BN	Rule	Additional checks
Baby's date of birth	DOBBABY	DOB/DOBZ	Set '01Jan1801' to missing. Make sure all dates are in format 'DDMMMYYYY' e.g. '01Jan2005'.	Check dates are not <01/01/05 or > 31/12/06
Mother's date of birth	MOTDOB	DOBM/DOBMZ	Set '01Jan1801' to missing. Make sure all dates are in format 'DDMMMYYYY' e.g. '01Jan2005'	
Postcode	HOMEADD	PCDRBZ (BN)	Transform var into format of 'XXX XXX', by removing any additional punctuation and transforming any lower case letters to upper case	
Birth weight	BIRWEIT	BTHWGTZ (BN)	Set '99', '9999', <400 and ≥7000 to missing	Check all hospitals report birth weight in g. If it looks like kg, then convert to equivalent in g.
Gestational age	GESTAT	GESTZ (BN)	Set '99' to missing	Check all hospital report gestational age in weeks, if not, convert from days or months to weeks
Sex	SEXBABY	SEXZ (BN)	Transform M to 1 and F to 2 if string variable. Set '0', '3' or '9' to missing.	
Hospital of birth	PROCODE	Location Code (derived from CESTRESS/ORGCODEZ)	Remove punctuation. Transform any lower case letters to upper case	

HES = Hospital Episode Statistics
BR = Birth Registration, BN= Birth Notification (NN4B)

Coathup V, *et al. BMJ Open* 2020; 10:e037885. doi: 10.1136/bmjopen-2020-037885

Table S4. Percentages of data missing from each dataset for key variables used to evaluate linkage of data about live, singleton births in NHS hospitals to women living in England

	ONS Births			HES Births		
**Variable**	**Total**	**2005**	**2006**	**Total**	**2005**	**2006**
NHS number	0.1	0.1	0.1	6.9	6.5	7.4
Baby's date of birth	0.0	0.0	0.0	0.0	0.0	0.0
Mother's date of birth	0.0	0.0	0.0	42.7	42.9	42.4
Mother's postcode	0.0	0.0	0.0	60.4	76.2	45.0
Hospital location code	0.0	0.0	0.0	0.0	0.0	0.0
Birthweight	0.1	0.1	0.1	44.4	43.8	45.1
Gestational age	0.8	0.8	0.8	49.1	45.7	52.6
Sex	0.0	0.0	0.0	0.0	0.0	0.0

Coathup V, *et al. BMJ Open* 2020; 10:e037885. doi: 10.1136/bmjopen-2020-037885

Steps taken to deal with multiple HES birth records with the same HESID:

1. EPISTAT is 1 = unfinished episode: exclude all (for details of HES variables, see *NHS Digital Data Dictionary)*
2. HESID and EPIKEY exact match = duplicate record: keep any and exclude rest
3. HESID, DOB, Sex, hospital code, EPIORDER, ADMIDATE, DISDATE, EPISTART, EPIEND, DIAG_01 – DIAG_20, OPCS_01-OPCS_24, OPDATE_01-OPDATE_24 exact match and MDOB and POSTCODE either exact match or missing match = duplicate record: keep any and exclude rest
4. Two or more key variables (date of birth, Hospital code, mother's date of birth, postcode, birthweight) differ completely = unclear which record contains correct information: exclude all
5. Remainder are considered multiple episodes part of same spell = create new record using all available information from multiple episodes: keep one record and save other episodes in separate file
6. Dataset should now contain one HES birth record per baby
