# Supplementary material for: Linkage of maternity hospital episode statistics birth records to birth registration and notification records for births in England 2005–2006: quality assurance of linkage
Source: BMJ Open. 2020 Oct 26;10(10):e037885. doi: 10.1136/bmjopen-2020-037885 (PMC7592278; doi:10.1136/bmjopen-2020-037885)
Supplement: Author's manuscript [file bmjopen-2020-037885.draft_revisions.pdf]

BMJ Open is committed to open peer review. As part of this commitment we make the peer review history of every article we publish publicly available.

When an article is published we post the peer reviewers' comments and the authors' responses online. We also post the versions of the paper that were used during peer review. These are the versions that the peer review comments apply to.

The versions of the paper that follow are the versions that were submitted during the peer review process. They are not the versions of record or the final published versions. They should not be cited or distributed as the published version of this manuscript.

BMJ Open is an open access journal and the full, final, typeset and author-corrected version of record of the manuscript is available on our site with no access controls, subscription charges or pay-per-view fees (http://bmjopen.bmj.com).

If you have any questions on BMJ Open's open peer review process please email info.bmjopen@bmj.com

Linkage of Maternity Hospital Episode Statistics data to birth registration and notification records for births in England 2005-2006: Quality assurance of linkage with singleton Hospital Episode Statistics birth records

Journal:	*BMJ Open*
Manuscript ID	bmjopen-2020-037885
Article Type:	Original research
Date Submitted by the Author:	21-Feb-2020
Complete List of Authors:	Coathup, Victoria; University of Oxford, Nuffield Department of Population Health<br>Macfarlane, Alison; City University, Dept of Midwifery<br>Quigley, Maria; Oxford University, National Perinatal Epidemiology Unit
Keywords:	PUBLIC HEALTH, Protocols & guidelines < HEALTH SERVICES ADMINISTRATION & MANAGEMENT, Clinical governance < HEALTH SERVICES ADMINISTRATION & MANAGEMENT

SCHOLARONE™
Manuscripts

I, the Submitting Author has the right to grant and does grant on behalf of all authors of the Work (as defined in the below author licence), an exclusive licence and/or a non-exclusive licence for contributions from authors who are: i) UK Crown employees; ii) where BMJ has agreed a CC-BY licence shall apply, and/or iii) in accordance with the terms applicable for US Federal Government officers or employees acting as part of their official duties; on a worldwide, perpetual, irrevocable, royalty-free basis to BMJ Publishing Group Ltd ("BMJ") its licensees and where the relevant Journal is co-owned by BMJ to the co-owners of the Journal, to publish the Work in this journal and any other BMJ products and to exploit all rights, as set out in our licence.

The Submitting Author accepts and understands that any supply made under these terms is made by BMJ to the Submitting Author unless you are acting as an employee on behalf of your employer or a postgraduate student of an affiliated institution which is paying any applicable article publishing charge ("APC") for Open Access articles. Where the Submitting Author wishes to make the Work available on an Open Access basis (and intends to pay the relevant APC), the terms of reuse of such Open Access shall be governed by a Creative Commons licence – details of these licences and which Creative Commons licence will apply to this Work are set out in our licence referred to above.

Other than as permitted in any relevant BMJ Author's Self Archiving Policies, I confirm this Work has not been accepted for publication elsewhere, is not being considered for publication elsewhere and does not duplicate material already published. I confirm all authors consent to publication of this Work and authorise the granting of this licence.

For peer review only

Linkage of Maternity Hospital Episode Statistics data to birth registration and notification records for births in England 2005-2006: Quality assurance of linkage with singleton Hospital Episode Statistics birth records

Dr Victoria Coathup[1], Professor Alison Macfarlane[2], Professor Maria Quigley[1]

[1]National Perinatal Epidemiology Unit (NPEU), University of Oxford, Oxford, UK

[2]Department of Midwifery, School of Health Sciences, City, University of London, UK

Corresponding author

Victoria Coathup

National Perinatal Epidemiology Unit (NPEU)

Nuffield Department of Population Health

Old Road Campus

Oxford, OX3 7LF

victoria.coathup@npeu.ox.ac.uk

Abstract

Objectives: The objectives of this study were to describe the methods used to assess the quality of linkage between records of babies' birth registration records and hospital birth records, and to evaluate the potential bias that may be introduced because of these methods.

Design/Setting: Linked data from the civil registration and the notification of births from the Office for National Statistics (ONS) were further linked by NHS Digital to birth records from the Hospital Episode Statistics (HES) for babies born in England. We developed a deterministic, six stage algorithm to assess the quality of this linkage.

Participants: All live, singleton births, occurring in NHS hospitals in England between 1st January 2005 and 31st December 2006 (n=1,170,790).

Primary outcome measure: The primary outcome was the number of successful links between ONS birth records and HES birth records. Rates of successful linkage were calculated for the cohort and the characteristics associated with unsuccessful linkage were identified.

Results: Approximately 92% (n=1,074,572) of the birth registration records were successfully linked with a HES birth record. Data quality and completeness was somewhat poorer in HES birth records compared to birth registration and birth notification records. The quality assurance algorithms identified 1,456 incorrect linkages (<1%). Compared to the linked dataset, birth records were more likely to be unlinked if babies were of white ethnic origin; born to unmarried mothers; born in East England, London, North West England or the West Midlands; or born in March.

Conclusions: It is possible to link administrative datasets to create large cohorts allowing researchers to explore important questions about exposures and childhood outcomes. Missing data and coding errors and inconsistencies mean it is important that the quality of linkage is assessed prior to analysis, however.

Keywords

Births, hospital records, data-linkage, Hospital Episode Statistics

Strengths and limitations of this study

1. This is the first study to evaluate the linkage of birth registration, birth notification and HES birth records in England.
2. We were able to build on existing work relating to quality assurance of birth registration, birth notification and HES delivery records.
3. Access to personal identifiers meant we were able to evaluate the quality of linkage, identify poor quality matches and assess the level of bias introduced by linking these datasets.
4. Accessing personal identifiers also led to delays because of the length of approval processes and the need to access these data in designated secure settings. However, the Office for National Statistics is moving towards a remote access system, so future research studies may not be affected in the same way.
5. We examined only singleton births in NHS hospitals so it remains unclear whether the linkage of multiple births is of comparable quality. However, previous work linking birth registration records with mothers' delivery records in multiples suggests that the linkage rate is similar to singletons

For peer review only

Introduction

The use of routinely collected datasets within research has increased rapidly over the past decade as an alternative to conducting large, observational studies, which can be extremely costly and often suffer from poor recruitment and retention rates[1]. Whilst there are many advantages to using linked administrative data for medical research, they also present challenges. One is the quality of data recorded and, in consequence, the quality of data linkage, as this is highly reliant upon the availability and accuracy of personal identifiers[2] and other supporting information.

The Digital Economy Act (DEA) 2017[3] was introduced with the aim of facilitating data sharing for research purposes, but only if the data have been 'de-identified' and the research is deemed to be in the public interest[4]. Without access to personal identifiers, successful linkage between datasets becomes more challenging. Therefore, the 'Trusted Third Party' model, whereby the full datasets are transferred to an organisation, which will perform the linkage under contract with the data controller, is now the preferred method of linkage in most research projects. Whilst Trust Third Parties typically publish their linkage algorithms, they usually do not publish results of quality assurance of their methods. Therefore, it is essential that researchers assess the quality of linkage and validity of data prior to conducting statistical analyses.

The work described in this paper was conducted as part of the TIGAR study (Tracking the Impact of Gestational Age on Health, Educational and Economic outcomes: a Longitudinal Records Linkage Study), which is a population-based, record-linkage study of births and hospital admission data in England. The study aimed to estimate the association between gestational age at birth and rates of hospital admission throughout childhood. This used previously linked data recorded at civil registration of births and data recorded when the birth is notified to the NHS and the baby's NHS Number is allocated and linked them with birth records from the Hospital Episode Statistics (HES). These records were linked by NHS Digital as part of another, larger study by City, University of London[5].

Two HES records are generated when a baby is born in an NHS maternity unit in England: (1) the mother's delivery record; and (2) the baby's birth record. The team at City, University of London has already evaluated the quality of linkage between birth registration, birth notification and the mother's HES delivery records[6]. Therefore, this paper describes the quality assurance checks performed on birth registration and notification records linked to the baby's HES birth records. The main objectives of the current study were to assess the quality of linkage between baby's birth registration, birth notification and HES birth records, and to evaluate the potential bias introduced to the study cohort by the linkage. This has relevance for analyses of similar linked administrative datasets.

For peer review only

Methods

All live, singleton babies born in NHS hospitals between 1st January 2005 and 31st December 2006 to a mother living in England were eligible for inclusion in the study cohort. All analyses was conducted using STATA 14[7] within the Office for National Statistics (ONS)' Secure Research Service (SRS). An overview of the procedures involved in the quality assurance process for this study is presented in **Figure 1**.

Figure 1. Flow chart of study population

Data sources

Two datasets containing data about born in England from 1st January 2005 to 31st December 2006 were linked (**Figure S1**). They and the linkage file were saved as three separate STATA datasets. (A) ONS Births; (B) Hospital Episode Statistics (HES birth records); and (C) the Linkage file containing unique ONS birth identifiers (ONS_id) and corresponding unique HES identifiers (HESID). These files are described in the supplementary information (**Figure S1**).

A. ONS births

The master dataset comprises data from two sources: birth registration and birth notification (birth notification replaces NHS numbers for babies [NN4B]). These two datasets have been routinely linked by the ONS since 2007. The combined dataset is referred to as 'ONS births' throughout this paper. ONS births contain personal identifiers, socio-demographic characteristics and birth characteristics.[8]

B. Hospital Episode Statistics (HES Birth records)

HES is a large database containing records of all episodes of care and births in NHS hospitals in England since 1989. The records used here are from the HES Admitted Patient Care (APC) dataset. This is described in detail elsewhere[9] but brief definitions of episodes and spells are in the supplementary information. Everyone for whom records are stored in HES is assigned a HESID[10] generated using a combination of NHS Number, local patient identifier, postcode, sex and date of birth to enable data users to uniquely track patients throughout the NHS. Within this study, data were obtained from the HES APC dataset, which includes birth and delivery records. Descriptions of the variables available are in NHS Digital's HES Data Dictionary[11].

When a baby is born, the general inpatient episode becomes part of the Maternity HES dataset and 19 additional variables relating to the birth are appended. These additional data items form the 'baby tail'.

For peer review only

Maternity HES contains two types of maternity records:

I. HES delivery records, which include all data items relevant to the mother's delivery

II. HES birth records, which are the baby based records, contain all data items relating to the baby born

This study evaluates the linkage of the HES birth records only. Linkage between birth registration and HES delivery records had already been evaluated.[6]

C. Linkage file

The linkage file contained the unique identifiers from each dataset as linked by NHS Digital. The dataset contained unique identifiers from ONS births that had been successfully linked with the corresponding unique identifier from HES births and those which had not. It also contained unique HES birth identifiers that were not linked to a unique ONS birth identifier.

Linkage of the Baby cohort and Maternity HES

As described previously[12], ONS birth records were linked to the HES patient index by NHS Digital using a deterministic algorithm in order to assign the HESID and then records were linked with the corresponding HES birth record. Here, each linked record was assigned a match rank score, indicating the stage in the algorithm at which the records had been matched (1 being highest quality). Steps of the algorithm are summarised in **Table S1**.

Data preparation for quality assurance checks

Full details of available variables, data cleaning procedures and preparation steps for the included datasets have are described elsewhere.[5] Many key variables were available from both birth registration and birth notification. The preferred sources are summarised in the supplementary information (**Table S2)**. A number of additional steps were taken to ensure that key variables in HES births and ONS births were in consistent formats, ready for comparison during the quality assurance procedure (**Table S3**). Further checks were conducted on HES birth records and those with: (1) discharge date occurring before admission; (2) discharge occurring before baby's date of birth; or (3) admission dates occurring before 1st January 2005 or after 31st December 2006 were excluded.

Multiple birth episodes with the same HESID (i.e. duplicate HES records)

Birth episodes with the same HESID can occur due to a number of reasons: (1) unfinished episodes, such as a baby being born on the 30th March, but then discharged on the 1st April[13]; (2) administrative errors[6]; (3) a birth

For peer review only

spell containing multiple episodes, such as a baby being transferred to a different hospital or consultant; and (4) HESID is incorrectly assigned to more than one baby[14]. A number of steps were taken which have been summarised in the supplementary information. In addition to this, episode start dates that occurred more than two days after the previous episode ended and did not include a transfer code (see supplementary information) were considered a new admission and were saved separately.

Quality assurance of the baby cohort and Maternity HES linkage

After merging the three datasets, we identified cases where two different ONS birth records had been linked to the same HES birth record and key characteristics were compared to identify the correct link. This was done by comparing the following variables which were available in both data sources: baby's date of birth, gestational age, birth weight, sex, mother's date of birth and postcode. The record with the highest number of matching variables was identified as the correct link. When records matched on the same number of variables, the record that matched exactly on birthweight was identified as the correct match. If birthweight was missing or did not match with either record, then the record with the highest match rank score from the original linkage (Table S1) was identified as the correct link. The remaining records were then compared manually, but in cases where records had a high proportion of missing data or the same number of matching variables and the same match rank score, it was not possible to identify the correct link and therefore all were excluded.

The deterministic algorithm developed for evaluating the record linkage is summarised in **Table 1**. The quality assurance (QA) algorithm was adapted from a previous study which assessed the quality of linkage between ONS birth records and the mother's HES delivery records[6], and was based on location of birth, date of birth, sex, birthweight and gestational age. The location of birth was defined as the hospital trust running the hospital that the baby was born in. A hospital trust in the UK is an organisational unit within the NHS and usually refers to a group of hospitals that are in close proximity to each other. This was used instead of the hospital of birth to account for potential transfers between hospitals within the birth admission. The hospital trust variable was developed as part of a previous study[5].To account for differences in rounding between hospitals, a birthweight of + or – 100g was considered a match.

For peer review only

Table 1. Quality assurance algorithm to assess linkage of ONS birth records to HES birth records

Step	Variable												
	**Hospital Trust code**		**Date of birth**		**Sex**		**Birthweight**		**Gestational age**		**Mother's date of birth**		**Postcode**
1	E	*and*	E	*and*	E	*and*	E	*and*	E		-		-
2	E	*and*	E	*and*	E	*and*	(E	*or*	E)		-		-
3	E	*and*	E	*and*	E		-		-	*and*	E/M	*and*	E/M
4	-		E	*and*	E	*and*	E/M	*and*	E/M	*and*	E/M	*and*	E/M
5	E	*and*	P	*and*	E	*and*	(E	*or*	E	*or*	E	*or*	E)
6	E	*and*	P		-		E/M	*and*	E/M	*and*	E/M	*and*	E/M

E = Exact match; M = Missing; P = Partial match (differs by up to four days, two elements of data match or dates match if day and month swapped)

For peer review only

Final check after the quality assurance procedures were completed included: (1) Checking that the baby's date of birth in ONS births was within the admission and discharge dates from the HES birth record; (2) Baby's date of birth in ONS births did not occur before 1st January 2005 or after 31st December 2006; (3) hospital discharge date did not occur before the admission date; and (4) further checks to ensure that all stillbirths had been excluded (ICD10 diagnosis code = Z37.1).

Assessment of linkage bias

The distributions of key characteristics were compared between the linked and unlinked study samples and the eligible study sample. The chi-squared test was used to assess whether distributions differed significantly between the linked and unlinked study samples. Because the sample sizes were so large we also looked at differences in distributions of variables, for example, differences between proportions of more than 1%. In addition to this, we compared the distributions of key characteristics between the linked study sample and all live births in England in 2005 and 2006 to see what effect excluding stillbirths, multiple and non-NHS births had on the study cohort.

Patient and Public Involvement

The TIGAR study was supported by a patient, parent and public advisory group, which provided input to different aspects of the study. This group met at the start of the study and gave input into the study protocol and the lay summary of the project.

Ethics approval

Ethics approval for this study was granted by the Health Research Authority Research Ethics Committee (South West – Frenchay; REC reference 15/SW/0294) and the Confidential Advisory Group (CAG reference 15/CAG/0196).

Results

There were 1,257,884 ONS birth records. After excluding babies who were not eligible for the TIGAR study, 1,170,790 live, singleton babies born from 1st January 2005 to 31st December 2006 in NHS hospitals to women living in England remained in the study cohort. There were 1,243,373 HES birth records and the linkage file included 1,242,938 records (**Figure 1)**.

Linkage of baby cohort to Maternity HES

Of the 1,170,790 eligible ONS birth records, 1,074,572 or 92% were successfully linked with a HES birth record. Of the 96,218 unlinked records, 88,471 had a HESID but no corresponding HES birth record and 7,747 had no corresponding HESID in the linkage file. The majority of records linked had a match rank score of one, which meant they exactly matched on all four variables in the NHS Digital algorithm, with fewer than 1% of records linking in stages three to six of the algorithm (Table S1). A higher proportion of links had a match rank score of one in 2006 compared to 2005, however.

For peer review only - http://bmjopen.bmj.com/site/about/guidelines.xhtml

Data preparation for quality assurance checks

All key variables in ONS birth records had data missing for less than 1% of births. In contrast, data were missing from substantial numbers of HES birth records (**Table S4)**.

Multiple birth episodes with the same HESID

Of the 1,243,373 HES birth records, 73,307 (6%) had linked with more than one record with the same HESID. Of these, the most common reasons for this were multiple episodes within a hospital spell (62%), unfinished episodes (15%) and duplicate episodes due to administrative errors (11%) (**Figure 1**). There were 1,197,999 unique HES birth records remaining.

Quality assurance of the baby cohort and Maternity HES linkage

A total of 979 (0.1%) HES birth records had linked with more than one ONS birth record. The high proportion of missing data in HES birth records meant it was difficult in many cases to identify the correct match. Of these, 499 records were judged to be incorrectly linked and were broken. It was not possible to ascertain the status of 95 records, which had the same matching variables and match rank score. A lot of data items in these records were missing data and all were all excluded (**Table 2)**.

For peer review only

For peer review only - http://bmjopen.bmj.com/site/about/guidelines.xhtml

Table 2. Proportions of birth records at each quality assurance stage in 2005 and 2006 for live, singleton births born in NHS hospitals to mother's living in England

Quality assurance stage		Total	2005		2006	
		n	n	%	n	%
1	Number of Births*	1,170,790	575,568	*49.2*	595,222	*50.8*
2	Number of ONS Birth records linked to HESID	1,148,110	563,963	*49.1*	584,147	*50.9*
3	Number of ONS Birth records not linked to HESID	22,680	11,605	*51.2*	11,075	*48.8*
4	Number of ONS Birth records linked to HESID and HES birth record	1,074,571	530,586	*49.4*	543,985	*50.6*
5	Number of ONS Birth records linked to HESID but not linked to HES birth record	96,219	44,982	*46.7*	51,237	*53.3*
6	Number of ONS Birth records linked to one HES birth record before QA	1,074,571	530,586	*49.4*	543,985	*50.6*
7	Number of links broken between ONS Birth records and HES birth records after QA	1,456	890	*61.1*	566	*38.9*
8	Number of ONS Birth records linked to one HES birth record after QA	1,073,115	530,119	*49.4*	542,996	*50.6*
-	% of total ONS birth records left linked to one HES birth record after QA	91.7	-	*92.1*	-	*91.3*
9	Total number of ONS Birth records not linked to HES birth record after QA	97,675	45,810	*46.9*	51,865	*53.1*
-	% of total ONS birth records not linked to HES birth record after QA	8.3	-	*7.9*	-	*8.7*

**Birth registration linked to birth notification records of live, singleton births that occurred in NHS hospitals to women residing in England*

For peer review only

For peer review only - http://bmjopen.bmj.com/site/about/guidelines.xhtml

Key variables of the 1,074,572 (92%) ONS birth records, which had been successfully linked, were then compared using the algorithm in **Table 1**. The vast majority (99.5%) of correct links were found to be within the first three stages of the quality assurance algorithm (**Table 3**). The 645 records identified as correct links in stage four of the algorithm appeared to be correct matches; the hospital location code differed slightly, which may have resulted from either a transfer to a neighbouring hospital trust during the birth admission or from data entry errors. In stage five, 1,973 (0.2%) records were identified as correct links. Of these, 76% had a match rank score of one, suggesting the date of birth had been entered incorrectly in the HES birth record. The majority of these matches looked like data entry errors where the month and day had inadvertently been swapped. In stage six of the algorithm, a small number of records were identified as correct links. These records had a lot of data missing, including 64% of birthweights, 70% of gestational ages, 77% of postcodes; and 67% of mother's dates of birth. Of the 242 records with partially matching dates of births, 91% had a match rank score of one or two, suggesting good quality matches but with data entry errors in the HES birth records. Interestingly, almost 62% of records in this stage exactly matched on date of birth, but differed by sex. Most of these records also exactly matched on birthweight, gestational age, postcode and mother's date of birth, suggesting they are correct links, again with data entry errors in the HES birth record.

Table 3. Proportion of birth records identified as correct links at each stage of the quality assurance process

Step	Total		2005		2006	
	Number	%	Number	%	Number	%
**1**	385,570	*35.9*	207,106	*39.1*	178,464	*32.8*
**2**	301,066	*28.0*	134,684	*25.4*	166,382	*30.6*
**3**	383,189	*35.6*	186,203	*35.1*	196,986	*36.2*
**4**	645	*0.1*	426	*0.1*	219	*0.0*
**5**	1,973	*0.2*	983	*0.2*	990	*0.2*
**6**	671	*0.0*	379	*0.0*	292	*0.0*
**Poor quality links**	**860**	***0.1***	**548**	***0.1***	**312**	***0.1***
**All**	**1,074,571**	***100.0***	**530,586**	***100.0***	**543,985**	***100.0***

Overall, 860 records with incorrect links were identified and 64% of these were in births in 2005, suggesting an improvement in data and linkage quality in 2006. When exploring these broken links in relation to their match rank score, 69% had a score of six, meaning their NHS numbers were missing. The main reason for broken links was completely different dates of birth as opposed to exact or partial matching and data missing for large numbers of variables. Among these records, birthweight, gestational age, postcode and mother's date of birth was missing for approximately 92%, 91%, 85% and 91%, respectively. There were a small number of records that appeared to be incorrectly broken due to cleaning errors, for example postcodes which included an 'O' instead of a zero.

A number of interesting observations was made when reviewing the broken links: (1) Many that were broken differed by gestational age, but in most cases by just one week and the babies were full term for example 39 and

For peer review only - http://bmjopen.bmj.com/site/about/guidelines.xhtml

40 weeks; and (2) More than one third of broken links were for babies born in London. As the numbers of broken links were small, distributions of numbers of broken links cannot be presented, as they could be disclosive.

Assessment of linkage bias

Distributions of key variables for all live births, all eligible births, and all linked and all unlinked births are presented in **Table 4**. Compared to the linked dataset, the distribution of those in the unlinked dataset differed by baby's ethnicity, mother's age, parity, Index of Multiple Deprivation score (IMD) score, sex, registration status, region and month of birth, birth weight and gestational age ($p<0.05$). Although absolute differences were small (<1%) for most variables, there were some noticeable discrepancies, where distributions differed by more than 1%. Unlinked records were more likely occur if babies were of white ethnic origin; born outside marriage; born in East England, London, North West England or the West Midlands; or born in March. Compared to all eligible births in 2005 and 2006, the distributions of linked births were very similar and differed by less than 1% in all cases.

For peer review only

BMJ Open

Table 4. Comparison of all live, all eligible, all inked and all unlinked datasets

	All live births (n=1,250,490)		All eligible (n=1,170,790)		Linked dataset (n=1,073,114)		Unlinked (n=97,674)		
	n	%	n	%	n	%	n	%	p-value[a]
**Ethnicity**									
Bangladeshi	16,730	*1.3*	16,281	*1.4*	15,356	*1.4*	925	*1.0*	p<0.000
Indian	32,241	*2.6*	31,287	*2.7*	28,970	*2.7*	2,318	*2.4*	
Pakistani	48,570	*3.9*	47,352	*4.0*	43,702	*4.1*	3,648	*3.7*	
Black African	24,461	*2.0*	23,394	*2.0*	21,504	*2.0*	1888	*1.9*	
Black Caribbean	8,668	*0.7*	8,200	*0.7*	7,420	*0.7*	780	*0.8*	
White British	788,98	*63.0*	738,887	*63.1*	675,34	*62.9*	63,552	*65.0*	
White Other	91,311	*7.3*	86,918	*7.4*	80,611	*7.5*	6,306	*6.5*	
Other	102,04	*8.2*	97,362	*8.3*	89,043	*8.3*	8,318	*8.5*	
Missing	137,47	*10.9*	121,109	*10.3*	111,16	*10.3*	9,939	*10.1*	
**Mother's age**									
Under20	52,605	*4.2*	51,312	*4.4*	46,803	*4.4*	4,509	*4.6*	p<0.000
20-24	216,76	*17.3*	208,688	*17.8*	190,72	*17.7*	17,960	*18.3*	
25-29	306,60	*24.5*	290,132	*24.7*	266,00	*24.7*	2,130	*24.7*	
30-34	363,31	*29.0*	337,323	*28.8*	309,60	*28.8*	27,718	*28.3*	
35-39	244,95	*19.5*	223,256	*19.0*	204,99	*19.1*	18,263	*18.7*	
40+	66,250	*5.3*	60,079	*5.1*	54,987	*5.1*	5,04	*5.2*	
**Nulliparous**									
No	587,83	*47.0*	562,207	*48.0*	523,05	*48.7*	39,152	*40.0*	p<0.000
Yes	570,12	*45.5*	548,258	*46.8*	505,98	*47.1*	42,279	*43.2*	
Missing	92,527	*7.4*	60,325	*5.2*	44,082	*4.1*	16,243	*16.6*	
**IMD Score (quintiles)**									
Q1	333,80	*26.6*	318,830	*27.2*	292,31	*27.2*	26,513	*27.1*	p=0.001
Q2	263,21	*21.0*	247,779	*21.1*	227,08	*21.1*	20,700	*21.1*	
Q3	221,72	*17.7*	206,648	*17.6*	189,75	*17.6*	16,891	*17.2*	
Q4	200,89	*16.0*	186,165	*15.9*	170,31	*15.8*	15,851	*16.2*	
Q5	195,38	*15.6*	181,041	*15.4*	165,76	*15.4*	15,277	*15.6*	
Missing	35,476	*2.8*	30,327	*2.6*	27,885	*2.6*	2,442	*2.5*	
**Sex**									
Male	639,61	*51.1*	599,715	*51.2*	549,23	*51.1*	50,484	*51.6*	p=0.002
Female	610,87	*48.8*	571,075	*48.7*	523,88	*48.8*	47,190	*48.3*	
**Mother UK born**									
No	274,65	*21.9*	259,171	*22.1*	237,66	*22.1*	21,503	*22.0*	p=0.413
Yes	974,06	*77.8*	909,961	*77.7*	833,94	*77.7*	76,022	*77.8*	
Missing	1,766	*0.14*	1,658	*0.1*	1,509	*0.1*	149	*0.2*	
**Registration status**									
Married	716,85	*57.3*	665,764	*56.8*	612,06	*57.0*	53,697	*54.9*	p<0.000
Sole registrant	85,500	*6.8*	81,080	*6.93*	74,158	*6.9*	6,922	*7.1*	
Partner, same address	339,64	*27.1*	320,098	*27.3*	292,33	*27.2*	27,762	*28.4*	
Partner, different	108,49	*8.7*	103,848	*8.9*	94,556	*8.8*	9,293	*9.5*	
**Region of birth**									
East Midlands	84,901	*6.8*	82,311	*7.03*	77,750	*7.3*	4,561	*4.7*	p<0.000
East of England	123,46	*9.9*	118,491	*10.1*	106,96	*10.0*	11,530	*11.8*	
London	241,67	*19.3*	228,980	*19.5*	208,22	*19.4*	20,756	*21.2*	
North East	59,313	*4.74*	57,491	*4.9*	54,569	*5.1*	2,922	*3.0*	
North West	165,91	*13.2*	159,859	*13.6*	142,85	*13.3*	17,003	*17.4*	
South Central	86,355	*6.9*	83,610	*7.1*	78,451	*7.3*	5,159	*5.3*	
South East Coast	93,956	*7.5*	90,875	*7.8*	83,956	*7.8*	6,921	*7.1*	
South West	104,42	*8.4*	101,120	*8.6*	98,438	*9.2*	2,682	*2.8*	
West Midlands	134,12	*10.7*	129,223	*11.0*	108,06	*10.0*	21,160	*21.6*	
Yorkshire/Humber	122,33	*9.8*	118,830	*10.1*	113,85	*10.6*	4,980	*5.1*	

[a]*chi-square test: linked vs. unlinked*

All live births = all live births within the ONS birth cohort

All eligible births = all live, singleton, NHS hospital births to mothers living in England within the ONS birth cohort

Linked dataset = all eligible births from ONS birth cohort that successfully linked with a HES birth record

Unlinked dataset = all eligible births from ONS birth cohort that did not successfully link with a HES birth record

For peer review only

For peer review only - http://bmjopen.bmj.com/site/about/guidelines.xhtml

Table 4 cont. Comparison of all live, all eligible, all inked and all unlinked datasets

	All live births (n=1,250,490)		All eligible births (n=1,170,970)		Linked dataset (n=1,073,114)		Unlinked (n=97,674)		
	n	%	n	%	n	%	n	%	p-value[a]
**Month of birth**									
January	100,604	*8.1*	94,233	*8.1*	85,746	*8.0*	8487	*8.7*	p<0.0001
February	93,456	*7.5*	87,683	*7.5*	78,333	*7.3*	9350	*9.6*	
March	104,069	*8.3*	97,526	*8.3*	85,779	*8.0*	11747	*12.0*	
April	99,977	*8.0*	93,633	*8.0*	86,700	*8.1*	6933	*7.1*	
May	104,992	*8.4*	98,313	*8.4*	90,856	*8.5*	7455	*7.6*	
June	105,124	*8.4*	98,149	*8.4*	90,082	*8.4*	8070	*8.3*	
July	108,249	*8.7*	101,330	*8.6*	93,908	*8.8*	7421	*7.6*	
August	109,968	*8.8*	103,229	*8.8*	95,674	*8.9*	7555	*7.7*	
September	109,717	*8.8*	102,752	*8.8*	94,918	*8.9*	7833	*8.0*	
October	108,485	*8.7*	101,301	*8.6*	94,421	*8.8*	6881	*7.0*	
November	102,517	*8.2*	95,848	*8.2*	87,938	*8.2*	7910	*8.1*	
December	103,332	*8.3*	96,793	*8.3*	88,759	*8.3*	8032	*8.2*	
**Birth weight (g)**									
<1500	15,130	*1.2*	11,073	*1.0*	10,016	*0.9*	1056	*1.1*	p<0.0001
1500-1999	19,282	*1.5*	13,439	*1.2*	12,454	*1.2*	985	*1.0*	
2000-2499	60,012	*4.8*	47,825	*4.1*	44,089	*4.1*	3738	*3.8*	
2500-2999	213,073	*17.0*	195,554	*16.7*	179,236	*16.7*	16319	*16.7*	
3000-3499	447,030	*35.8*	427,600	*36.5*	391,926	*36.5*	35675	*36.5*	
3500-3999	357,505	*28.6*	342,606	*29.3*	314,060	*29.3*	28540	*29.2*	
4000-4499	116,484	*9.3*	111,708	*9.5*	102,534	*9.6*	9176	*9.4*	
4500+	20,730	*1.7*	19,940	*1.7*	18,328	*1.7*	1611	*1.7*	
Missing	1,244	*0.1*	1,045	*1.0*	471	*0.0*	574	*0.6*	
**Gestational age**									
<28	6,071	*0.5*	4,661	*0.4*	4,061	*0.4*	600	*0.6*	p<0.0001
28	2,035	*0.2*	1,524	*0.1*	1,382	*0.1*	142	*0.2*	
29	2,333	*0.2*	1,739	*0.2*	1,602	*0.2*	137	*0.1*	
30	2,999	*0.2*	2,261	*0.2*	2,071	*0.2*	189	*0.2*	
31	3,816	*0.3*	2,791	*0.2*	2,570	*0.2*	221	*0.2*	
32	5,367	*0.4*	3,820	*0.3*	3,509	*0.3*	311	*0.3*	
33	7,520	*0.6*	5,581	*0.5*	5,193	*0.5*	388	*0.4*	
34	12,261	*1.0*	9,225	*0.8*	8,506	*0.8*	718	*0.7*	
35	18,049	*1.4*	14,170	*1.2*	13,049	*1.2*	1122	*1.2*	
36	33,701	*2.7*	27,535	*2.4*	25,271	*2.4*	2264	*2.3*	
37	71,643	*5.7*	62,034	*5.3*	56,917	*5.3*	5117	*5.2*	
38	168,887	*13.5*	156,200	*13.3*	142,975	*13.3*	13226	*13.5*	
39	272,469	*21.8*	260,443	*22.3*	238,741	*22.3*	21707	*22.2*	
40	337,793	*27.0*	322,748	*27.6*	296,399	*27.6*	26347	*27.0*	
41	242,264	*19.4*	234,547	*20.0*	215,217	*20.1*	19325	*19.8*	
42	48,240	*3.9*	47,237	*4.0*	43,521	*4.1*	3716	*3.8*	
43+	5,063	*0.4*	4,893	*0.4*	4,578	*0.4*	315	*0.3*	
Missing	9,979	*0.8*	9,381	*0.8*	7,552	*0.7*	1829	*1.9*	

[a]chi-square test: linked vs. unlinked

All live births = all live births within the ONS birth cohort

All eligible births = all live, singleton, NHS hospital births to mothers living in England within the ONS birth cohort

Linked dataset = all eligible births from ONS birth cohort that successfully linked with a HES birth record

Unlinked dataset = all eligible births from ONS birth cohort that did not successfully link with a HES birth record

For peer review only

Discussion

Key findings

Our study has shown that linkage between ONS births and HES births based on NHS number; baby's date of birth, sex and postcode is possible. Overall, 92% of ONS birth records for live, singleton births born in NHS hospitals to mothers living in England were successfully linked to a HES birth record. After checking the quality of linkage in two stages, 1,456 records (<1%) were identified as potentially incorrect links. The main reasons for breaking links were different dates of birth and large amounts of data missing from key fields, making it difficult to determine if the link was correct. A higher proportion of links was broken for births in 2005 compared to births in 2006, suggesting an improvement in data quality between the two years. The addition of partially matching date of birth in the quality assurance algorithm increased sensitivity and identified more correct linkages, which would have otherwise been missed from the study cohort.

Duplicate HES records and large amounts of data missing for some variables created challenges when assessing linkage quality, highlighting the importance of these procedures before beginning statistical analysis. Finally, unlinked birth records were more likely to be unlinked if babies were of white, British ethnic origin born outside marriage, born in East England, London, North West England or the West Midlands or born in March.

Strengths and limitations

Key strengths of this study included building upon previous work conducted into the linkage of mothers' hospital records with babies' birth records, obtaining data from multiple sources, and the use of personal identifiers, which allowed us to evaluate the linkage more easily. The need to access personal identifiers also increased the problems involved in accessing the data, notably in terms of the length of approvals processes. Other limitations include the restriction of the assessment of linkage to live, singleton births in 2005 and 2006. This means that it is unclear how well the algorithms designed for this study would perform with multiple births. These tend to have more complex data, although similar algorithms were successfully used for the quality of assurance of the linkage of multiple births to mothers' delivery records[6]. In addition, the algorithms used in this study were deterministic, rather than probabilistic. The latter can be more effective when dealing with complex records, such as those with a lot of missing data or coding errors[15,16].

Interpretation of findings

Approximately 8% of ONS birth records did not link to a HES birth record. One of the key reasons for unlinked records appeared to be missing data in key linking fields. Quality and completeness is always a concern when using routinely collected datasets and the large sample sizes they provide are always traded off with these limitations. Data quality was somewhat poorer in HES birth records than in ONS birth records and this is a commonly cited issue when working with HES data[17,18]. In studies using HES birth data in later years, the data quality improves with time[17]. Therefore, researchers who wish to link the remaining years (2007 onwards) should achieve a higher linkage rate with better quality linkage. However, in previous work with mother's HES delivery records, the linkage rate plateaued at around 98% between 2010 and 2014 for singleton births, whereas the linkage rate in multiples began to decrease from 2010[6].

For peer review only - http://bmjopen.bmj.com/site/about/guidelines.xhtml

Compared to the full linked dataset, records of babies born in East England, North West England, London or the West Midlands were more likely to be unlinked. It is likely that these variations are due to differences between hospital trusts in the ways in which definitions or protocols are used or because errors occur during the transfer of data from one organisation to another or in the overall quality of Maternity HES. It is also possible that the regional reporting differences account for the higher proportion of white babies and births outside marriage in the unlinked dataset. Babies born in March were also more likely to have unlinked records and this was also the case with the mothers' HES delivery records[6]. HES uses financial years (1st April – 31st March) for report, so, differences in reporting standards prior to the financial year-end may account for this.

We found a small proportion of records that were potentially incorrect linkages, suggesting that linkage performed by 'Trusted Third Parties', such as NHS Digital, may not be 100% accurate. Therefore, it is essential that researchers using Trusted Third Party linked data evaluate the quality of linkage prior to performing any statistical analysis. The Digital Economy Act 2017 is designed to enable research for public benefit through the sharing of data, but the legislation is limited to the sharing of data that has been de-identified. This means reliance on the Trusted Third Party model in research is likely to increase over time. It is still unclear how this effects health and social care data, especially as they are not covered by the Digital Economy Act.[4] If researchers do not have access to personal identifiers, quality assurance of the linkage will become more challenging. Therefore, the findings in this paper will offer some insight into the quality of linkage between ONS birth and HES birth records and be of use to other researchers using the same linkage. As the quality and completeness of HES improved over time, it is likely that the quality of linkage will also improved as a consequence, but the signs that the improvement in quality may not have been sustained is worrying.

Recommendations for future practice and research

A number of studies have shown that datasets can be linked using de-identified datasets and produce generalisable cohorts, therefore, future work could explore ways to quality assure linkage without using personal identifiers. This will increase the likelihood of easier data access, potentially reducing the time required to complete the linkage and cleaning of routinely collected datasets. Finally, it remains unclear whether the quality of linkage for multiples is comparable with live, singleton, NHS hospital births although quality assurance of the linkage of multiple births to mothers' delivery records suggested that it was better than had been initially assumed.[6].

Conclusions

By linking together administrative datasets, such as birth registration, birth notification and hospital admission data, it is possible to create more complete data about births which can then be linked to subsequent hospital admissions to form valuable longitudinal datasets.[5] If data are missing or there are coding errors and inconsistences, the resulting datasets can often be of poor quality. It is therefore essential that the quality of linkage is assessed prior to analysis. The work presented in this study provides a guide to steps taken to quality assure the linkage of births in England and may be of use to other researchers working with similar datasets. The

For peer review only - http://bmjopen.bmj.com/site/about/guidelines.xhtml

findings will be particularly useful for researchers working with the same dataset but without access to personal identifiers.

For peer review only

For peer review only - http://bmjopen.bmj.com/site/about/guidelines.xhtml

Funding

This work was funded by the Medical Research Council (MRC): MR/M01228X/1. The funder had no input into the study design, data analysis, interpretation of results, or writing of the manuscript. VC and MQ had full access to all the data in the study and final responsibility for the decision to submit for publication.

Contributors

VC, MAQ and AJM were involved in the design of the study with input from Rod Gibson (Data management consultant) and Nirupa Dattani (Data analyst at City, University of London). RG and ND were involved in the cleaning of the ONS births dataset. VC performed the data manipulation and analysis. VC, AJM and MAQ were all involved in the interpretation of the data. VC was responsible for the initial draft of the manuscript. AJM and MAQ reviewed and contributed to drafts of the manuscript, and all authors have reviewed the final version.

Conflicts of interest

No conflicts of interest

Acknowledgements

We are grateful to the following organisations and individuals who were involved in this study: The MRC for funding this project (MR/M01228X/1); The Office for National Statistics (ONS) for providing access to data and hosting it within the Safe Research Setting (SRS); NHS Digital for linking and providing access to the data; Nirupa Dattani for providing her expertise on record linkage and data management; Rod Gibson, for his help cleaning the data; and Gillian Harper for her original work on the quality assurance of linkage of the mother's HES delivery records.

This work contains statistical data from ONS which is Crown Copyright. The use of the ONS statistical data in this work does not imply the endorsement of the ONS in relation to the interpretation or analysis of the statistical data. This work uses research datasets which may not exactly reproduce National Statistics aggregates.

For peer review only

References

1. Jorm, L. Routinely collected data as a strategic resource for research: priorities for methods and workforce. *Public Heal. Res. Pract.* **25**, 1–5 (2015).
2. Künn, S. The challenges of linking survey and administrative data. *IZA World Labor* (2015). doi:10.15185/izawol.214
3. House of Commons. *Digital Economy Act*. *The National Archives* (2017).
4. Bell, J. *et al.* Lawful disclosure of administrative data for research purposes in the UK. *J. Data Prot. Priv.* **2**, 264–277 (2019).
5. Macfarlane, A. *et al.* Births and their outcomes by time, day and year: a retrospective birth cohort data linkage study. *Heal. Serv. Deliv. Res.* **7**, (2019).
6. Harper, G. Linkage of Maternity Hospital Episode Statistics data to birth registration and notification records for births in England 2005-2014: Quality assurance of linkage of routine data for singleton and multiple births. *BMJ Open* **8**, (2018).
7. StataCorp. Stata Statistical Software: Release 14. (2015).
8. Hilder, L., Moser, K., Dattani, N. & Macfarlane, A. Pilot linkage of NHS Numbers for Babies data with birth registrations. *Heal. Stat. Q.* 25–33 (2007).
9. Herbert, A., Wijlaars, L., Zylbersztejn, A., Cromwell, D. & Hardelid, P. Data Resource Profile: Hospital Episode Statistics Admitted Patient Care (HES APC). *Int. J. Epidemiol.* **46**, 1093-1093i (2017).
10. HSCIC. *Methodology for creation of the HES Patient ID (HESID)*. (2014).
11. NHS DIGITAL. HES Data Dictionary - Admitted Patient. *HSCIC* (2018). Available at: file:///Users/victoriacoathup/Downloads/DD-APC-V11.pdf. (Accessed: 6th June 2018)
12. Dattani, N. & Macfarlane, A. Linkage of Maternity Hospital Episode Statistics data to birth registration and notification records for births in England 2005-2014: methods. A population-based birth cohort study. *BMJ Open* **8**, 1–7 (2018).
13. Herbert, A., Wijlaars, L., Zylbersztejn, A., Cromwell, D. & Hardelid, P. Data Resource Profile: Hospital Episode Statistics Admitted Patient Care (HES APC). *Int. J. Epidemiol.* **46**, 1093-1093i (2017).
14. Hagger-Johnson, G. *et al.* Identifying possible false matches in anonymized hospital administrative data without patient identifiers. *Health Serv. Res.* **50**, 1162–1178 (2015).
15. Paixão, E. S. *et al.* Evaluation of record linkage of two large administrative databases in a middle income country: Stillbirths and notifications of dengue during pregnancy in Brazil. *BMC Med. Inform. Decis. Mak.* **17**, 1–9 (2017).
16. Harron, K., Gilbert, R., Cromwell, D. & Van Der Meulen, J. Linking data for mothers and babies in de-identified electronic health data. *PLoS One* **11**, 1–18 (2016).
17. Murray, J. *et al.* Quality of routine hospital birth records and the feasibility of their use for creating birth cohorts. *J. Public Heal. (United Kingdom)* **35**, 298–307 (2013).
18. Ghosh, R. E. *et al.* Routinely collected English birth data sets: Comparisons and recommendations for reproductive epidemiology. *Arch. Dis. Child. Fetal Neonatal Ed.* **101**, F451–F457 (2016).

For peer review only - http://bmjopen.bmj.com/site/about/guidelines.xhtml

Figure Legends

Figure 1. Flowchart of study population

Figure S1. Overview of data sources

For peer review only

For peer review only - http://bmjopen.bmj.com/site/about/guidelines.xhtml

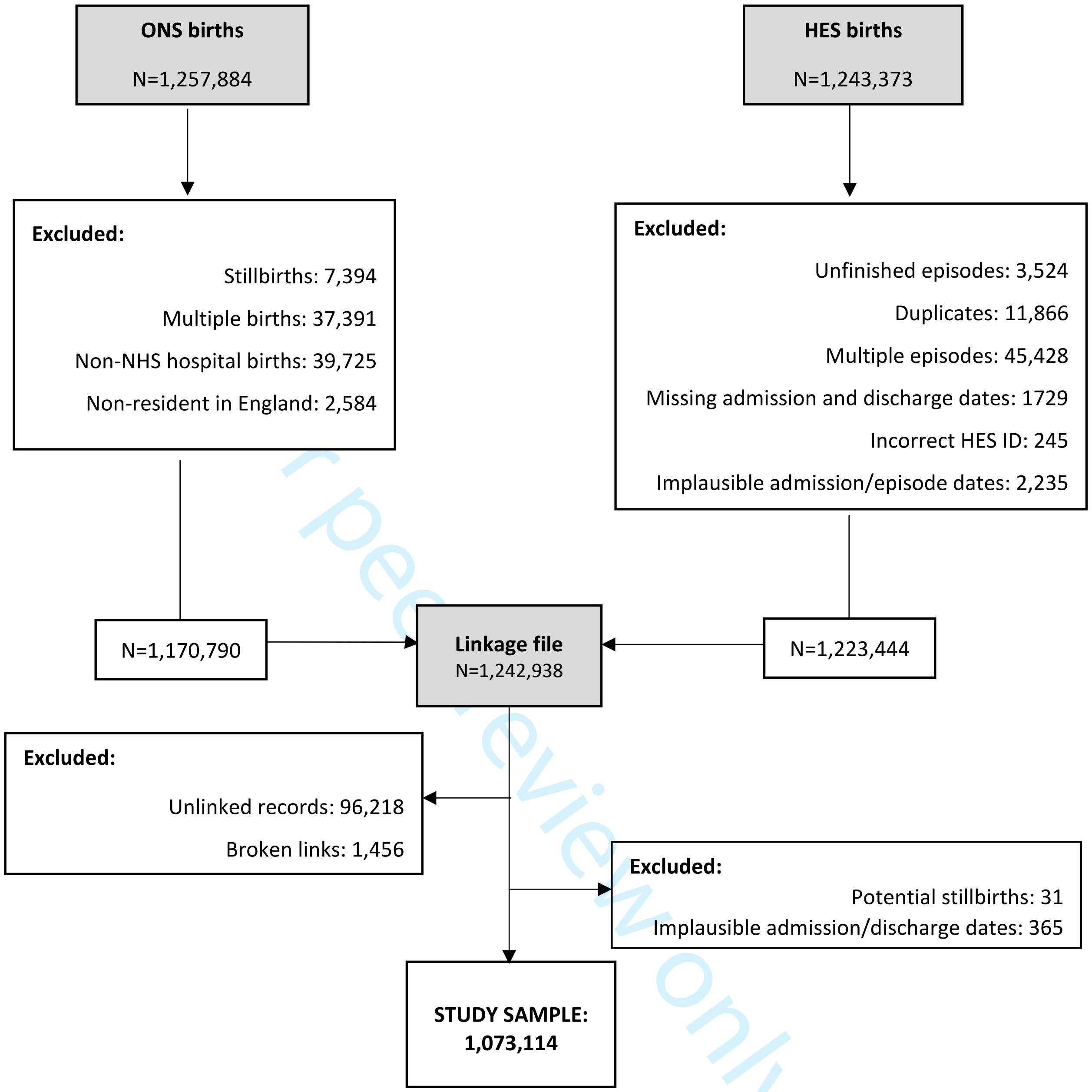
ONS births
N=1,257,884
HES births
N=1,243,373
Excluded:
Stillbirths: 7,394
Multiple births: 37,391
Non-NHS hospital births: 39,725
Non-resident in England: 2,584
Excluded:
Unfinished episodes: 3,524
Duplicates: 11,866
Multiple episodes: 45,428
Missing admission and discharge dates: 1729
Incorrect HES ID: 245
Implausible admission/episode dates: 2,235
N=1,170,790
Linkage file
N=1,242,938
N=1,223,444
Excluded:
Unlinked records: 96,218
Broken links: 1,456
Excluded:
Potential stillbirths: 31
Implausible admission/discharge dates: 365
STUDY SAMPLE:
1,073,114

Supplementary information

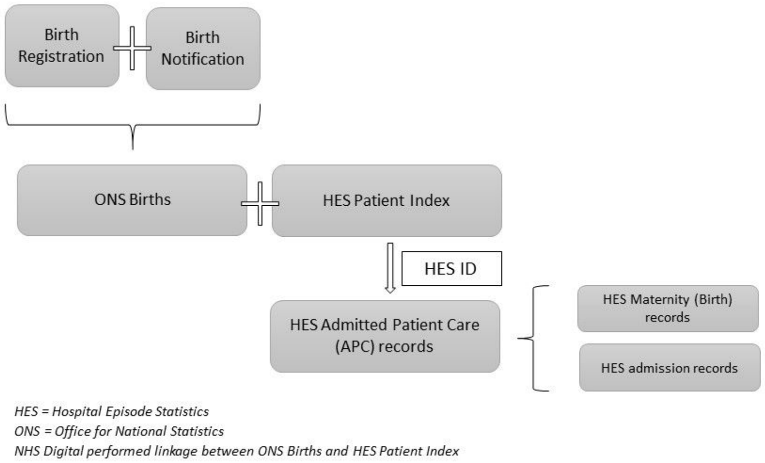

Figure S1. Overview of data sources

Definitions of Episode, Spell and Continuous Inpatient Stay (CIP)

HES inpatient admissions are structured as 'episodes' of care, with an episode defined as a period of care under one consultant or midwife. Multiple episodes are referred to as a 'spell' and represent an uninterrupted period of care within one hospital. A new spell is generated when the patient is transferred to another hospital to continue care. A continuous inpatient stay (CIP) may consist of one episode or multiple episodes and spells, and ends when the patient is discharged from an NHS hospital. Hospital episodes are primarily collected for financial reimbursement, and therefore, the datasets are divided into financial years, beginning 1st April and ending 31st March. Episodes are labelled as 'finished' once the patient is discharged from hospital. However, if an episode begins in one financial year and ends during the next, two episodes will be generated – one in the financial year the episode begins and one in the financial year that the episode ends – therefore, the first episode will remain as 'unfinished'.

- Unfinished episodes identified using episode type (EPITYPE = 1)
- Transfers identified using admission method, admission source and discharge destination (ADMIMETH = 81; ADMISORC = 49/50/51/52/53/87; or DISDEST = 49/50/51/52/53/84).

Table S1. NHS Digital algorithm used to link ONS Births with HES birth records

Match rank	Variables: NHS number	Variables: Date of birth	Variables: Sex	Variables: Postcode	Total: % Linked	Total: % Broken	2005: % Linked	2005: % Broken	2006: % Linked	2006: % Broken
1	E	E	E	E	*80.43*	*28.23*	*75.68*	*24.61*	*85.01*	*34.45*
2	E	E	E	-	*19.20*	*9.41*	*24.02*	*8.76*	*14.52*	*10.42*
3	E	P°	E	E	*0.02*	*1.31*	*0.01*	*1.35*	*0.02*	*1.59*
4	E	P°	E	-	*0.01*	*1.03*	*0.01*	*1.12*	*0.01*	*0.71*
5	E	-	-	E	*0.02*	*2.61*	*0.02*	*1.57*	*0.03*	*4.59*
6	M	E*	E	E^	*0.31*	*53.50*	*0.22*	*58.76*	*0.40*	*44.52*
7	M	E*	E	E	*0.01*	*1.10*	*0.01*	*1.35*	*0.01*	*0.68*
8	E	-	-	-	*0.02*	*2.82*	*0.03*	*2.47*	*0.01*	*3.13*
Number of NHS births					1,073,114	1,456	525,826	890	100.0	566

E=Exact match, P=Partial match, M=No match, due to missing data
°Two components of data match or DOB matches, when two components are swapped
**Date of birth not 1st January*
^Postcode not on 'ignore' list

For peer review only

Table S2. Sources of variables for quality assurance

Variable	Source	Rule
Baby's date of birth	BR & BN	Use BR unless BR = missing, then use BN
Mother's date of birth	BR & BN	Use BR unless BR = missing, then use BN
Birth weight	BR & BN	Use BR unless BR = missing, then use BN
Gestational age	BR & BN	use BN as BR reported for still births only
Sex	BR & BN	Use BR unless BR = missing, then use BN
Hospital of birth	BR & BN	Use BR unless BR = missing, then use BN

BR = Birth Registration

BN = Birth Notification (NN4B)

For peer review only

Table S3. Data preparation rules for quality assurance variables

Variable	Variable name: HES	Variable name: BR/BN	Rule	Additional checks
Baby's date of birth	DOBBABY	DOB/DOBZ	Set '01Jan1801' to missing. Make sure all dates are in format 'DDMMMYYYY' e.g. '01Jan2005'.	Check dates are not <01/01/05 or > 31/12/06
Mother's date of birth	MOTDOB	DOBM/DOBMZ	Set '01Jan1801' to missing. Make sure all dates are in format 'DDMMMYYYY' e.g. '01Jan2005'	
Postcode	HOMEADD	PCDRBZ (BN)	Transform var into format of 'XXX XXX', by removing any additional punctuation and transforming any lower case letters to upper case	
Birth weight	BIRWEIT	BTHWGTZ (BN)	Set '99', '9999', <400 and ≥7000 to missing	Check all hospitals report birth weight in g. If it looks like kg, then convert to equivalent in g.
Gestational age	GESTAT	GESTZ (BN)	Set '99' to missing	Check all hospital report gestational age in weeks, if not, convert from days or months to weeks
Sex	SEXBABY	SEXZ (BN)	Transform M to 1 and F to 2 if string variable. Set '0', '3' or '9' to missing.	
Hospital of birth	PROCODE	Location Code (derived from CESTRESS/ORGCODEZ)	Remove punctuation. Transform any lower case letters to upper case	

HES = Hospital Episode Statistics

BR = Birth Registration, BN= Birth Notification (NN4B)

For peer review only

Table S4. Percentages of data missing from each dataset for key variables used to evaluate linkage of data about live, singleton births in NHS hospitals to women living in England

	ONS Births			HES Births		
**Variable**	**Total**	**2005**	**2006**	**Total**	**2005**	**2006**
	%	%	%	%	%	%
NHS number	0.1	0.1	0.1	6.9	6.5	7.4
Baby's date of birth	0.0	0.0	0.0	0.0	0.0	0.0
Mother's date of birth	0.0	0.0	0.0	42.7	42.9	42.4
Mother's postcode	0.0	0.0	0.0	60.4	76.2	45.0
Hospital location code	0.0	0.0	0.0	0.0	0.0	0.0
Birthweight	0.1	0.1	0.1	44.4	43.8	45.1
Gestational age	0.8	0.8	0.8	49.1	45.7	52.6
Sex	0.0	0.0	0.0	0.0	0.0	0.0

For peer review only

Steps taken to deal with multiple HES birth records with the same HESID:

1. EPISTAT is 1 = unfinished episode: exclude all (for details of HES variables, see *NHS Digital Data Dictionary)*
2. HESID and EPIKEY exact match = duplicate record: keep any and exclude rest
3. HESID, DOB, Sex, hospital code, EPIORDER, ADMIDATE, DISDATE, EPISTART, EPIEND, DIAG_01 – DIAG_20, OPCS_01-OPCS_24, OPDATE_01-OPDATE_24 exact match and MDOB and POSTCODE either exact match or missing match = duplicate record: keep any and exclude rest
4. Two or more key variables (date of birth, Hospital code, mother's date of birth, postcode, birthweight) differ completely = unclear which record contains correct information: exclude all
5. Remainder are considered multiple episodes part of same spell = create new record using all available information from multiple episodes: keep one record and save other episodes in separate file
6. Dataset should now contain one HES birth record per baby

For peer review only

For peer review only

The RECORD statement – checklist of items, extended from the STROBE statement, that should be reported in observational studies using routinely collected health data.

	Item No.	STROBE items	Location in manuscript where items are reported	RECORD items	Location in manuscript where items are reported
**Title and abstract**					
	1	(a) Indicate the study's design with a commonly used term in the title or the abstract (b) Provide in the abstract an informative and balanced summary of what was done and what was found	Study design is included in the title and abstract	RECORD 1.1: The type of data used should be specified in the title or abstract. When possible, the name of the databases used should be included.<br><br>RECORD 1.2: If applicable, the geographic region and timeframe within which the study took place should be reported in the title or abstract.<br><br>RECORD 1.3: If linkage between databases was conducted for the study, this should be clearly stated in the title or abstract.	Type of data included in the title. Databases included in abstract<br><br>Region in title, region and timeframe mentioned in abstract<br><br>Stated in both the title and abstract
**Introduction**					
Background rationale	2	Explain the scientific background and rationale for the investigation being reported	Introduction		
Objectives	3	State specific objectives, including any prespecified hypotheses	At the end of the introduction		
**Methods**					
Study Design	4	Present key elements of study design early in the paper	Design and study population mentioned on page 3 at beginning of methods		

For peer review only

Setting	5	Describe the setting, locations, and relevant dates, including periods of recruitment, exposure, follow-up, and data collection	Described in study population section in methods		
Participants	6	*(a) Cohort study* - Give the eligibility criteria, and the sources and methods of selection of participants. Describe methods of follow-up<br>*Case-control study* - Give the eligibility criteria, and the sources and methods of case ascertainment and control selection. Give the rationale for the choice of cases and controls<br>*Cross-sectional study* - Give the eligibility criteria, and the sources and methods of selection of participants<br><br>*(b) Cohort study* - For matched studies, give matching criteria and number of exposed and unexposed<br>*Case-control study* - For matched studies, give matching criteria and the number of controls per case	Study population section within methods<br><br>N/A	RECORD 6.1: The methods of study population selection (such as codes or algorithms used to identify subjects) should be listed in detail. If this is not possible, an explanation should be provided.<br><br>RECORD 6.2: Any validation studies of the codes or algorithms used to select the population should be referenced. If validation was conducted for this study and not published elsewhere, detailed methods and results should be provided.<br><br>RECORD 6.3: If the study involved linkage of databases, consider use of a flow diagram or other graphical display to demonstrate the data linkage process, including the number of individuals with linked data at each stage.	Methods and supplementary information<br><br>N/A<br><br>Figure 1
Variables	7	Clearly define all outcomes, exposures, predictors, potential confounders, and effect modifiers. Give diagnostic criteria, if applicable.	N/A	RECORD 7.1: A complete list of codes and algorithms used to classify exposures, outcomes, confounders, and effect modifiers should be provided. If these cannot be reported, an explanation should be provided.	Supplementary information and appropriate references to sources of codes
Data sources/ measurement	8	For each variable of interest, give sources of data and details of methods of assessment (measurement).	Methods and supplementary information – but only key variables		

For peer review only

		Describe comparability of assessment methods if there is more than one group	with more than one source		
Bias	9	Describe any efforts to address potential sources of bias	Methods and supplementary information		
Study size	10	Explain how the study size was arrived at	Figure 1		
Quantitative variables	11	Explain how quantitative variables were handled in the analyses. If applicable, describe which groupings were chosen, and why	Outcomes, exposures and statistical analysis sections of methods		
Statistical methods	12	(a) Describe all statistical methods, including those used to control for confounding<br>(b) Describe any methods used to examine subgroups and interactions<br>(c) Explain how missing data were addressed<br>(d) *Cohort study* - If applicable, explain how loss to follow-up was addressed<br>*Case-control study* - If applicable, explain how matching of cases and controls was addressed<br>*Cross-sectional study* - If applicable, describe analytical methods taking account of sampling strategy<br>(e) Describe any sensitivity analyses	Assessment of linkage bias section in methods		
Data access and cleaning methods		..		RECORD 12.1: Authors should describe the extent to which the investigators had access to the database	Author contributions and Supplementary information

For peer review only

				population used to create the study population.<br><br>RECORD 12.2: Authors should provide information on the data cleaning methods used in the study.	
Linkage		..		RECORD 12.3: State whether the study included person-level, institutional-level, or other data linkage across two or more databases. The methods of linkage and methods of linkage quality evaluation should be provided.	Methods
**Results**					
Participants	13	(a) Report the numbers of individuals at each stage of the study (*e.g.*, numbers potentially eligible, examined for eligibility, confirmed eligible, included in the study, completing follow-up, and analysed)<br>(b) Give reasons for non-participation at each stage.<br>(c) Consider use of a flow diagram	Figure 1	RECORD 13.1: Describe in detail the selection of the persons included in the study (*i.e.,* study population selection) including filtering based on data quality, data availability and linkage. The selection of included persons can be described in the text and/or by means of the study flow diagram.	Figure 1
Descriptive data	14	(a) Give characteristics of study participants (*e.g.*, demographic, clinical, social) and information on exposures and potential confounders<br>(b) Indicate the number of participants with missing data for each variable of interest<br>(c) *Cohort study* - summarise follow-up time (*e.g.*, average and total amount)	Table 4 in results section		

For peer review only

Outcome data	15	*Cohort study* - Report numbers of outcome events or summary measures over time<br>*Case-control study* - Report numbers in each exposure category, or summary measures of exposure<br>*Cross-sectional study* - Report numbers of outcome events or summary measures	Table 4 in results section		
Main results	16	(a) Give unadjusted estimates and, if applicable, confounder-adjusted estimates and their precision (e.g., 95% confidence interval). Make clear which confounders were adjusted for and why they were included<br>(b) Report category boundaries when continuous variables were categorized<br>(c) If relevant, consider translating estimates of relative risk into absolute risk for a meaningful time period	N/A		
Other analyses	17	Report other analyses done—e.g., analyses of subgroups and interactions, and sensitivity analyses	N/A		
**Discussion**					
Key results	18	Summarise key results with reference to study objectives	First paragraph of discussion		
Limitations	19	Discuss limitations of the study, taking into account sources of potential bias or imprecision. Discuss both direction and magnitude of any potential bias	Strengths and limitations section of discussion	RECORD 19.1: Discuss the implications of using data that were not created or collected to answer the specific research question(s). Include discussion of misclassification bias, unmeasured confounding, missing data, and changing eligibility over	Strengths and limitations section of discussion

For peer review only

				time, as they pertain to the study being reported.	
Interpretation	20	Give a cautious overall interpretation of results considering objectives, limitations, multiplicity of analyses, results from similar studies, and other relevant evidence	Interpretation of findings section within the discussion		
Generalisability	21	Discuss the generalisability (external validity) of the study results	Interpretation of findings section within the discussion		
**Other Information**					
Funding	22	Give the source of funding and the role of the funders for the present study and, if applicable, for the original study on which the present article is based	Funding included at end of manuscript		
Accessibility of protocol, raw data, and programming code		..		RECORD 22.1: Authors should provide information on how to access any supplemental information such as the study protocol, raw data, or programming code.	Supplementary information

*Reference: Benchimol EI, Smeeth L, Guttmann A, Harron K, Moher D, Petersen I, Sørensen HT, von Elm E, Langan SM, the RECORD Working Committee. The REporting of studies Conducted using Observational Routinely-collected health Data (RECORD) Statement. *PLoS Medicine* 2015; in press.

*Checklist is protected under Creative Commons Attribution (CC BY) license.

BMJ Open

Linkage of Maternity Hospital Episode Statistics birth records to birth registration and notification records for births in England 2005-2006: Quality assurance of linkage

Journal:	*BMJ Open*
Manuscript ID	bmjopen-2020-037885.R1
Article Type:	Original research
Date Submitted by the Author:	18-Jun-2020
Complete List of Authors:	Coathup, Victoria; University of Oxford, Nuffield Department of Population Health<br>Macfarlane, Alison; City University, Dept of Midwifery<br>Quigley, Maria; Oxford University, National Perinatal Epidemiology Unit
**Primary Subject Heading**:	Health informatics
Secondary Subject Heading:	Epidemiology, Health services research, Public health, Research methods
Keywords:	PUBLIC HEALTH, Protocols & guidelines < HEALTH SERVICES ADMINISTRATION & MANAGEMENT, Clinical governance < HEALTH SERVICES ADMINISTRATION & MANAGEMENT

SCHOLARONE™
Manuscripts

I, the Submitting Author has the right to grant and does grant on behalf of all authors of the Work (as defined in the below author licence), an exclusive licence and/or a non-exclusive licence for contributions from authors who are: i) UK Crown employees; ii) where BMJ has agreed a CC-BY licence shall apply, and/or iii) in accordance with the terms applicable for US Federal Government officers or employees acting as part of their official duties; on a worldwide, perpetual, irrevocable, royalty-free basis to BMJ Publishing Group Ltd ("BMJ") its licensees and where the relevant Journal is co-owned by BMJ to the co-owners of the Journal, to publish the Work in this journal and any other BMJ products and to exploit all rights, as set out in our licence.

The Submitting Author accepts and understands that any supply made under these terms is made by BMJ to the Submitting Author unless you are acting as an employee on behalf of your employer or a postgraduate student of an affiliated institution which is paying any applicable article publishing charge ("APC") for Open Access articles. Where the Submitting Author wishes to make the Work available on an Open Access basis (and intends to pay the relevant APC), the terms of reuse of such Open Access shall be governed by a Creative Commons licence – details of these licences and which Creative Commons licence will apply to this Work are set out in our licence referred to above.

Other than as permitted in any relevant BMJ Author's Self Archiving Policies, I confirm this Work has not been accepted for publication elsewhere, is not being considered for publication elsewhere and does not duplicate material already published. I confirm all authors consent to publication of this Work and authorise the granting of this licence.

For peer review only

Linkage of Maternity Hospital Episode Statistics birth records to birth registration and notification records for births in England 2005-2006: Quality assurance of linkage

Dr Victoria Coathup[1], Professor Alison Macfarlane[2], Professor Maria Quigley[1]

[1]National Perinatal Epidemiology Unit (NPEU), University of Oxford, Oxford, UK

[2]Centre for Maternal and Child Health Research, School of Health Sciences, City, University of London, UK

Corresponding author

Victoria Coathup

National Perinatal Epidemiology Unit (NPEU)

Nuffield Department of Population Health

Old Road Campus

Oxford, OX3 7LF

victoria.coathup@npeu.ox.ac.uk

Abstract

Objectives: The objectives of this study were to describe the methods used to assess the quality of linkage between records of babies' birth registration records and hospital birth records, and to evaluate the potential bias that may be introduced because of these methods.

Design/Setting: Data from the civil registration and the notification of births previously linked by the Office for National Statistics (ONS) had been further linked to birth records from the Hospital Episode Statistics (HES) for babies born in England. We developed a deterministic, six stage algorithm to assess the quality of this linkage.

Participants: All 1,170,790 live, singleton births, occurring in NHS hospitals in England between 1st January 2005 and 31st December 2006.

Primary outcome measure: The primary outcome was the number of successful links between ONS birth records and HES birth records. Rates of successful linkage were calculated for the cohort and the characteristics associated with unsuccessful linkage were identified.

Results: Approximately 92% (1,074,572) of the birth registration records were successfully linked with a HES birth record. Data quality and completeness were somewhat poorer in HES birth records compared to linked birth registration and birth notification records. The quality assurance algorithms identified 1,456 incorrect linkages (<1%). Compared to the linked dataset, birth records were more likely to be unlinked if babies were of white ethnic origin; born to unmarried mothers; born in East England, London, North West England or the West Midlands; or born in March.

Conclusions: It is possible to link administrative datasets to create large cohorts allowing researchers to explore important questions about exposures and childhood outcomes. Missing data and coding errors and inconsistencies mean it is important that the quality of linkage is assessed prior to analysis.

Keywords

Births, hospital records, data-linkage, Hospital Episode Statistics

Strengths and limitations of this study

1. This is the first study to evaluate the linkage of birth registration, birth notification and HES birth records in England.
2. We were able to build on existing work relating to quality assurance of birth registration, birth notification and HES delivery records.
3. Access to personal identifiers meant we were able to evaluate the quality of linkage, identify poor quality matches and assess the level of bias introduced by linking these datasets.
4. Accessing personal identifiers also led to delays because of the length of approval processes and the need to travel to access these data in designated secure settings. However, the Office for National Statistics is developing a remote access system for the secure setting, so future research studies may not be affected by the need to travel .
5. We examined only singleton births in NHS hospitals so we did not assess whether the linkage of multiple births is of comparable quality. However, previous work linking birth registration records with mothers' delivery records in multiples suggests that the linkage rate is similar to that for singletons.

For peer review only

Introduction

The use of routinely collected datasets within research has increased rapidly over the past decade as an alternative to conducting large, observational studies, which can be extremely costly and often suffer from poor recruitment and retention rates[1]. Whilst there are many advantages to using linked administrative data for medical research, they also present challenges. One is the quality of data recorded and, in consequence, the quality of data linkage, as this is highly reliant upon the availability and accuracy of personal identifiers[2] and other supporting information.

The Digital Economy Act 2017[3] was introduced with the aim of facilitating data sharing for research purposes, but only if the data have been 'de-identified' and the research is deemed to be in the public interest[4]. Without access to personal identifiers, successful linkage between datasets becomes more challenging. Therefore, the 'Trusted Third Party' model, whereby the full identifiers are transferred to an organisation, which will link them with its own data and return the linked data to the data controller, is now the preferred method of linkage in most research projects. Whilst Trusted Third Parties typically publish their linkage algorithms, they usually do not publish results of quality assurance of their methods. Therefore, it is essential that researchers assess the quality of linkage and validity of data prior to conducting statistical analyses.

The work described in this paper was conducted as part of the (Tracking the Impact of Gestational Age on Health, Educational and Economic outcomes: a Longitudinal Records Linkage Study) TIGAR study, which is a population-based, record-linkage study of births and hospital admission data in England. The study aimed to estimate the association between gestational age at birth and rates of hospital admission throughout childhood. This used previously linked data from two sources.[5] The first of these was civil registration of births, a legal process in which parents register the birth and provide mainly demographic information to a specially trained local registrar of births, deaths and marriages who records the information, issues birth certificates and forwards the data to national systems. The second is the data recorded at the notification of the birth within 36 hours to the National Health Service (NHS) by the attending midwife or other birth attendant, and the baby's NHS Number, a unique identifier, is allocated. These combined ONS birth records were linked with birth records from Hospital Episode Statistics (HES), the national hospital discharge system for England. This was done by the data owner, the Health and Social Care Information Centre, now known as NHS Digital, as part of an earlier, larger study by City, University of London[6].

Two HES records are generated when a baby is born in England, one for the mother and one for each baby. Each consists of an Admitted Patient Care record common to all hospital in-patient stays plus a 'tail' with information about the birth. The mother's HES delivery record contains Admitted Patient Care information

For peer review only - http://bmjopen.bmj.com/site/about/guidelines.xhtml

relating to the mother's delivery and a 'maternity' tail'. The baby's HES birth record contains the baby's Admitted Patient Care record and a 'baby tail' containing details of the birth and overlapping extensively with the 'maternity tail'. Maternity HES data are downloaded from hospitals' administrative systems. As these do not all come from the same supplier, there are some differences in the ways in which data are entered and there are differences between systems and hospitals in the extent to which data items are missing.

The team at City, University of London has already evaluated the quality of linkage between birth registration, birth notification and the mother's HES delivery records for births from 2005 to 2014.The authors reported a linkage rate of 95% and uncovered some linkage errors[7]. Therefore, the main objectives of the current study were to assess the quality of linkage between baby's birth registration and notification records and the baby's HES birth records, and to evaluate the potential bias introduced to the study cohort by the linkage. This has relevance for analyses of similar linked administrative datasets.

Methods

All live, singleton babies born in NHS hospitals between 1st January 2005 and 31st December 2006 to a mother living in England were eligible for inclusion in this study cohort. The analysis was restricted to births in NHS hospitals, but they accounted for 96.6 per cent of women giving birth in 2006. Home births accounted for 2.7 per cent of deliveries in 2006, but although most received NHS care, many were not recorded on hospital systems and so not included in HES. There were extremely few HES records for the 0.5 per cent of deliveries in non-NHS hospitals and the 0.2 per cent delivering elsewhere.[8]

All analyses were conducted using STATA 14[9] within the Office for National Statistics (ONS)' Secure Research Service (SRS). An overview of the procedures involved in the quality assurance process for this study is presented in **Figure 1**.

Figure 1. Flow chart of study population

Data sources

Two datasets containing data about births born in England from 1st January 2005 to 31st December 2006 were linked (**Figure S1**). They and the linkage file were saved as three separate STATA datasets. The datasets included: (A) ONS Births; (B) Hospital Episode Statistics (HES) birth records; and (C) the Linkage file containing unique ONS birth identifiers (ONS_id) and corresponding unique HES identifiers (HESID). These files are described in the supplementary information (**Figure S1**).

A. ONS births

For peer review only - http://bmjopen.bmj.com/site/about/guidelines.xhtml

The master dataset comprises data from two sources: birth registration and birth notification These two datasets have been routinely linked by the ONS since 2006 and the combined dataset is referred to as 'ONS births' throughout this paper. ONS births contains personal identifiers, socio-demographic characteristics and birth characteristics.[10]

B. Hospital Episode Statistics (HES Birth records)

HES is a large database containing records of all episodes of care and births in NHS hospitals in England since 1989. The records used here are from the HES Admitted Patient Care (APC) dataset, which contains records of all inpatient admissions, including birth and delivery records, to NHS hospitals across England. A full description of the database can be found elsewhere.[11] Briefly, HES inpatient admissions are structured as 'episodes' of care, with an episode defined as a period of care under one consultant or midwife. Each episode contains details relating to the individual, care provider and care received (including diagnosis and procedural codes). If a patient receives care in more than one department, this generates multiple episodes, referred to as a 'spell' and represents an uninterrupted period of care within one hospital. A new spell is generated when the patient is transferred to a different hospital to continue care. A continuous inpatient stay (CIP) may consist of one or more episodes and spells, and ends when the patient is discharged from an NHS hospital. Data about hospital episodes are primarily collected for financial reimbursement, and therefore, the datasets are divided into financial years, beginning 1st April and ending 31st March. Episodes are labelled as 'finished' once the patient is discharged from hospital. However, if an episode begins in one financial year and ends during the next, two episodes will be generated; one in the financial year the episode begins and one in the financial year that the episode ends. In this case,, the first episode will be defined as 'unfinished'.

Everyone for whom records are stored in HES is assigned a unique identifier, called the HESID.[12] This is generated using a combination of NHS Number, local patient identifier, postcode, sex and date of birth to enable data users to uniquely track patients throughout the NHS. Descriptions of the variables available are in NHS Digital's HES Data Dictionary[13].

When a baby is born, the general inpatient episode becomes part of the Maternity HES dataset and 19 additional variables relating to the delivery or birth are appended. For each birth, two Maternity HES records are generated. Firstly, a HES delivery record, which includes the general inpatient record for the mother and 19 additional variables, referred to as the 'maternity tail'. Secondly, a HES birth record, which includes the general inpatient record for the baby, along with 19 additional variables, referred to as the 'baby tail'. The maternity and baby tails contain similar information relating to the delivery and birth; however, this study evaluated the

For peer review only - http://bmjopen.bmj.com/site/about/guidelines.xhtml

linkage of HES birth records only. These additional data items in the 'baby tail' include variables such as gestational age at birth and neonatal level of care. For a full list, please see section A in the supplementary information.

C. Linkage file

The linkage file contained the unique identifiers from each dataset as linked by NHS Digital. The dataset contained unique identifiers from ONS births that had been successfully linked with the corresponding unique identifier from HES births and those which had not. It also contained unique HES birth identifiers that were not linked to a unique ONS birth identifier.

Linkage of the Baby cohort and Maternity HES

Linkage between ONS birth records and HES birth records is a two-step process. Firstly, ONS birth records were linked to the HES patient index (an index of personal details relating to all individuals with access to NHS hospitals) by NHS Digital using a deterministic algorithm in order to assign the HESID; and secondly, records were linked to the corresponding HES birth records using the HESID and other identifiers.[5] Here, each linked record was assigned a match rank score, indicating the stage in the algorithm at which the records had been matched (1 being highest quality). Steps of the algorithm are summarised in **Table S1**.

Data preparation for quality assurance checks

Full details of available variables, data cleaning procedures and preparation steps for the included datasets have are described elsewhere.[6] Many key variables were available from both birth registration and birth notification. The preferred sources are summarised in the supplementary information (**Table S2)**. A number of additional steps were taken to ensure that key variables in HES births and ONS births were in consistent formats, ready for comparison during the quality assurance procedure (**Table S3**). Further checks were conducted on HES birth records and those with: (1) discharge date occurring before admission; (2) discharge occurring before baby's date of birth; or (3) admission dates occurring before 1st January 2005 or after 31st December 2006 were excluded.

Multiple episodes with the same HESID (i.e. duplicate HES birth records)

Birth episodes with the same HESID can occur due to a number of reasons: (1) unfinished episodes, such as a baby being born on the 30th March, but then discharged on the 1st April[14]; (2) administrative errors[7]; (3) a birth spell containing multiple episodes, such as a baby being transferred to a different hospital or consultant; and (4) HESID is incorrectly assigned to more than one baby[15]. The steps which were taken have been summarised in

For peer review only - http://bmjopen.bmj.com/site/about/guidelines.xhtml

For peer review only

the supplementary information. In addition to this, episode start dates that occurred more than two days after the previous episode ended and did not include a transfer code (see supplementary information) were considered a new admission and were saved separately.

Quality assurance of the baby cohort and Maternity HES linkage

After merging the three datasets, we identified cases where two different ONS birth records had been linked to the same HES birth record and key characteristics were compared to identify the correct link. This was done by comparing the following variables which were available in both data sources: baby's date of birth, gestational age, birth weight, sex, mother's date of birth and postcode. The record with the highest number of matching variables was identified as the correct link. When records matched on the same number of variables, the record that matched exactly on birthweight was identified as the correct match. If birthweight was missing or did not match with either record, then the record with the highest match rank score from the original linkage (Table S1) was identified as the correct link. The remaining records were then compared manually, but in cases where records had a high proportion of missing data or the same number of matching variables and the same match rank score, it was not possible to identify the correct link and therefore all were excluded.

The deterministic algorithm developed for evaluating the record linkage is summarised in **Table 1**. The quality assurance (QA) algorithm was adapted from the previous study which assessed the quality of linkage between ONS birth records and the mother's HES delivery records[7], and was based on location of birth, date of birth, sex, birthweight and gestational age. The location of birth was defined as the hospital trust running the hospital that the baby was born in. A hospital trust in the UK is an organisational unit within the NHS and usually refers to a group of hospitals that are in close proximity to each other. This was used instead of the hospital of birth to account for potential transfers between hospitals within the birth admission. The hospital trust variable was developed as part of a previous study[6].To account for differences in rounding between hospitals, a birthweight of + or – 100g was considered a match.

For peer review only - http://bmjopen.bmj.com/site/about/guidelines.xhtml

Table 1. Quality assurance algorithm to assess linkage of ONS birth records to HES birth records

	Variable												
**Step**	**Hospital Trust code**		**Date of birth**		**Sex**		**Birthweight**		**Gestational age**		**Mother's date of birth**		**Postcode**
1	E	*and*	E	*and*	E	*and*	E	*and*	E		-		-
2	E	*and*	E	*and*	E	*and*	(E	*or*	E)		-		-
3	E	*and*	E	*and*	E		-		-	*and*	E/M	*and*	E/M
4	-		E	*and*	E	*and*	E/M	*and*	E/M	*and*	E/M	*and*	E/M
5	E	*and*	P	*and*	E	*and*	(E	*or*	E	*or*	E	*or*	E)
6	E	*and*	P		-		E/M	*and*	E/M	*and*	E/M	*and*	E/M

E = Exact match; M = Missing; P = Partial match (differs by up to four days, two elements of data match or dates match if day and month swapped)

For peer review only

Final checks after the quality assurance procedures were completed included: (1) Checking that the baby's date of birth in ONS births was within the admission and discharge dates from the HES birth record; (2) Baby's date of birth in ONS births did not occur before 1st January 2005 or after 31st December 2006; (3) hospital discharge date did not occur before the admission date; and (4) further checks to ensure that all stillbirths had been excluded (ICD10 diagnosis code = Z37.1).

Assessment of linkage bias

The distributions of key characteristics were compared between the linked and unlinked study samples and the eligible study sample. The chi-squared test was used to assess whether distributions differed significantly between the linked and unlinked study samples. Because the sample sizes were so large we also looked at differences in distributions of variables, for example, differences between proportions of more than 1%. In addition to this, we compared the distributions of key characteristics between the linked study sample and all live births in England in 2005 and 2006 to see what effect excluding stillbirths, multiple and non-NHS births had on the study cohort.

Patient and Public Involvement

The TIGAR study was supported by a patient, parent and public advisory group, which provided input to different aspects of the study. This group met at the start of the study and gave input into the study protocol and the lay summary of the project.

Results

There were 1,257,884 ONS birth records. After excluding multiple births and babies who were not eligible for the TIGAR study, 1,170,790 live, singleton babies born from 1st January 2005 to 31st December 2006 in NHS hospitals to women living in England remained in the study cohort. There were 1,243,373 HES birth records and the linkage file included 1,242,938 records (**Figure 1**).

Linkage of baby cohort to Maternity HES

Of the 1,170,790 eligible ONS birth records, 1,074,571 or 92% were successfully linked with a HES birth record. Of the 96,219 unlinked records, 88,471 had a HESID but no corresponding HES birth record and 7,747 had no corresponding HESID in the linkage file. The majority of records linked had a match rank score of one, which meant they exactly matched on all four variables in the NHS Digital algorithm, with fewer than 1% of records linking in stages three to six of the algorithm (Table S1). A higher proportion of links had a match rank score of one in 2006 compared to 2005, however.

Data preparation for quality assurance checks

All key variables in ONS birth records had data missing for less than 1% of births. In contrast, data were missing from substantial numbers of HES birth records (**Table S4**).

For peer review only - http://bmjopen.bmj.com/site/about/guidelines.xhtml

Multiple birth episodes with the same HESID

Of the 1,243,373 HES birth records, 73,307 (6%) had linked with more than one record with the same HESID. Of these, the most common reasons for this were multiple episodes within a hospital spell (62%), unfinished episodes (15%) and duplicate episodes due to administrative errors (11%) (**Figure 1**). There were 1,197,999 unique HES birth records remaining.

Quality assurance of the baby cohort and Maternity HES linkage

A total of 979 (0.1%) HES birth records had linked with more than one ONS birth record. The high proportion of missing data in HES birth records meant it was difficult in many cases to identify the correct match. Of these, 499 records were judged to be incorrectly linked and were broken. It was not possible to ascertain the status of 95 records, which had the same matching variables and match rank score. A lot of data items in these records were missing data and all were excluded (**Table 2)**.

For peer review only

For peer review only - http://bmjopen.bmj.com/site/about/guidelines.xhtml

Table 2. Proportions of birth records at each quality assurance stage in 2005 and 2006 for live, singleton births born in NHS hospitals to mother's living in England

Quality assurance stage		Total	2005		2006	
		n	n	%	n	%
1	Number of Births*	1,170,790	575,568	*49.2*	595,222	*50.8*
2	Number of ONS Birth records linked to HESID	1,148,110	563,963	*49.1*	584,147	*50.9*
3	Number of ONS Birth records not linked to HESID	22,680	11,605	*51.2*	11,075	*48.8*
4	Number of ONS Birth records linked to HESID and HES birth record	1,074,571	530,586	*49.4*	543,985	*50.6*
5	Number of ONS Birth records linked to HESID but not linked to HES birth record	96,219	44,982	*46.7*	51,237	*53.3*
6	Number of ONS Birth records linked to one HES birth record before QA	1,074,571	530,586	*49.4*	543,985	*50.6*
7	Number of links broken between ONS Birth records and HES birth records after QA	1,456	890	*61.1*	566	*38.9*
8	Number of ONS Birth records linked to one HES birth record after QA	1,073,115	530,119	*49.4*	542,996	*50.6*
-	% of total ONS birth records left linked to one HES birth record after QA	91.7	-	*92.1*	-	*91.3*
9	Total number of ONS Birth records not linked to HES birth record after QA	97,675	45,810	*46.9*	51,865	*53.1*
-	% of total ONS birth records not linked to HES birth record after QA	8.3	-	*7.9*	-	*8.7*

**Birth registration linked to birth notification records of live, singleton births that occurred in NHS hospitals to women residing in England*

For peer review only

For peer review only - http://bmjopen.bmj.com/site/about/guidelines.xhtml

Key variables of the 1,074,571 (92%) ONS birth records, which had been successfully linked, were then compared using the algorithm in **Table 1**. The vast majority (99.5%) of correct links were found to be within the first three stages of the quality assurance algorithm (**Table 3**). The 645 records identified as correct links in stage four of the algorithm appeared to be correct matches; the hospital location code differed slightly, which may have resulted from either a transfer to a neighbouring hospital trust during the birth admission or from data entry errors. In stage five, 1,973 (0.2%) records were identified as correct links. Of these, 76% had a match rank score of one, suggesting the date of birth had been entered incorrectly in the HES birth record. The majority of these matches looked like data entry errors where the month and day had inadvertently been swapped. In stage six of the algorithm, a small number of records were identified as correct links. These records had a lot of data missing, including 64% of birthweights, 70% of gestational ages, 77% of postcodes; and 67% of mother's dates of birth. Of the 242 records with partially matching dates of births, 91% had a match rank score of one or two, suggesting good quality matches but with data entry errors in the HES birth records. Interestingly, almost 62% of records in this stage exactly matched on date of birth, but differed by sex. Most of these records also exactly matched on birthweight, gestational age, postcode and mother's date of birth, suggesting they are correct links, again with data entry errors in the HES birth record.

Table 3. Proportion of birth records identified as correct links at each stage of the quality assurance process

**Step**	**Total**		**2005**		**2006**	
	Number	%	Number	%	Number	%
**1**	385,570	*35.9*	207,106	*39.1*	178,464	*32.8*
**2**	301,066	*28.0*	134,684	*25.4*	166,382	*30.6*
**3**	383,189	*35.6*	186,203	*35.1*	196,986	*36.2*
**4**	645	*0.1*	426	*0.1*	219	*0.0*
**5**	1,973	*0.2*	983	*0.2*	990	*0.2*
**6**	671	*0.0*	379	*0.0*	292	*0.0*
**Poor quality links**	**860**	***0.1***	**548**	***0.1***	**312**	***0.1***
**All**	**1,074,571**	***100***	**530,586**	***100***	**543,985**	***100***

Overall, 860 records with incorrect links were identified and 64% of these were in births in 2005, suggesting an improvement in data and linkage quality in 2006. When exploring these broken links in relation to their match rank score, 69% had a score of six, meaning their NHS numbers were missing. The main reason for broken links was completely different dates of birth as opposed to exact or partial matching and data missing for large numbers of variables. Among these records, birthweight, gestational age, postcode and mother's date of birth was missing for approximately 92%, 91%, 85% and 91%, respectively. There were a small number of records that appeared to be incorrectly broken due to cleaning errors, for example postcodes which included an 'O' instead of a zero.

A number of interesting observations was made when reviewing the broken links: (1) Many that were broken differed by gestational age, but in most cases by just one week and the babies were full term for example 39 and

For peer review only - http://bmjopen.bmj.com/site/about/guidelines.xhtml

40 weeks; and (2) More than one third of broken links were for babies born in London. As the numbers of broken links were small, distributions of numbers of broken links cannot be presented, as they could be disclosive.

Assessment of linkage bias

Distributions of key variables for all live births, all eligible births, and all linked and all unlinked births are presented in **Table 4**. Compared to the linked dataset, the distribution of those in the unlinked dataset differed by baby's ethnicity, mother's age, parity, Index of Multiple Deprivation score (IMD) score, sex, registration status, region and month of birth, birth weight and gestational age ($p<0.05$). Although absolute differences were small (<1%) for most variables, there were some noticeable discrepancies, where distributions differed by more than 1%. Unlinked records were more likely occur if babies were of white ethnic origin; born outside marriage; born in East England, London, North West England or the West Midlands; or born in March. Compared to all eligible births in 2005 and 2006, the distributions of linked births were very similar and differed by less than 1% in all cases.

For peer review only - http://bmjopen.bmj.com/site/about/guidelines.xhtml

Table 4. Comparison of all live, all eligible, all inked and all unlinked datasets

**Characteristics**	**All live births (n=1,250,490)** n	%	**All eligible (n=1,170,790)** n	%	**Linked (n=1,073,115)** n	%	**Unlinked (n=97,675)** n	%	p-value[a]
**Ethnicity**									
Bangladeshi	16,730	*1.3*	16,281	*1.4*	15,356	*1.4*	925	*1.0*	p<0.000
Indian	32,241	*2.6*	31,287	*2.7*	28,970	*2.7*	2,318	*2.4*	
Pakistani	48,570	*3.9*	47,352	*4.0*	43,702	*4.1*	3,648	*3.7*	
Black African	24,461	*2.0*	23,394	*2.0*	21,504	*2.0*	1,888	*1.9*	
Black Caribbean	8,668	*0.7*	8,200	*0.7*	7,420	*0.7*	781	*0.8*	
White British	788,985	*63.0*	738,88	*63.1*	675,34	*62.9*	63,552	*65.1*	
White Other	91,311	*7.3*	86,918	*7.4*	80,611	*7.5*	6,306	*6.5*	
Other	102,048	*8.2*	97,362	*8.3*	89,043	*8.3*	8,318	*8.5*	
Missing	137,476	*11.0*	121,10	*10.3*	111,16	*10.4*	9,939	*10.2*	
**Mother's age**									
Under20	52,605	*4.2*	51,312	*4.4*	46,803	*4.4*	4,509	*4.6*	p<0.000
20-24	216,768	*17.3*	208,68	*17.8*	190,72	*17.8*	17,960	*18.4*	
25-29	306,603	*24.5*	290,13	*24.8*	266,00	*24.8*	24,130	*24.7*	
30-34	363,312	*29.1*	337,32	*28.8*	309,60	*28.9*	27,718	*28.4*	
35-39	244,952	*19.6*	223,25	*19.1*	204,99	*19.1*	18,263	*18.7*	
40+	66,250	*5.3*	60,079	*5.1*	54,987	*5.1*	5095	*5.2*	
**Nulliparous**									
No	587,834	*47.0*	562,20	*48.0*	523,05	*48.7*	39,152	*40.1*	p<0.000
Yes	570,129	*45.5*	548,25	*46.8*	505,98	*47.2*	42,279	*43.3*	
Missing	92,527	*7.4*	60,325	*5.2*	44,083	*4.1*	16,244	*16.6*	
**IMD Score (quintiles)**									
Q1	333,809	*26.7*	318,83	*27.2*	292,31	*27.2*	26,513	*27.1*	p=0.001
Q2	263,212	*21.1*	247,77	*21.2*	227,08	*21.2*	20,700	*21.2*	
Q3	221,720	*17.7*	206,64	*17.7*	189,75	*17.7*	16,891	*17.3*	
Q4	200,891	*16.1*	186,16	*15.9*	170,31	*15.9*	15,851	*16.2*	
Q5	195,382	*15.6*	181,04	*15.5*	165,76	*15.5*	15,277	*15.6*	
Missing	35,476	*2.8*	30,327	*2.6*	27,886	*2.6*	2,443	*2.5*	
**Sex**									
Male	639,617	*51.2*	599,71	*51.2*	549,23	*51.2*	50,484	*51.7*	p=0.002
Female	610,873	*48.9*	571,07	*48.8*	523,88	*48.8*	47,191	*48.3*	
**Mother UK born**									
No	274,659	*22.0*	259,17	*22.1*	237,66	*22.2*	21,504	*22.0*	p=0.413
Yes	974,065	*77.9*	909,96	*77.7*	833,94	*77.7*	76,022	*77.8*	
Missing	1,766	*0.1*	1,658	*0.1*	1,509	*0.1*	149	*0.2*	
**Registration status**									
Married	716,858	*57.3*	665,76	*56.9*	612,06	*57.0*	53,698	*55.0*	p<0.000
Sole registrant	85,500	*6.8*	81,080	*6.9*	74,158	*6.9*	6,922	*7.1*	
Partner, same address	339,641	*27.2*	320,09	*27.3*	292,33	*27.2*	27,762	*28.4*	
Partner, different	108,491	*8.7*	103,84	*8.9*	94,556	*8.8*	9,293	*9.5*	
**Region of birth**									
East Midlands	87,310	*7.0*	82,311	*7.0*	77,750	*7.3*	4,561	*4.7*	p<0.000
East of England	126,925	*10.1*	118,49	*10.1*	106,96	*10.0*	11,530	*11.8*	
London	248,341	*19.9*	228,98	*19.6*	208,22	*19.4*	20,756	*21.3*	
North East	61,007	*4.9*	57,491	*4.9*	54,569	*5.1*	2,922	*3.0*	
North West	170,504	*13.6*	159,85	*13.7*	142,85	*13.3*	17,003	*17.4*	
South Central	88,798	*7.1*	83,610	*7.1*	78,451	*7.3*	5,159	*5.3*	
South East Coast	96,606	*7.7*	90,875	*7.8*	83,956	*7.8*	6,921	*7.1*	
South West	107,376	*8.6*	101,12	*8.6*	98,438	*9.2*	2,682	*2.8*	
West Midlands	137,859	*11.0*	129,22	*11.0*	108,06	*10.1*	21,160	*21.7*	
Yorkshire/Humber	125,765	*10.1*	118,83	*10.2*	113,85	*10.6*	4,981	*5.1*	

[a]*chi-square test: linked vs. unlinked*

All live births *= all live births within the ONS birth cohort*

All eligible births *= all live, singleton, NHS hospital births to mothers living in England within the ONS birth cohort*

Linked dataset *= all eligible births from ONS birth cohort that successfully linked with a HES birth record*

Unlinked dataset *= all eligible births from ONS birth cohort that did not successfully link with a HES birth record*

For peer review only

Table 4 cont. Comparison of all live, all eligible, all inked and all unlinked datasets

Characteristics	All live births (n=1,250,490)		All eligible births (n=1,170,970)		Linked dataset (n=1,073,115)		Unlinked (n=97,675)		
	n	%	n	%	n	%	n	%	p-value[a]
**Month of birth**									
January	100,604	*8.1*	94,233	*8.1*	85,746	*8.0*	8,487	*8.7*	p<0.0001
February	93,456	*7.5*	87,683	*7.5*	78,333	*7.3*	9,350	*9.6*	
March	104,069	*8.3*	97,526	*8.3*	85,779	*8.0*	11,747	*12.0*	
April	99,977	*8.0*	93,633	*8.0*	86,701	*8.1*	6,933	*7.1*	
May	104,992	*8.4*	98,313	*8.4*	90,856	*8.5*	7,455	*7.6*	
June	105,124	*8.4*	98,149	*8.4*	90,082	*8.4*	8,071	*8.3*	
July	108,249	*8.7*	101,330	*8.6*	93,908	*8.8*	7,421	*7.6*	
August	109,968	*8.8*	103,229	*8.8*	95,674	*8.9*	7,555	*7.7*	
September	109,717	*8.8*	102,752	*8.8*	94,918	*8.9*	7,833	*8.0*	
October	108,485	*8.7*	101,301	*8.6*	94,421	*8.8*	6,881	*7.0*	
November	102,517	*8.2*	95,848	*8.2*	87,938	*8.2*	7,910	*8.1*	
December	103,332	*8.3*	96,793	*8.3*	88,759	*8.3*	8,032	*8.2*	
**Birth weight (g)**									
<1500	15,130	*1.2*	11,073	*1.0*	10,016	*0.9*	1,056	*1.1*	p<0.0001
1500-1999	19,282	*1.5*	13,439	*1.2*	12,454	*1.2*	985	*1.0*	
2000-2499	60,012	*4.8*	47,825	*4.1*	44,089	*4.1*	3,738	*3.8*	
2500-2999	213,073	*17.0*	195,554	*16.7*	179,236	*16.7*	16,319	*16.7*	
3000-3499	447,030	*35.8*	427,600	*36.5*	391,926	*36.5*	35,675	*36.5*	
3500-3999	357,505	*28.6*	342,606	*29.3*	314,060	*29.3*	28,540	*29.2*	
4000-4499	116,484	*9.3*	111,708	*9.5*	102,534	*9.6*	9,176	*9.4*	
4500+	20,730	*1.7*	19,940	*1.7*	18,328	*1.7*	1,611	*1.7*	
Missing	1,244	*0.1*	1,045	*1.0*	472	*0.0*	575	*0.6*	
**Gestational age**									
<28	6,071	*0.5*	4,661	*0.4*	4,061	*0.4*	600	*0.6*	p<0.0001
28	2,035	*0.2*	1,524	*0.1*	1,382	*0.1*	142	*0.2*	
29	2,333	*0.2*	1,739	*0.2*	1,602	*0.2*	137	*0.1*	
30	2,999	*0.2*	2,261	*0.2*	2,071	*0.2*	189	*0.2*	
31	3,816	*0.3*	2,791	*0.2*	2,570	*0.2*	221	*0.2*	
32	5,367	*0.4*	3,820	*0.3*	3,509	*0.3*	311	*0.3*	
33	7,520	*0.6*	5,581	*0.5*	5,193	*0.5*	388	*0.4*	
34	12,261	*1.0*	9,225	*0.8*	8,506	*0.8*	719	*0.7*	
35	18,049	*1.4*	14,170	*1.2*	13,049	*1.2*	1,122	*1.2*	
36	33,701	*2.7*	27,535	*2.4*	25,271	*2.4*	2,264	*2.3*	
37	71,643	*5.7*	62,034	*5.3*	56,917	*5.3*	5,117	*5.2*	
38	168,887	*13.5*	156,200	*13.3*	142,975	*13.3*	13,226	*13.5*	
39	272,469	*21.8*	260,443	*22.3*	238,741	*22.3*	21,707	*22.2*	
40	337,793	*27.0*	322,748	*27.6*	296,399	*27.6*	26,347	*27.0*	
41	242,264	*19.4*	234,547	*20.0*	215,217	*20.1*	19,325	*19.8*	
42	48,240	*3.9*	47,237	*4.0*	43,521	*4.1*	3,716	*3.8*	
43+	5,063	*0.4*	4,893	*0.4*	4,578	*0.4*	315	*0.3*	
Missing	9,979	*0.8*	9,381	*0.8*	7,553	*0.7*	1,829	*1.9*	

[a]chi-square test: linked vs. unlinked

***All live births** = all live births within the ONS birth cohort*

***All eligible births** = all live, singleton, NHS hospital births to mothers living in England within the ONS birth cohort*

***Linked dataset** = all eligible births from ONS birth cohort that successfully linked with a HES birth record*

***Unlinked dataset** = all eligible births from ONS birth cohort that did not successfully link with a HES birth record*

For peer review only - http://bmjopen.bmj.com/site/about/guidelines.xhtml

Discussion

Key findings

Our study has shown that linkage between ONS births and HES births based on NHS number; baby's date of birth, sex and postcode is possible. Overall, 92% of ONS birth records for live, singleton births born in NHS hospitals to mothers living in England were successfully linked to a HES birth record. After checking the quality of linkage in two stages, 1,456 records (<1%) were identified as potentially incorrect links. The main reasons for breaking links were different dates of birth and large amounts of data missing from key fields, making it difficult to determine if the link was correct. A higher proportion of links was broken for births in 2005 compared to births in 2006, suggesting an improvement in data quality between the two years. The addition of partially matching date of birth in the quality assurance algorithm increased sensitivity and identified more correct linkages, which would have otherwise been missed from the study cohort.

Duplicate HES records and large amounts of data missing for some variables created challenges when assessing linkage quality, highlighting the importance of these procedures before beginning statistical analysis. Finally, unlinked birth records were more likely to be unlinked if babies were of white, British ethnic origin born outside marriage, born in East England, London, North West England or the West Midlands or born in March.

Strengths and limitations

Key strengths of this study included building upon previous work conducted into the linkage of mothers' hospital records with babies' birth records, obtaining data from multiple sources, and the use of personal identifiers, which allowed us to evaluate the linkage more easily. The need to access personal identifiers also increased the problems involved in accessing the data, notably in terms of the length of approvals processes. Other limitations include the restriction of the assessment of linkage to live, singleton births in 2005 and 2006. We were unable to assess how well the algorithms designed for this study would perform with multiple births. These tend to have more complex data, although similar algorithms were successfully used for the quality of assurance of the linkage of multiple births to mothers' delivery records[6]. In addition, the algorithms used in this study were deterministic, rather than probabilistic. The latter can be more effective when dealing with complex records, such as those with a lot of missing data or coding errors[16,17].

Interpretation of findings

Approximately 8% of ONS birth records did not link to a HES birth record. One of the key reasons for unlinked records appeared to be missing data in key linking fields. Quality and completeness is always a concern when using routinely collected datasets and the large sample sizes they provide are always traded off with these limitations. Data quality was somewhat poorer in HES birth records than in ONS birth records and this is a commonly cited issue when working with HES data.[18,19] In studies using HES birth data in later years, the data quality improves with time. In 2009/10 the baby tail is far more complete, with only 18% and 14% of births with missing gestational age and birthweight, respectively.[18] Therefore, researchers who wish to analyse the linked data for the remaining years (2007-2014) should find a higher linkage rate with better quality linkage.

For peer review only - http://bmjopen.bmj.com/site/about/guidelines.xhtml

However, in previous work with mother's HES delivery records, the linkage rate plateaued at around 98% between 2010 and 2014 for singleton births, whereas the linkage rate in multiples began to decrease from 2010[7].

Compared to the full linked dataset, records of babies born in East England, North West England, London or the West Midlands were more likely to be unlinked. It is likely that these variations are due to differences between hospital trusts in the ways in which definitions or protocols are used or because errors occur during the transfer of data from one organisation to another or in the overall quality of Maternity HES. It is also possible that the regional reporting differences account for the higher proportion of white babies and births outside marriage in the unlinked dataset. Babies born in March were also more likely to have unlinked records and this was also the case with the mothers' HES delivery records[7]. HES uses financial years (1st April – 31st March) for report, so, differences in reporting standards prior to the financial year-end may account for this.

We found a small proportion of records that were potentially incorrect linkages, suggesting that linkage performed by 'Trusted Third Parties', such as NHS Digital, may not be 100% accurate. Therefore, it is essential that researchers understand the quality of linkage undertaken by a Trusted Third Party prior to performing any statistical analysis. Whilst the responsibility should fall to the Trusted Third Party to conduct quality assessment of their linkage methods and make them publicly available to researchers, this does not happen in practice.[20] The Digital Economy Act 2017 is designed to enable research for public benefit through the sharing of data, but the legislation is limited to the sharing of data that has been de-identified. This means reliance on the Trusted Third Party model in research is likely to increase over time. It is still unclear how this effects health and social care data, especially as they are not covered by the Digital Economy Act.[4] If researchers do not have access to personal identifiers, quality assurance of the linkage will become more challenging.

The findings in this paper will offer some insight into the quality of linkage between ONS birth and HES birth records and be of use to other researchers using the same linkage. As the quality and completeness of HES improved over time, it is likely that the quality of linkage will also have improved as a consequence, but the signs that the improvement in quality may not have been sustained is worrying.

Recommendations for future practice and research

The findings from this study have demonstrated a need for Trusted Third Parties to evaluate linkage methods and publish findings for researchers to use prior to beginning statistical analysis. In cases where this isn't possible, a number of studies have shown that datasets can be linked using de-identified datasets and produce generalisable cohorts, therefore, future work could explore ways to quality assure linkage without using personal identifiers. This will increase the likelihood of easier data access, potentially reducing the time required to complete the linkage and cleaning of routinely collected datasets. Finally, we did not assess whether the quality of linkage for multiples is comparable with live, singleton, NHS hospital births although quality assurance of the linkage of multiple births to mothers' delivery records suggested that it was better than had been initially assumed.[6].

For peer review only - http://bmjopen.bmj.com/site/about/guidelines.xhtml

Conclusions

By linking together administrative datasets, such as birth registration, birth notification and hospital admission data, it is possible to create more complete datasets about births which can then be linked to other administrative datasets to look at longer term outcomes.[6] If data are missing or there are coding errors and inconsistences, the resulting datasets can often be of poor quality. It is therefore essential that the quality of linkage is assessed prior to analysis. The work presented in this study provides a guide to steps taken to quality assure the linkage of births in England and may be of use to other researchers working with similar datasets. The findings will be particularly useful for researchers working with the same dataset but without access to personal identifiers.

For peer review only

Funding

This work was funded by the Medical Research Council (MRC): MR/M01228X/1. The funder had no input into the study design, data analysis, interpretation of results, or writing of the manuscript. VC and MQ had full access to all the data in the study and final responsibility for the decision to submit for publication.

Contributors

VC, MAQ and AJM were involved in the design of the study with input from Rod Gibson (Data management consultant) and Nirupa Dattani (Data analyst at City, University of London). RG and ND were involved in the cleaning of the ONS births dataset. VC performed the data manipulation and analysis. VC, AJM and MAQ were all involved in the interpretation of the data. VC was responsible for the initial draft of the manuscript. AJM and MAQ reviewed and contributed to drafts of the manuscript, and all authors have reviewed the final version.

Conflicts of interest

No conflicts of interest

Acknowledgements

We are grateful to the following organisations and individuals who were involved in this study: The MRC for funding this project (MR/M01228X/1); The Office for National Statistics (ONS) for providing access to data and hosting it within the Safe Research Setting (SRS); NHS Digital for linking and providing access to the data; Nirupa Dattani for providing her expertise on record linkage and data management; Rod Gibson, for his help cleaning the data; and Gillian Harper for her original work on the quality assurance of linkage of the mother's HES delivery records.

This work contains statistical data from ONS, which is Crown Copyright. The use of the ONS statistical data in this work does not imply the endorsement of the ONS in relation to the interpretation or analysis of the statistical data. This work uses research datasets, which may not exactly reproduce National Statistics aggregates.

Approvals

Ethics approval for this study was granted by the Health Research Authority Research Ethics Committee (South West – Frenchay; REC reference *Ethics* 15/SW/0294)

For peer review only - http://bmjopen.bmj.com/site/about/guidelines.xhtml

The TIGAR study used data linked as part of a previous study led by City, University of London.[6] For that study Permission to use patient-identifiable data without consent under Regulation 5 of the Health Service (Control of Patient Information) Regulations 2002 ('section 251 support') was initially granted by the Patient Information Advisory Group PIAG 2-10(g)/2005. Renewals and amendments and a second permission, CAG 9-08(b)2014, under Regulation 5 of the Health Service (Control of Patient Information) Regulations 2002 (or 'same legislation') were granted by the Secretary of State for Health and the Health Research Authority following advice from the CAG. A second permission CAG 9-08(b)2014 to use patient-identifiable data without consent under Regulation 5 of the Health Service (Control of Patient Information) Regulations 2002 and create a research database held at the ONS for analyses relating to inequalities in the outcome of pregnancy and to inform maternity service users about the outcome of midwifery, obstetric and neonatal care was granted by the Health Research Authority. For the TIGAR study, permission was granted by the Secretary of State for Health and the Health Research Authority following advice from the CAG (CAG reference 15/CAG/0196). Permission from the Health and Social Care Information Centre for the work described in this article was included in Data Sharing Agreement NIC-273840-N0N0 N. Permission for the subsequent analyses in the TIGAR study were included in Data Sharing Agreement.

Data Sharing Statement

The author does not have permission to supply data or identifiable information to third parties, including other researchers, but the research team of which she is a member has permission under Section 251 of the Health Service (Control of Patient Information) Regulations 2002 to analyse patient identifiable data for England and Wales without consent and create a research database which could be accessed by other researchers using the SRS at the Office for National Statistics subject to permission from the Confidentiality Advisory Group of the Health Research Authority to access patient identifiable data without consent

The authors do not have permission to supply data or identifiable information to third parties, including other researchers, but the team at City, University of London has permission under Regulation 5 of the Health Service (Control of Patient Information) Regulations 2002 to analyse patient-identifiable data for England and Wales without consent and create a research database that could be accessed by other researchers using the SRS at the ONS. The TIGAR team has permission under Regulation 5 of the Health Service (Control of Patient

For peer review only - http://bmjopen.bmj.com/site/about/guidelines.xhtml

Information) Regulations 2002 to analyse these. Anyone wishing to access the linked datasets for research purposes should apply via the CAG to the Health Research Authority to access patient-identifiable data without consent and then to the ONS and NHS Digital. In the first instance, enquiries about access to the data should be addressed to Alison Macfarlane.

For peer review only

References

1. Jorm, L. Routinely collected data as a strategic resource for research: priorities for methods and workforce. *Public Heal. Res. Pract.* **25**, 1–5 (2015).
2. Künn, S. The challenges of linking survey and administrative data. *IZA World Labor* (2015). doi:10.15185/izawol.214
3. House of Commons. *Digital Economy Act*. *The National Archives* (2017).
4. Bell, J. *et al.* Lawful disclosure of administrative data for research purposes in the UK. *J. Data Prot. Priv.* **2**, 264–277 (2019).
5. Dattani, N. & Macfarlane, A. Linkage of Maternity Hospital Episode Statistics data to birth registration and notification records for births in England 2005-2014: methods. A population-based birth cohort study. *BMJ Open* **8**, 1–7 (2018).
6. Macfarlane, A. *et al.* Births and their outcomes by time, day and year: a retrospective birth cohort data linkage study. *Heal. Serv. Deliv. Res.* **7**, (2019).
7. Harper, G. Linkage of Maternity Hospital Episode Statistics data to birth registration and notification records for births in England 2005-2014: Quality assurance of linkage of routine data for singleton and multiple births. *BMJ Open* **8**, (2018).
8. ONS. *Birth statistics: Review of the Registrar General on births and patterns of family building in England and Wales, 2006*. (2007).
9. StataCorp. Stata Statistical Software: Release 14. (2015).

For peer review only - http://bmjopen.bmj.com/site/about/guidelines.xhtml

10. Hilder, L., Moser, K., Dattani, N. & Macfarlane, A. Pilot linkage of NHS Numbers for Babies data with birth registrations. *Heal. Stat. Q.* 25–33 (2007).

11. Herbert, A., Wijlaars, L., Zylbersztejn, A., Cromwell, D. & Hardelid, P. Data Resource Profile: Hospital Episode Statistics Admitted Patient Care (HES APC). *Int. J. Epidemiol.* **46**, 1093-1093i (2017).

12. HSCIC. *Methodology for creation of the HES Patient ID (HESID)*. (2014).

13. NHS DIGITAL. HES Data Dictionary - Admitted Patient. *HSCIC* (2018). Available at: file:///Users/victoriacoathup/Downloads/DD-APC-V11.pdf. (Accessed: 6th June 2018)

14. Herbert, A., Wijlaars, L., Zylbersztejn, A., Cromwell, D. & Hardelid, P. Data Resource Profile: Hospital Episode Statistics Admitted Patient Care (HES APC). *Int. J. Epidemiol.* **46**, 1093-1093i (2017).

15. Hagger-Johnson, G. *et al.* Identifying possible false matches in anonymized hospital administrative data without patient identifiers. *Health Serv. Res.* **50**, 1162–1178 (2015).

16. Paixão, E. S. *et al.* Evaluation of record linkage of two large administrative databases in a middle income country: Stillbirths and notifications of dengue during pregnancy in Brazil. *BMC Med. Inform. Decis. Mak.* **17**, 1–9 (2017).

17. Harron, K., Gilbert, R., Cromwell, D. & Van Der Meulen, J. Linking data for mothers and babies in de-identified electronic health data. *PLoS One* **11**, 1–18 (2016).

18. Murray, J. *et al.* Quality of routine hospital birth records and the feasibility of their use for creating birth cohorts. *J. Public Heal. (United Kingdom)* **35**, 298–307 (2013).

19. Ghosh, R. E. *et al.* Routinely collected English birth data sets: Comparisons and recommendations for reproductive epidemiology. *Arch. Dis. Child. Fetal Neonatal Ed.* **101**, F451–F457 (2016).

20. Harper, G. Linkage of Maternity Hospital Episode Statistics data to birth registration and notification records for births in England 2005-2014: Quality assurance of linkage of routine data for singleton and multiple births. *BMJ Open* **8**, (2018).

Figure Legends

Figure 1. Flowchart of study population

Figure S1. Overview of data sources

For peer review only

For peer review only

For peer review only - http://bmjopen.bmj.com/site/about/guidelines.xhtml

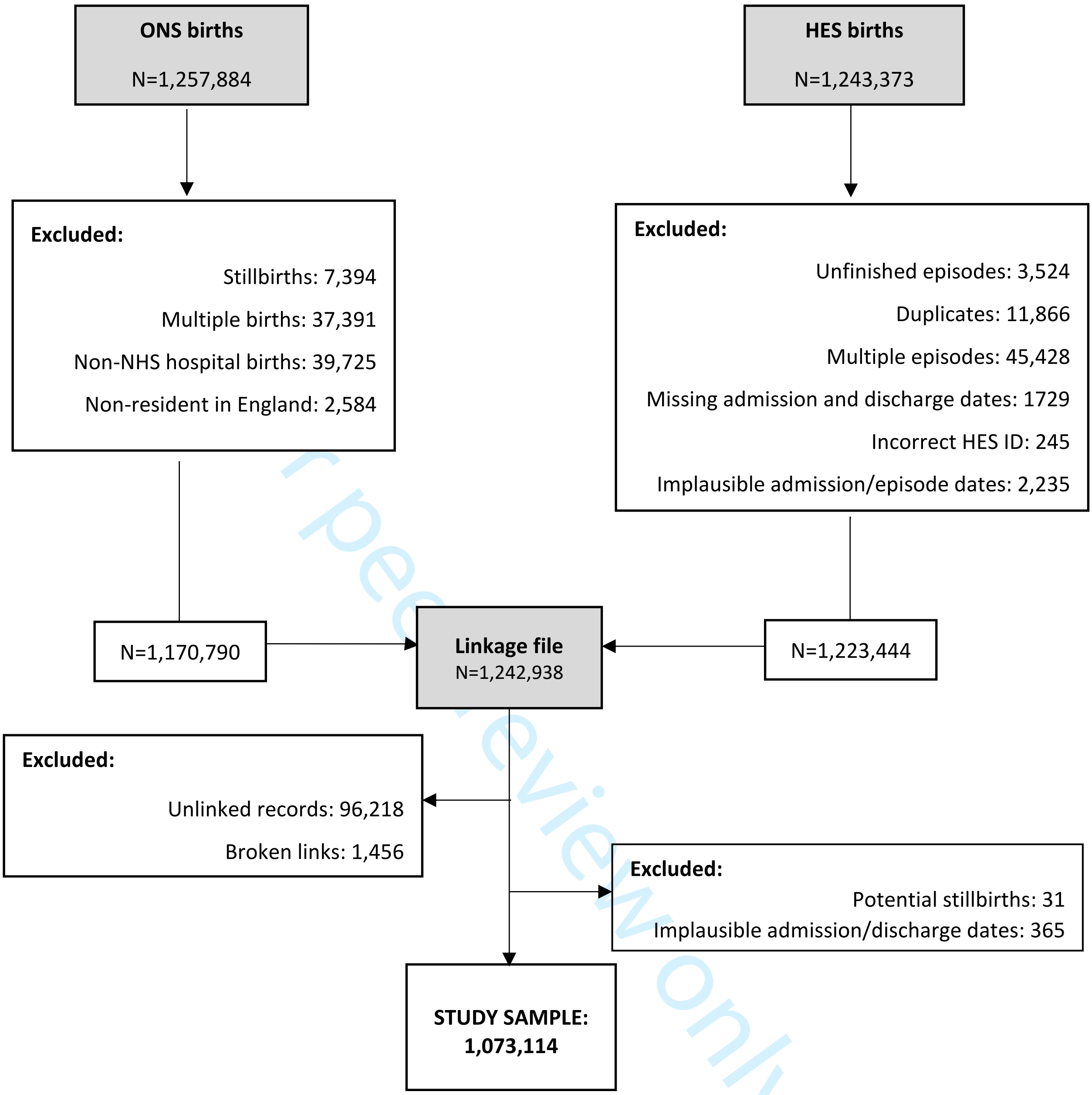

For peer review only - http://bmjopen.bmj.com/site/about/guidelines.xhtml

Supplementary information

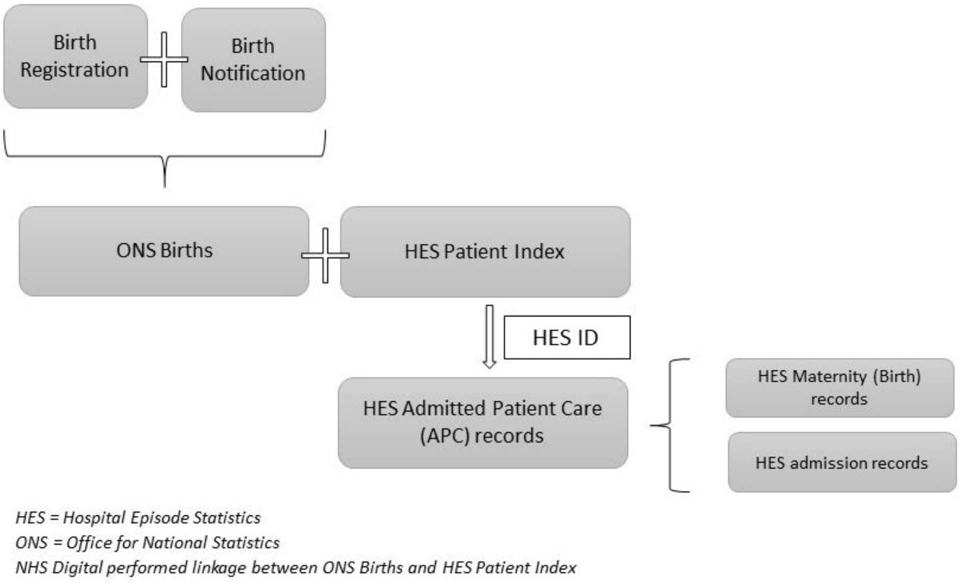

Figure S1. Overview of data sources

List of variables within the 'baby tail':

1. Anaesthetic given during labour or delivery (delpren)
2. Anaesthetic given post-labour or delivery (delposn)
3. Antenatal days of stay (antedur) (derived from other HES fields)
4. Baby's age in days (neodur) (derived from other HES fields)
5. Birth order (birorder)
6. Birth weight (birweit)
7. Delivery place change reason (delchang)
8. Delivery method (delmeth)
9. Delivery place (actual) (delplace)
10. Delivery place (intended) (delinten)
11. First antenatal assessment date (anasdate)
12. Gestation in weeks at first antenatal assessment (anagest)
13. Length of gestation (gestat)
14. Birth status (birstat)
15. Labour/delivery onset method (delonset)
16. Neonatel level of care (neocare)
17. Number of babies (numbaby)
18. Resuscitation method (biresus)
19. Status of person conducting delivery (delstat)

For peer review only - http://bmjopen.bmj.com/site/about/guidelines.xhtml

Definitions of Episode, Spell and Continuous Inpatient Stay (CIP)

HES inpatient admissions are structured as 'episodes' of care, with an episode defined as a period of care under one consultant or midwife. Multiple episodes are referred to as a 'spell' and represent an uninterrupted period of care within one hospital. A new spell is generated when the patient is transferred to another hospital to continue care. A continuous inpatient stay (CIP) may consist of one episode or multiple episodes and spells, and ends when the patient is discharged from an NHS hospital. Hospital episodes are primarily collected for financial reimbursement, and therefore, the datasets are divided into financial years, beginning 1st April and ending 31st March. Episodes are labelled as 'finished' once the patient is discharged from hospital. However, if an episode begins in one financial year and ends during the next, two episodes will be generated – one in the financial year the episode begins and one in the financial year that the episode ends – therefore, the first episode will remain as 'unfinished'.

- Unfinished episodes identified using episode type (EPITYPE = 1)

- Transfers identified using admission method, admission source and discharge destination (ADMIMETH = 81; ADMISORC = 49/50/51/52/53/87; or DISDEST = 49/50/51/52/53/84).

For peer review only

Table S1. NHS Digital algorithm used to link ONS Births with HES birth records

Match rank	Variables				Total		2005		2006	
	NHS number	Date of birth	Sex	Postcode	% Linked	% Broken	% Linked	% Broken	% Linked	% Broken
1	E	E	E	E	*80.43*	*28.23*	*75.68*	*24.61*	*85.01*	*34.45*
2	E	E	E	-	*19.20*	*9.41*	*24.02*	*8.76*	*14.52*	*10.42*
3	E	P°	E	E	*0.02*	*1.31*	*0.01*	*1.35*	*0.02*	*1.59*
4	E	P°	E	-	*0.01*	*1.03*	*0.01*	*1.12*	*0.01*	*0.71*
5	E	-	-	E	*0.02*	*2.61*	*0.02*	*1.57*	*0.03*	*4.59*
6	M	E*	E	E^	*0.31*	*53.50*	*0.22*	*58.76*	*0.40*	*44.52*
7	M	E*	E	E	*0.01*	*1.10*	*0.01*	*1.35*	*0.01*	*0.68*
8	E	-	-	-	*0.02*	*2.82*	*0.03*	*2.47*	*0.01*	*3.13*
Number of NHS births					1,073,114	1,456	525,826	890	100	566

E=Exact match, P=Partial match, M=No match, due to missing data
°Two components of data match or DOB matches, when two components are swapped
**Date of birth not 1st January*
^Postcode not on 'ignore' list

For peer review only - http://bmjopen.bmj.com/site/about/guidelines.xhtml

Table S2. Sources of variables for quality assurance

Variable	Source	Rule
Baby's date of birth	BR & BN	Use BR unless BR = missing, then use BN
Mother's date of birth	BR & BN	Use BR unless BR = missing, then use BN
Birth weight	BR & BN	Use BR unless BR = missing, then use BN
Gestational age	BR & BN	use BN as BR reported for still births only
Sex	BR & BN	Use BR unless BR = missing, then use BN
Hospital of birth	BR & BN	Use BR unless BR = missing, then use BN

BR = Birth Registration
BN = Birth Notification (NN4B)

For peer review only

Table S3. Data preparation rules for quality assurance variables

Variable	Variable name: HES	Variable name: BR/BN	Rule	Additional checks
Baby's date of birth	DOBBABY	DOB/DOBZ	Set '01Jan1801' to missing. Make sure all dates are in format 'DDMMMYYYY' e.g. '01Jan2005'.	Check dates are not <01/01/05 or > 31/12/06
Mother's date of birth	MOTDOB	DOBM/DOBMZ	Set '01Jan1801' to missing. Make sure all dates are in format 'DDMMMYYYY' e.g. '01Jan2005'	
Postcode	HOMEADD	PCDRBZ (BN)	Transform var into format of 'XXX XXX', by removing any additional punctuation and transforming any lower case letters to upper case	
Birth weight	BIRWEIT	BTHWGTZ (BN)	Set '99', '9999', <400 and ≥7000 to missing	Check all hospitals report birth weight in g. If it looks like kg, then convert to equivalent in g.
Gestational age	GESTAT	GESTZ (BN)	Set '99' to missing	Check all hospital report gestational age in weeks, if not, convert from days or months to weeks
Sex	SEXBABY	SEXZ (BN)	Transform M to 1 and F to 2 if string variable. Set '0', '3' or '9' to missing.	
Hospital of birth	PROCODE	Location Code (derived from CESTRESS/ORGCODEZ)	Remove punctuation. Transform any lower case letters to upper case	

HES = Hospital Episode Statistics
BR = Birth Registration, BN= Birth Notification (NN4B)

For peer review only - http://bmjopen.bmj.com/site/about/guidelines.xhtml

Table S4. Percentages of data missing from each dataset for key variables used to evaluate linkage of data about live, singleton births in NHS hospitals to women living in England

	ONS Births			HES Births		
**Variable**	**Total**	**2005**	**2006**	**Total**	**2005**	**2006**
NHS number	0.1	0.1	0.1	6.9	6.5	7.4
Baby's date of birth	0.0	0.0	0.0	0.0	0.0	0.0
Mother's date of birth	0.0	0.0	0.0	42.7	42.9	42.4
Mother's postcode	0.0	0.0	0.0	60.4	76.2	45.0
Hospital location code	0.0	0.0	0.0	0.0	0.0	0.0
Birthweight	0.1	0.1	0.1	44.4	43.8	45.1
Gestational age	0.8	0.8	0.8	49.1	45.7	52.6
Sex	0.0	0.0	0.0	0.0	0.0	0.0

For peer review only

Steps taken to deal with multiple HES birth records with the same HESID:

1. EPISTAT is 1 = unfinished episode: exclude all (for details of HES variables, see *NHS Digital Data Dictionary)*
2. HESID and EPIKEY exact match = duplicate record: keep any and exclude rest
3. HESID, DOB, Sex, hospital code, EPIORDER, ADMIDATE, DISDATE, EPISTART, EPIEND, DIAG_01 – DIAG_20, OPCS_01-OPCS_24, OPDATE_01-OPDATE_24 exact match and MDOB and POSTCODE either exact match or missing match = duplicate record: keep any and exclude rest
4. Two or more key variables (date of birth, Hospital code, mother's date of birth, postcode, birthweight) differ completely = unclear which record contains correct information: exclude all
5. Remainder are considered multiple episodes part of same spell = create new record using all available information from multiple episodes: keep one record and save other episodes in separate file
6. Dataset should now contain one HES birth record per baby

For peer review only

For peer review only - http://bmjopen.bmj.com/site/about/guidelines.xhtml

For peer review only

For peer review only - http://bmjopen.bmj.com/site/about/guidelines.xhtml

The RECORD statement – checklist of items, extended from the STROBE statement, that should be reported in observational studies using routinely collected health data.

	Item No.	STROBE items	Location in manuscript where items are reported	RECORD items	Location in manuscript where items are reported
**Title and abstract**					
	1	(a) Indicate the study's design with a commonly used term in the title or the abstract (b) Provide in the abstract an informative and balanced summary of what was done and what was found	Study design is included in the title and abstract	RECORD 1.1: The type of data used should be specified in the title or abstract. When possible, the name of the databases used should be included.<br><br>RECORD 1.2: If applicable, the geographic region and timeframe within which the study took place should be reported in the title or abstract.<br><br>RECORD 1.3: If linkage between databases was conducted for the study, this should be clearly stated in the title or abstract.	Type of data included in the title. Databases included in abstract<br><br>Region in title, region and timeframe mentioned in abstract<br><br>Stated in both the title and abstract
**Introduction**					
Background rationale	2	Explain the scientific background and rationale for the investigation being reported	Introduction		
Objectives	3	State specific objectives, including any prespecified hypotheses	At the end of the introduction		
**Methods**					
Study Design	4	Present key elements of study design early in the paper	Design and study population mentioned on page 3 at beginning of methods		

For peer review only

Setting	5	Describe the setting, locations, and relevant dates, including periods of recruitment, exposure, follow-up, and data collection	Described in study population section in methods		
Participants	6	*(a) Cohort study* - Give the eligibility criteria, and the sources and methods of selection of participants. Describe methods of follow-up<br>*Case-control study* - Give the eligibility criteria, and the sources and methods of case ascertainment and control selection. Give the rationale for the choice of cases and controls<br>*Cross-sectional study* - Give the eligibility criteria, and the sources and methods of selection of participants<br><br>*(b) Cohort study* - For matched studies, give matching criteria and number of exposed and unexposed<br>*Case-control study* - For matched studies, give matching criteria and the number of controls per case	Study population section within methods<br><br>N/A	RECORD 6.1: The methods of study population selection (such as codes or algorithms used to identify subjects) should be listed in detail. If this is not possible, an explanation should be provided.<br><br>RECORD 6.2: Any validation studies of the codes or algorithms used to select the population should be referenced. If validation was conducted for this study and not published elsewhere, detailed methods and results should be provided.<br><br>RECORD 6.3: If the study involved linkage of databases, consider use of a flow diagram or other graphical display to demonstrate the data linkage process, including the number of individuals with linked data at each stage.	Methods and supplementary information<br><br>N/A<br><br>Figure 1
Variables	7	Clearly define all outcomes, exposures, predictors, potential confounders, and effect modifiers. Give diagnostic criteria, if applicable.	N/A	RECORD 7.1: A complete list of codes and algorithms used to classify exposures, outcomes, confounders, and effect modifiers should be provided. If these cannot be reported, an explanation should be provided.	Supplementary information and appropriate references to sources of codes
Data sources/ measurement	8	For each variable of interest, give sources of data and details of methods of assessment (measurement).	Methods and supplementary information – but only key variables		

For peer review only

		Describe comparability of assessment methods if there is more than one group	with more than one source		
Bias	9	Describe any efforts to address potential sources of bias	Methods and supplementary information		
Study size	10	Explain how the study size was arrived at	Figure 1		
Quantitative variables	11	Explain how quantitative variables were handled in the analyses. If applicable, describe which groupings were chosen, and why	Outcomes, exposures and statistical analysis sections of methods		
Statistical methods	12	(a) Describe all statistical methods, including those used to control for confounding<br>(b) Describe any methods used to examine subgroups and interactions<br>(c) Explain how missing data were addressed<br>(d) *Cohort study* - If applicable, explain how loss to follow-up was addressed<br>*Case-control study* - If applicable, explain how matching of cases and controls was addressed<br>*Cross-sectional study* - If applicable, describe analytical methods taking account of sampling strategy<br>(e) Describe any sensitivity analyses	Assessment of linkage bias section in methods		
Data access and cleaning methods		..		RECORD 12.1: Authors should describe the extent to which the investigators had access to the database	Author contributions and Supplementary information

For peer review only

				population used to create the study population.<br><br>RECORD 12.2: Authors should provide information on the data cleaning methods used in the study.	
Linkage		..		RECORD 12.3: State whether the study included person-level, institutional-level, or other data linkage across two or more databases. The methods of linkage and methods of linkage quality evaluation should be provided.	Methods
**Results**					
Participants	13	(a) Report the numbers of individuals at each stage of the study (*e.g.*, numbers potentially eligible, examined for eligibility, confirmed eligible, included in the study, completing follow-up, and analysed)<br>(b) Give reasons for non-participation at each stage.<br>(c) Consider use of a flow diagram	Figure 1	RECORD 13.1: Describe in detail the selection of the persons included in the study (*i.e.,* study population selection) including filtering based on data quality, data availability and linkage. The selection of included persons can be described in the text and/or by means of the study flow diagram.	Figure 1
Descriptive data	14	(a) Give characteristics of study participants (*e.g.*, demographic, clinical, social) and information on exposures and potential confounders<br>(b) Indicate the number of participants with missing data for each variable of interest<br>(c) *Cohort study* - summarise follow-up time (*e.g.*, average and total amount)	Table 4 in results section		

For peer review only

Outcome data	15	*Cohort study* - Report numbers of outcome events or summary measures over time<br>*Case-control study* - Report numbers in each exposure category, or summary measures of exposure<br>*Cross-sectional study* - Report numbers of outcome events or summary measures	Table 4 in results section		
Main results	16	(a) Give unadjusted estimates and, if applicable, confounder-adjusted estimates and their precision (e.g., 95% confidence interval). Make clear which confounders were adjusted for and why they were included<br>(b) Report category boundaries when continuous variables were categorized<br>(c) If relevant, consider translating estimates of relative risk into absolute risk for a meaningful time period	N/A		
Other analyses	17	Report other analyses done—e.g., analyses of subgroups and interactions, and sensitivity analyses	N/A		
**Discussion**					
Key results	18	Summarise key results with reference to study objectives	First paragraph of discussion		
Limitations	19	Discuss limitations of the study, taking into account sources of potential bias or imprecision. Discuss both direction and magnitude of any potential bias	Strengths and limitations section of discussion	RECORD 19.1: Discuss the implications of using data that were not created or collected to answer the specific research question(s). Include discussion of misclassification bias, unmeasured confounding, missing data, and changing eligibility over	Strengths and limitations section of discussion

For peer review only

				time, as they pertain to the study being reported.	
Interpretation	20	Give a cautious overall interpretation of results considering objectives, limitations, multiplicity of analyses, results from similar studies, and other relevant evidence	Interpretation of findings section within the discussion		
Generalisability	21	Discuss the generalisability (external validity) of the study results	Interpretation of findings section within the discussion		
**Other Information**					
Funding	22	Give the source of funding and the role of the funders for the present study and, if applicable, for the original study on which the present article is based	Funding included at end of manuscript		
Accessibility of protocol, raw data, and programming code		..		RECORD 22.1: Authors should provide information on how to access any supplemental information such as the study protocol, raw data, or programming code.	Supplementary information

*Reference: Benchimol EI, Smeeth L, Guttmann A, Harron K, Moher D, Petersen I, Sørensen HT, von Elm E, Langan SM, the RECORD Working Committee. The REporting of studies Conducted using Observational Routinely-collected health Data (RECORD) Statement. *PLoS Medicine* 2015; in press.

*Checklist is protected under Creative Commons Attribution (CC BY) license.

For peer review only

BMJ Open

Linkage of Maternity Hospital Episode Statistics birth records to birth registration and notification records for births in England 2005-2006: Quality assurance of linkage

Journal:	*BMJ Open*
Manuscript ID	bmjopen-2020-037885.R2
Article Type:	Original research
Date Submitted by the Author:	10-Aug-2020
Complete List of Authors:	Coathup, Victoria; University of Oxford, Nuffield Department of Population Health<br>Macfarlane, Alison; City University, Dept of Midwifery<br>Quigley, Maria; Oxford University, National Perinatal Epidemiology Unit
**Primary Subject Heading**:	Health informatics
Secondary Subject Heading:	Epidemiology, Health services research, Public health, Research methods
Keywords:	PUBLIC HEALTH, Protocols & guidelines < HEALTH SERVICES ADMINISTRATION & MANAGEMENT, Clinical governance < HEALTH SERVICES ADMINISTRATION & MANAGEMENT

SCHOLARONE™
Manuscripts

I, the Submitting Author has the right to grant and does grant on behalf of all authors of the Work (as defined in the below author licence), an exclusive licence and/or a non-exclusive licence for contributions from authors who are: i) UK Crown employees; ii) where BMJ has agreed a CC-BY licence shall apply, and/or iii) in accordance with the terms applicable for US Federal Government officers or employees acting as part of their official duties; on a worldwide, perpetual, irrevocable, royalty-free basis to BMJ Publishing Group Ltd ("BMJ") its licensees and where the relevant Journal is co-owned by BMJ to the co-owners of the Journal, to publish the Work in this journal and any other BMJ products and to exploit all rights, as set out in our licence.

The Submitting Author accepts and understands that any supply made under these terms is made by BMJ to the Submitting Author unless you are acting as an employee on behalf of your employer or a postgraduate student of an affiliated institution which is paying any applicable article publishing charge ("APC") for Open Access articles. Where the Submitting Author wishes to make the Work available on an Open Access basis (and intends to pay the relevant APC), the terms of reuse of such Open Access shall be governed by a Creative Commons licence – details of these licences and which Creative Commons licence will apply to this Work are set out in our licence referred to above.

Other than as permitted in any relevant BMJ Author's Self Archiving Policies, I confirm this Work has not been accepted for publication elsewhere, is not being considered for publication elsewhere and does not duplicate material already published. I confirm all authors consent to publication of this Work and authorise the granting of this licence.

For peer review only

Linkage of Maternity Hospital Episode Statistics birth records to birth registration and notification records for births in England 2005-2006: Quality assurance of linkage

Dr Victoria Coathup[1], Professor Alison Macfarlane[2], Professor Maria Quigley[1]

[1]National Perinatal Epidemiology Unit (NPEU), University of Oxford, Oxford, UK

[2]Centre for Maternal and Child Health Research, School of Health Sciences, City, University of London, UK

Corresponding author

Victoria Coathup

National Perinatal Epidemiology Unit (NPEU)

Nuffield Department of Population Health

Old Road Campus

Oxford, OX3 7LF

victoria.coathup@npeu.ox.ac.uk

Abstract

Objectives: The objectives of this study were to describe the methods used to assess the quality of linkage between records of babies' birth registration and hospital birth records, and to evaluate the potential bias that may be introduced because of these methods.

Design/Setting: Data from the civil registration and the notification of births previously linked by the Office for National Statistics (ONS) had been further linked to birth records from the Hospital Episode Statistics (HES) for babies born in England. We developed a deterministic, six stage algorithm to assess the quality of this linkage.

Participants: All 1,170,790 live, singleton births, occurring in NHS hospitals in England between 1st January 2005 and 31st December 2006.

Primary outcome measure: The primary outcome was the number of successful links between ONS birth records and HES birth records. Rates of successful linkage were calculated for the cohort and the characteristics associated with unsuccessful linkage were identified.

Results: Approximately 92% (1,074,572) of the birth registration records were successfully linked with a HES birth record. Data quality and completeness were somewhat poorer in HES birth records compared to linked birth registration and birth notification records. The quality assurance algorithms identified 1,456 incorrect linkages (<1%). Compared to the linked dataset, birth records were more likely to be unlinked if babies were of white ethnic origin; born to unmarried mothers; born in East England, London, North West England or the West Midlands; or born in March.

Conclusions: It is possible to link administrative datasets to create large cohorts allowing researchers to explore important questions about exposures and childhood outcomes. Missing data and coding errors and inconsistencies mean it is important that the quality of linkage is assessed prior to analysis.

Keywords

Births, hospital records, data-linkage, Hospital Episode Statistics

For peer review only - http://bmjopen.bmj.com/site/about/guidelines.xhtml

Strengths and limitations of this study

1. This is the first study to evaluate the linkage of birth registration, birth notification and HES birth records in England.
2. We were able to build on existing work relating to quality assurance of birth registration, birth notification and HES delivery records.
3. Access to personal identifiers meant we were able to evaluate the quality of linkage, identify poor quality matches and assess the level of bias introduced by linking these datasets.
4. Accessing personal identifiers also led to delays because of the length of approval processes and the need to travel to access these data in designated secure settings. However, the Office for National Statistics is developing a remote access system for the secure setting, so future research studies may not be affected by the need to travel .
5. We examined only singleton births in NHS hospitals so we did not assess whether the linkage of multiple births is of comparable quality. However, previous work linking birth registration records with mothers' delivery records in multiples suggests that the linkage rate is similar to that for singletons.

For peer review only - http://bmjopen.bmj.com/site/about/guidelines.xhtml

Introduction

The use of routinely collected datasets within research has increased rapidly over the past decade as an alternative to conducting large, observational studies, which can be extremely costly and often suffer from poor recruitment and retention rates[1]. Whilst there are many advantages to using linked administrative data for medical research, they also present challenges. One is the quality of data recorded and, in consequence, the quality of data linkage, as this is highly reliant upon the availability and accuracy of personal identifiers[2] and other supporting information.

The Digital Economy Act 2017[3] was introduced with the aim of facilitating data sharing for research purposes, but only if the data have been 'de-identified' and the research is deemed to be in the public interest[4]. Without access to personal identifiers, successful linkage between datasets becomes more challenging. Therefore, the 'Trusted Third Party' model, whereby the full identifiers are transferred to an organisation, which will link them with its own data and return the linked data to the data controller, is now the preferred method of linkage in most research projects. Whilst Trusted Third Parties typically publish their linkage algorithms, they usually do not publish results of quality assurance of their methods. Therefore, it is essential that researchers assess the quality of linkage and validity of data prior to conducting statistical analyses.

The work described in this paper was conducted as part of the (Tracking the Impact of Gestational Age on Health, Educational and Economic outcomes: a Longitudinal Records Linkage Study) TIGAR study, which is a population-based, record-linkage study of births and hospital admission data in England. The study aimed to estimate the association between gestational age at birth and rates of hospital admission throughout childhood. This used previously linked data from two sources.[5] The first of these was civil registration of births, a legal process in which parents register the birth and provide mainly demographic information to a specially trained local registrar of births, deaths and marriages who records the information, issues birth certificates and forwards the data to national systems. The second is the data recorded at the notification of the birth within 36 hours to the National Health Service (NHS) by the attending midwife or other birth attendant, and the baby's NHS Number, a unique identifier, is allocated. These combined ONS birth records were linked with birth records from Hospital Episode Statistics (HES), the national hospital discharge system for England. This was done by the data owner, the Health and Social Care Information Centre, now known as NHS Digital, as part of an earlier, larger study by City, University of London[6].

Two HES records are generated when a baby is born in England, one for the mother and one for each baby. Each consists of an Admitted Patient Care record common to all hospital in-patient stays plus a 'tail' with information about the birth (Figure S1). The mother's HES delivery record contains Admitted Patient Care

For peer review only - http://bmjopen.bmj.com/site/about/guidelines.xhtml

information relating to the mother's delivery and a 'maternity' tail'. The baby's HES birth record contains the baby's Admitted Patient Care record and a 'baby tail' containing details of the birth and overlapping extensively with the 'maternity tail'. Maternity HES data are downloaded from hospitals' administrative systems. As these do not all come from the same supplier, there are some differences in the ways in which data are entered and there are differences between systems and hospitals in the extent to which data items are missing.

The team at City, University of London has already evaluated the quality of linkage between birth registration, birth notification and the mother's HES delivery records for births from 2005 to 2014.The authors reported a linkage rate of 95% and uncovered some linkage errors[7]. Therefore, the main objectives of the current study were to assess the quality of linkage between baby's birth registration and notification records and the baby's HES birth records, and to evaluate the potential bias introduced to the study cohort by the linkage. This has relevance for analyses of similar linked administrative datasets.

Methods

All live, singleton babies born in NHS hospitals between 1st January 2005 and 31st December 2006 to a mother living in England were eligible for inclusion in this study cohort. The analysis was restricted to births in NHS hospitals, but they accounted for 96.6 per cent of women giving birth in 2006. Home births accounted for 2.7 per cent of deliveries in 2006, but although most received NHS care, many were not recorded on hospital systems and so not included in HES. There were extremely few HES records for the 0.5 per cent of deliveries in non-NHS hospitals and the 0.2 per cent delivering elsewhere.[8]

All analyses were conducted using STATA 14[9] within the Office for National Statistics (ONS)' Secure Research Service (SRS). An overview of the procedures involved in the quality assurance process for this study is presented in **Figure 1**.

Figure 1. Flow chart of study population

Data sources

Two datasets containing data about births in England from 1st January 2005 to 31st December 2006 were linked (**Figure S1**). They and the linkage file were saved as three separate STATA datasets. The datasets included: (A) ONS Births; (B) Hospital Episode Statistics (HES) birth records; and (C) the Linkage file containing unique ONS birth identifiers (ONS_id) and corresponding unique HES identifiers (HESID). These files are described in the supplementary information (**Figure S1**).

A. ONS births

For peer review only - http://bmjopen.bmj.com/site/about/guidelines.xhtml

The master dataset comprises data from two sources: birth registration and birth notification These two datasets have been routinely linked by the ONS since 2006 and the combined dataset is referred to as 'ONS births' throughout this paper. ONS births contains personal identifiers, socio-demographic characteristics and birth characteristics.[10]

B. Hospital Episode Statistics (HES Birth records)

HES is a large database containing records of all episodes of care and births in NHS hospitals in England since 1989. The records used here are from the HES Admitted Patient Care (APC) dataset, which contains records of all inpatient admissions, including birth and delivery records, to NHS hospitals across England. A full description of the database can be found elsewhere.[11] Briefly, HES inpatient admissions are structured as 'episodes' of care, with an episode defined as a period of care under one consultant or midwife. Each episode contains details relating to the individual, care provider and care received (including diagnosis and procedural codes). If a patient receives care in more than one department, this generates multiple episodes, referred to as a 'spell' and represents an uninterrupted period of care within one hospital. A new spell is generated when the patient is transferred to a different hospital to continue care. A continuous inpatient stay (CIP) may consist of one or more episodes and spells, and ends when the patient is discharged from an NHS hospital. Data about hospital episodes are primarily collected for financial reimbursement, and therefore, the datasets are divided into financial years, beginning 1st April and ending 31st March. Episodes are labelled as 'finished' once the patient is discharged from hospital. However, if an episode begins in one financial year and ends during the next, two episodes will be generated; one in the financial year the episode begins and one in the financial year that the episode ends. In this case,, the first episode will be defined as 'unfinished'.

Everyone for whom records are stored in HES is assigned a unique identifier, called the HESID.[12] This is generated using a combination of NHS Number, local patient identifier, postcode, sex and date of birth to enable data users to uniquely track patients throughout the NHS. Descriptions of the variables available are in NHS Digital's HES Data Dictionary[13].

When a baby is born, the general inpatient episode becomes part of the Maternity HES dataset and 19 additional variables relating to the delivery or birth are appended. For each birth, two Maternity HES records are generated. Firstly, a HES delivery record, which includes the general inpatient record for the mother and 19 additional variables, referred to as the 'maternity tail'. Secondly, a HES birth record, which includes the general inpatient record for the baby, along with 19 additional variables, referred to as the 'baby tail'. The maternity and baby tails contain similar information relating to the delivery and birth; however, this study evaluated the

For peer review only - http://bmjopen.bmj.com/site/about/guidelines.xhtml

linkage of HES birth records only. These additional data items in the 'baby tail' include variables such as gestational age at birth and neonatal level of care. For a full list, please see section A in the supplementary information.

C. Linkage file

The linkage file contained the unique identifiers from each dataset as linked by NHS Digital. The dataset contained unique identifiers from ONS births that had been successfully linked with the corresponding unique identifier from HES births and those which had not. It also contained unique HES birth identifiers that were not linked to a unique ONS birth identifier.

Linkage of the Baby cohort and Maternity HES

Linkage between ONS birth records and HES birth records is a two-step process. Firstly, ONS birth records were linked to the HES patient index (an index of personal details relating to all individuals with access to NHS hospitals) by NHS Digital using a deterministic algorithm in order to assign the HESID; and secondly, records were linked to the corresponding HES birth records using the HESID and other identifiers.[5] Here, each linked record was assigned a match rank score, indicating the stage in the algorithm at which the records had been matched (1 being highest quality). Steps of the algorithm are summarised in **Table S1**.

Data preparation for quality assurance checks

Full details of available variables, data cleaning procedures and preparation steps for the included datasets have are described elsewhere.[6] Many key variables were available from both birth registration and birth notification. The preferred sources are summarised in the supplementary information (**Table S2)**. A number of additional steps were taken to ensure that key variables in HES births and ONS births were in consistent formats, ready for comparison during the quality assurance procedure (**Table S3**). Further checks were conducted on HES birth records and those with: (1) discharge date occurring before admission; (2) discharge occurring before baby's date of birth; or (3) admission dates occurring before 1st January 2005 or after 31st December 2006 were excluded.

Multiple episodes with the same HESID (i.e. duplicate HES birth records)

Birth episodes with the same HESID can occur due to a number of reasons: (1) unfinished episodes, such as a baby being born on the 30th March, but then discharged on the 1st April[14]; (2) administrative errors[7]; (3) a birth spell containing multiple episodes, such as a baby being transferred to a different hospital or consultant; and (4) HESID is incorrectly assigned to more than one baby[15]. The steps which were taken have been summarised in

For peer review only - http://bmjopen.bmj.com/site/about/guidelines.xhtml

the supplementary information. In addition to this, episode start dates that occurred more than two days after the previous episode ended and did not include a transfer code (see supplementary information) were considered a new admission and were saved separately.

Quality assurance of the baby cohort and Maternity HES linkage

After merging the three datasets, we identified cases where two different ONS birth records had been linked to the same HES birth record and key characteristics were compared to identify the correct link. This was done by comparing the following variables which were available in both data sources: baby's date of birth, gestational age, birth weight, sex, mother's date of birth and postcode. The record with the highest number of matching variables was identified as the correct link. When records matched on the same number of variables, the record that matched exactly on birthweight was identified as the correct match. If birthweight was missing or did not match with either record, then the record with the highest match rank score from the original linkage (Table S1) was identified as the correct link. The remaining records were then compared manually, but in cases where records had a high proportion of missing data or the same number of matching variables and the same match rank score, it was not possible to identify the correct link and therefore all were excluded.

The deterministic algorithm developed for evaluating the record linkage is summarised in **Table 1**. The quality assurance (QA) algorithm was adapted from the previous study which assessed the quality of linkage between ONS birth records and the mother's HES delivery records[7], and was based on location of birth, date of birth, sex, birthweight and gestational age. The location of birth was defined as the hospital trust running the hospital that the baby was born in. A hospital trust in the UK is an organisational unit within the NHS and usually refers to a group of hospitals that are in close proximity to each other. This was used instead of the hospital of birth to account for potential transfers between hospitals within the birth admission. The hospital trust variable was developed as part of a previous study[6].To account for differences in rounding between hospitals, a birthweight of + or – 100g was considered a match.

For peer review only - http://bmjopen.bmj.com/site/about/guidelines.xhtml

Table 1. Quality assurance algorithm to assess linkage of ONS birth records to HES birth records

Step	Variable												
	**Hospital Trust code**		**Date of birth**		**Sex**		**Birthweight**		**Gestational age**		**Mother's date of birth**		**Postcode**
1	E	*and*	E	*and*	E	*and*	E	*and*	E		-		-
2	E	*and*	E	*and*	E	*and*	(E	*or*	E)		-		-
3	E	*and*	E	*and*	E		-		-	*and*	E/M	*and*	E/M
4	-		E	*and*	E	*and*	E/M	*and*	E/M	*and*	E/M	*and*	E/M
5	E	*and*	P	*and*	E	*and*	(E	*or*	E	*or*	E	*or*	E)
6	E	*and*	P		-		E/M	*and*	E/M	*and*	E/M	*and*	E/M

E = Exact match; M = Missing; P = Partial match (differs by up to four days, two elements of data match or dates match if day and month swapped)

For peer review only

Final checks after the quality assurance procedures were completed included: (1) Checking that the baby's date of birth in ONS births was within the admission and discharge dates from the HES birth record; (2) Baby's date of birth in ONS births did not occur before 1st January 2005 or after 31st December 2006; (3) hospital discharge date did not occur before the admission date; and (4) further checks to ensure that all stillbirths had been excluded (ICD10 diagnosis code = Z37.1).

Assessment of linkage bias

The distributions of key characteristics were compared between the linked and unlinked study samples and the eligible study sample. The chi-squared test was used to assess whether distributions differed significantly between the linked and unlinked study samples. Because the sample sizes were so large we also looked at differences in distributions of variables, for example, differences between proportions of more than 1%. In addition to this, we compared the distributions of key characteristics between the linked study sample and all live births in England in 2005 and 2006 to see what effect excluding stillbirths, multiple and non-NHS births had on the study cohort.

Patient and Public Involvement

The TIGAR study was supported by a patient, parent and public advisory group, which provided input to different aspects of the study. This group met at the start of the study and gave input into the study protocol and the lay summary of the project.

Results

There were 1,257,884 ONS birth records. After excluding multiple births and babies who were not eligible for the TIGAR study, 1,170,790 live, singleton babies born from 1st January 2005 to 31st December 2006 in NHS hospitals to women living in England remained in the study cohort. There were 1,243,373 HES birth records and the linkage file included 1,242,938 records (**Figure 1**).

Linkage of baby cohort to Maternity HES

Of the 1,170,790 eligible ONS birth records, 1,074,571 or 92% were successfully linked with a HES birth record. Of the 96,219 unlinked records, 88,471 had a HESID but no corresponding HES birth record and 7,747 had no corresponding HESID in the linkage file. The majority of records linked had a match rank score of one, which meant they exactly matched on all four variables in the NHS Digital algorithm, with fewer than 1% of records linking in stages three to six of the algorithm (Table S1). A higher proportion of links had a match rank score of one in 2006 compared to 2005, however.

Data preparation for quality assurance checks

All key variables in ONS birth records had data missing for less than 1% of births. In contrast, data were missing from substantial numbers of HES birth records (**Table S4**).

For peer review only - http://bmjopen.bmj.com/site/about/guidelines.xhtml

Multiple birth episodes with the same HESID

Of the 1,243,373 HES birth records, 73,307 (6%) had linked with more than one record with the same HESID. Of these, the most common reasons for this were multiple episodes within a hospital spell (62%), unfinished episodes (15%) and duplicate episodes due to administrative errors (11%) (**Figure 1**). There were 1,197,999 unique HES birth records remaining.

Quality assurance of the baby cohort and Maternity HES linkage

A total of 979 (0.1%) HES birth records had linked with more than one ONS birth record. The high proportion of missing data in HES birth records meant it was difficult in many cases to identify the correct match. Of these, 499 records were judged to be incorrectly linked and were broken. It was not possible to ascertain the status of 95 records, which had the same matching variables and match rank score. A lot of data items in these records were missing data and all were excluded (**Table 2)**.

For peer review only

For peer review only - http://bmjopen.bmj.com/site/about/guidelines.xhtml

Table 2. Proportions of birth records at each quality assurance stage in 2005 and 2006 for live, singleton births born in NHS hospitals to mother's living in England

	Quality assurance stage	Total	2005		2006	
		n	n	%	n	%
1	Number of Births*	1,170,790	575,568	*49.2*	595,222	*50.8*
2	Number of ONS Birth records linked to HESID	1,148,110	563,963	*49.1*	584,147	*50.9*
3	Number of ONS Birth records not linked to HESID	22,680	11,605	*51.2*	11,075	*48.8*
4	Number of ONS Birth records linked to HESID and HES birth record	1,074,571	530,586	*49.4*	543,985	*50.6*
5	Number of ONS Birth records linked to HESID but not linked to HES birth record	96,219	44,982	*46.7*	51,237	*53.3*
6	Number of ONS Birth records linked to one HES birth record before QA	1,074,571	530,586	*49.4*	543,985	*50.6*
7	Number of links broken between ONS Birth records and HES birth records after QA	1,456	890	*61.1*	566	*38.9*
8	Number of ONS Birth records linked to one HES birth record after QA	1,073,115	530,119	*49.4*	542,996	*50.6*
-	% of total ONS birth records left linked to one HES birth record after QA	91.7	-	*92.1*	-	*91.3*
9	Total number of ONS Birth records not linked to HES birth record after QA	97,675	45,810	*46.9*	51,865	*53.1*
-	% of total ONS birth records not linked to HES birth record after QA	8.3	-	*7.9*	-	*8.7*

**Birth registration linked to birth notification records of live, singleton births that occurred in NHS hospitals to women residing in England*

For peer review only

Key variables of the 1,074,571 (92%) ONS birth records, which had been successfully linked, were then compared using the algorithm in **Table 1**. The vast majority (99.5%) of correct links were found to be within the first three stages of the quality assurance algorithm (**Table 3**). The 645 records identified as correct links in stage four of the algorithm appeared to be correct matches; the hospital location code differed slightly, which may have resulted from either a transfer to a neighbouring hospital trust during the birth admission or from data entry errors. In stage five, 1,973 (0.2%) records were identified as correct links. Of these, 76% had a match rank score of one, suggesting the date of birth had been entered incorrectly in the HES birth record. The majority of these matches looked like data entry errors where the month and day had inadvertently been swapped. In stage six of the algorithm, a small number of records were identified as correct links. These records had a lot of data missing, including 64% of birthweights, 70% of gestational ages, 77% of postcodes; and 67% of mother's dates of birth. Of the 242 records with partially matching dates of births, 91% had a match rank score of one or two, suggesting good quality matches but with data entry errors in the HES birth records. Interestingly, almost 62% of records in this stage exactly matched on date of birth, but differed by sex. Most of these records also exactly matched on birthweight, gestational age, postcode and mother's date of birth, suggesting they are correct links, again with data entry errors in the HES birth record.

Table 3. Proportion of birth records identified as correct links at each stage of the quality assurance process

**Step**	**Total**		**2005**		**2006**	
	Number	%	Number	%	Number	%
**1**	385,570	*35.9*	207,106	*39.1*	178,464	*32.8*
**2**	301,066	*28.0*	134,684	*25.4*	166,382	*30.6*
**3**	383,189	*35.6*	186,203	*35.1*	196,986	*36.2*
**4**	645	*0.1*	426	*0.1*	219	*0.0*
**5**	1,973	*0.2*	983	*0.2*	990	*0.2*
**6**	671	*0.0*	379	*0.0*	292	*0.0*
**Poor quality links**	**860**	***0.1***	**548**	***0.1***	**312**	***0.1***
**All**	**1,074,571**	***100***	**530,586**	***100***	**543,985**	***100***

Overall, 860 records with incorrect links were identified and 64% of these were in births in 2005, suggesting an improvement in data and linkage quality in 2006. When exploring these broken links in relation to their match rank score, 69% had a score of six, meaning their NHS numbers were missing. The main reason for broken links was completely different dates of birth as opposed to exact or partial matching and data missing for large numbers of variables. Among these records, birthweight, gestational age, postcode and mother's date of birth was missing for approximately 92%, 91%, 85% and 91%, respectively. There were a small number of records that appeared to be incorrectly broken due to cleaning errors, for example postcodes which included an 'O' instead of a zero.

A number of interesting observations were made when reviewing the broken links: (1) Many that were broken differed by gestational age, but in most cases by just one week and the babies were full term for example 39 and

For peer review only - http://bmjopen.bmj.com/site/about/guidelines.xhtml

For peer review only

40 weeks; and (2) More than one third of broken links were for babies born in London. As the numbers of broken links were small, distributions of numbers of broken links cannot be presented, as they could be disclosive.

Assessment of linkage bias

Distributions of key variables for all live births, all eligible births, and all linked and all unlinked births are presented in **Table 4**. Compared to the linked dataset, the distribution of those in the unlinked dataset differed by baby's ethnicity, mother's age, parity, Index of Multiple Deprivation score (IMD) score, sex, registration status, region and month of birth, birth weight and gestational age ($p<0.05$). Although absolute differences were small (<1%) for most variables, there were some noticeable discrepancies, where distributions differed by more than 1%. Unlinked records were more likely occur if babies were of white ethnic origin; born outside marriage; born in East England, London, North West England or the West Midlands; or born in March. Compared to all eligible births in 2005 and 2006, the distributions of linked births were very similar and differed by less than 1% in all cases.

For peer review only - http://bmjopen.bmj.com/site/about/guidelines.xhtml

Table 4. Comparison of all live, all eligible, all inked and all unlinked datasets

Characteristics	All live births (n=1,250,490)		All eligible (n=1,170,790)		Linked (n=1,073,115)		Unlinked (n=97,675)		
	n	%	n	%	n	%	n	%	p-value[a]
**Ethnicity**									
Bangladeshi	16,730	*1.3*	16,281	*1.4*	15,356	*1.4*	925	*1.0*	p<0.000
Indian	32,241	*2.6*	31,287	*2.7*	28,970	*2.7*	2,318	*2.4*	
Pakistani	48,570	*3.9*	47,352	*4.0*	43,702	*4.1*	3,648	*3.7*	
Black African	24,461	*2.0*	23,394	*2.0*	21,504	*2.0*	1,888	*1.9*	
Black Caribbean	8,668	*0.7*	8,200	*0.7*	7,420	*0.7*	781	*0.8*	
White British	788,985	*63.0*	738,88	*63.1*	675,34	*62.9*	63,552	*65.1*	
White Other	91,311	*7.3*	86,918	*7.4*	80,611	*7.5*	6,306	*6.5*	
Other	102,048	*8.2*	97,362	*8.3*	89,043	*8.3*	8,318	*8.5*	
Missing	137,476	*11.0*	121,10	*10.3*	111,16	*10.4*	9,939	*10.2*	
**Mother's age**									
Under20	52,605	*4.2*	51,312	*4.4*	46,803	*4.4*	4,509	*4.6*	p<0.000
20-24	216,768	*17.3*	208,68	*17.8*	190,72	*17.8*	17,960	*18.4*	
25-29	306,603	*24.5*	290,13	*24.8*	266,00	*24.8*	24,130	*24.7*	
30-34	363,312	*29.1*	337,32	*28.8*	309,60	*28.9*	27,718	*28.4*	
35-39	244,952	*19.6*	223,25	*19.1*	204,99	*19.1*	18,263	*18.7*	
40+	66,250	*5.3*	60,079	*5.1*	54,987	*5.1*	5095	*5.2*	
**Nulliparous**									
No	587,834	*47.0*	562,20	*48.0*	523,05	*48.7*	39,152	*40.1*	p<0.000
Yes	570,129	*45.5*	548,25	*46.8*	505,98	*47.2*	42,279	*43.3*	
Missing	92,527	*7.4*	60,325	*5.2*	44,083	*4.1*	16,244	*16.6*	
**IMD Score (quintiles)**									
Q1	333,809	*26.7*	318,83	*27.2*	292,31	*27.2*	26,513	*27.1*	p=0.001
Q2	263,212	*21.1*	247,77	*21.2*	227,08	*21.2*	20,700	*21.2*	
Q3	221,720	*17.7*	206,64	*17.7*	189,75	*17.7*	16,891	*17.3*	
Q4	200,891	*16.1*	186,16	*15.9*	170,31	*15.9*	15,851	*16.2*	
Q5	195,382	*15.6*	181,04	*15.5*	165,76	*15.5*	15,277	*15.6*	
Missing	35,476	*2.8*	30,327	*2.6*	27,886	*2.6*	2,443	*2.5*	
**Sex**									
Male	639,617	*51.2*	599,71	*51.2*	549,23	*51.2*	50,484	*51.7*	p=0.002
Female	610,873	*48.9*	571,07	*48.8*	523,88	*48.8*	47,191	*48.3*	
**Mother UK born**									
No	274,659	*22.0*	259,17	*22.1*	237,66	*22.2*	21,504	*22.0*	p=0.413
Yes	974,065	*77.9*	909,96	*77.7*	833,94	*77.7*	76,022	*77.8*	
Missing	1,766	*0.1*	1,658	*0.1*	1,509	*0.1*	149	*0.2*	
**Registration status**									
Married	716,858	*57.3*	665,76	*56.9*	612,06	*57.0*	53,698	*55.0*	p<0.000
Sole registrant	85,500	*6.8*	81,080	*6.9*	74,158	*6.9*	6,922	*7.1*	
Partner, same address	339,641	*27.2*	320,09	*27.3*	292,33	*27.2*	27,762	*28.4*	
Partner, different	108,491	*8.7*	103,84	*8.9*	94,556	*8.8*	9,293	*9.5*	
**Region of birth**									
East Midlands	87,310	*7.0*	82,311	*7.0*	77,750	*7.3*	4,561	*4.7*	p<0.000
East of England	126,925	*10.1*	118,49	*10.1*	106,96	*10.0*	11,530	*11.8*	
London	248,341	*19.9*	228,98	*19.6*	208,22	*19.4*	20,756	*21.3*	
North East	61,007	*4.9*	57,491	*4.9*	54,569	*5.1*	2,922	*3.0*	
North West	170,504	*13.6*	159,85	*13.7*	142,85	*13.3*	17,003	*17.4*	
South Central	88,798	*7.1*	83,610	*7.1*	78,451	*7.3*	5,159	*5.3*	
South East Coast	96,606	*7.7*	90,875	*7.8*	83,956	*7.8*	6,921	*7.1*	
South West	107,376	*8.6*	101,12	*8.6*	98,438	*9.2*	2,682	*2.8*	
West Midlands	137,859	*11.0*	129,22	*11.0*	108,06	*10.1*	21,160	*21.7*	
Yorkshire/Humber	125,765	*10.1*	118,83	*10.2*	113,85	*10.6*	4,981	*5.1*	

[a] *chi-square test: linked vs. unlinked*

***All live births** = all live births within the ONS birth cohort*

***All eligible births** = all live, singleton, NHS hospital births to mothers living in England within the ONS birth cohort*

***Linked dataset** = all eligible births from ONS birth cohort that successfully linked with a HES birth record*

***Unlinked dataset** = all eligible births from ONS birth cohort that did not successfully link with a HES birth record*

For peer review only - http://bmjopen.bmj.com/site/about/guidelines.xhtml

For peer review only

Table 4 cont. Comparison of all live, all eligible, all inked and all unlinked datasets

Characteristics	All live births (n=1,250,490)		All eligible births (n=1,170,970)		Linked dataset (n=1,073,115)		Unlinked (n=97,675)		
	n	%	n	%	n	%	n	%	p-value[a]
**Month of birth**									
January	100,604	*8.1*	94,233	*8.1*	85,746	*8.0*	8,487	*8.7*	p<0.0001
February	93,456	*7.5*	87,683	*7.5*	78,333	*7.3*	9,350	*9.6*	
March	104,069	*8.3*	97,526	*8.3*	85,779	*8.0*	11,747	*12.0*	
April	99,977	*8.0*	93,633	*8.0*	86,701	*8.1*	6,933	*7.1*	
May	104,992	*8.4*	98,313	*8.4*	90,856	*8.5*	7,455	*7.6*	
June	105,124	*8.4*	98,149	*8.4*	90,082	*8.4*	8,071	*8.3*	
July	108,249	*8.7*	101,330	*8.6*	93,908	*8.8*	7,421	*7.6*	
August	109,968	*8.8*	103,229	*8.8*	95,674	*8.9*	7,555	*7.7*	
September	109,717	*8.8*	102,752	*8.8*	94,918	*8.9*	7,833	*8.0*	
October	108,485	*8.7*	101,301	*8.6*	94,421	*8.8*	6,881	*7.0*	
November	102,517	*8.2*	95,848	*8.2*	87,938	*8.2*	7,910	*8.1*	
December	103,332	*8.3*	96,793	*8.3*	88,759	*8.3*	8,032	*8.2*	
**Birth weight (g)**									
<1500	15,130	*1.2*	11,073	*1.0*	10,016	*0.9*	1,056	*1.1*	p<0.0001
1500-1999	19,282	*1.5*	13,439	*1.2*	12,454	*1.2*	985	*1.0*	
2000-2499	60,012	*4.8*	47,825	*4.1*	44,089	*4.1*	3,738	*3.8*	
2500-2999	213,073	*17.0*	195,554	*16.7*	179,236	*16.7*	16,319	*16.7*	
3000-3499	447,030	*35.8*	427,600	*36.5*	391,926	*36.5*	35,675	*36.5*	
3500-3999	357,505	*28.6*	342,606	*29.3*	314,060	*29.3*	28,540	*29.2*	
4000-4499	116,484	*9.3*	111,708	*9.5*	102,534	*9.6*	9,176	*9.4*	
4500+	20,730	*1.7*	19,940	*1.7*	18,328	*1.7*	1,611	*1.7*	
Missing	1,244	*0.1*	1,045	*1.0*	472	*0.0*	575	*0.6*	
**Gestational age**									
<28	6,071	*0.5*	4,661	*0.4*	4,061	*0.4*	600	*0.6*	p<0.0001
28	2,035	*0.2*	1,524	*0.1*	1,382	*0.1*	142	*0.2*	
29	2,333	*0.2*	1,739	*0.2*	1,602	*0.2*	137	*0.1*	
30	2,999	*0.2*	2,261	*0.2*	2,071	*0.2*	189	*0.2*	
31	3,816	*0.3*	2,791	*0.2*	2,570	*0.2*	221	*0.2*	
32	5,367	*0.4*	3,820	*0.3*	3,509	*0.3*	311	*0.3*	
33	7,520	*0.6*	5,581	*0.5*	5,193	*0.5*	388	*0.4*	
34	12,261	*1.0*	9,225	*0.8*	8,506	*0.8*	719	*0.7*	
35	18,049	*1.4*	14,170	*1.2*	13,049	*1.2*	1,122	*1.2*	
36	33,701	*2.7*	27,535	*2.4*	25,271	*2.4*	2,264	*2.3*	
37	71,643	*5.7*	62,034	*5.3*	56,917	*5.3*	5,117	*5.2*	
38	168,887	*13.5*	156,200	*13.3*	142,975	*13.3*	13,226	*13.5*	
39	272,469	*21.8*	260,443	*22.3*	238,741	*22.3*	21,707	*22.2*	
40	337,793	*27.0*	322,748	*27.6*	296,399	*27.6*	26,347	*27.0*	
41	242,264	*19.4*	234,547	*20.0*	215,217	*20.1*	19,325	*19.8*	
42	48,240	*3.9*	47,237	*4.0*	43,521	*4.1*	3,716	*3.8*	
43+	5,063	*0.4*	4,893	*0.4*	4,578	*0.4*	315	*0.3*	
Missing	9,979	*0.8*	9,381	*0.8*	7,553	*0.7*	1,829	*1.9*	

[a]chi-square test: linked vs. unlinked

***All live births** = all live births within the ONS birth cohort*

***All eligible births** = all live, singleton, NHS hospital births to mothers living in England within the ONS birth cohort*

***Linked dataset** = all eligible births from ONS birth cohort that successfully linked with a HES birth record*

***Unlinked dataset** = all eligible births from ONS birth cohort that did not successfully link with a HES birth record*

For peer review only - http://bmjopen.bmj.com/site/about/guidelines.xhtml

Discussion

Key findings

Our study has shown that linkage between ONS births and HES births based on NHS number; baby's date of birth, sex and postcode is possible. Overall, 92% of ONS birth records for live, singleton births born in NHS hospitals to mothers living in England were successfully linked to a HES birth record. After checking the quality of linkage in two stages, 1,456 records (<1%) were identified as potentially incorrect links. The main reasons for breaking links were different dates of birth and large amounts of data missing from key fields, making it difficult to determine if the link was correct. A higher proportion of links was broken for births in 2005 compared to births in 2006, suggesting an improvement in data quality between the two years. The addition of partially matching date of birth in the quality assurance algorithm increased sensitivity and identified more correct linkages, which would have otherwise been missed from the study cohort.

Duplicate HES records and large amounts of data missing for some variables created challenges when assessing linkage quality, highlighting the importance of these procedures before beginning statistical analysis. Finally, unlinked birth records were more likely to be unlinked if babies were of white, British ethnic origin born outside marriage, born in East England, London, North West England or the West Midlands or born in March.

Strengths and limitations

Key strengths of this study included building upon previous work conducted into the linkage of mothers' hospital records with babies' birth records, obtaining data from multiple sources, and the use of personal identifiers, which allowed us to evaluate the linkage more easily. The need to access personal identifiers also increased the problems involved in accessing the data, notably in terms of the length of approvals processes. Other limitations include the restriction of the assessment of linkage to live, singleton births in 2005 and 2006. We were unable to assess how well the algorithms designed for this study would perform with multiple births. These tend to have more complex data, although similar algorithms were successfully used for the quality of assurance of the linkage of multiple births to mothers' delivery records[6]. In addition, the algorithms used in this study were deterministic, rather than probabilistic. The latter can be more effective when dealing with complex records, such as those with a lot of missing data or coding errors[16,17].

Interpretation of findings

Approximately 8% of ONS birth records did not link to a HES birth record. One of the key reasons for unlinked records appeared to be missing data in key linking fields. Quality and completeness is always a concern when using routinely collected datasets and the large sample sizes they provide are always traded off with these limitations. Data quality was somewhat poorer in HES birth records than in ONS birth records and this is a commonly cited issue when working with HES data.[18,19] In studies using HES birth data in later years, the data quality improves with time. In 2009/10 the baby tail is far more complete, with only 18% and 14% of births with missing gestational age and birthweight, respectively.[18] Therefore, researchers who wish to analyse the linked data for the remaining years (2007-2014) should find a higher linkage rate with better quality linkage.

For peer review only - http://bmjopen.bmj.com/site/about/guidelines.xhtml

However, in previous work with mother's HES delivery records, the linkage rate plateaued at around 98% between 2010 and 2014 for singleton births, whereas the linkage rate in multiples began to decrease from 2010[7].

Compared to the full linked dataset, records of babies born in East England, North West England, London or the West Midlands were more likely to be unlinked. It is likely that these variations are due to differences between hospital trusts in the ways in which definitions or protocols are used or because errors occur during the transfer of data from one organisation to another or in the overall quality of Maternity HES. It is also possible that the regional reporting differences account for the higher proportion of white babies and births outside marriage in the unlinked dataset. Babies born in March were also more likely to have unlinked records and this was also the case with the mothers' HES delivery records[7]. HES uses financial years (1st April – 31st March) for report, so, differences in reporting standards prior to the financial year-end may account for this.

We found a small proportion of records that were potentially incorrect linkages, suggesting that linkage performed by 'Trusted Third Parties', such as NHS Digital, may not be 100% accurate. Therefore, it is essential that researchers understand the quality of linkage undertaken by a Trusted Third Party prior to performing any statistical analysis. Whilst the responsibility should fall to the Trusted Third Party to conduct quality assessment of their linkage methods and make them publicly available to researchers, this does not happen in practice.[20] The Digital Economy Act 2017 is designed to enable research for public benefit through the sharing of data, but the legislation is limited to the sharing of data that has been de-identified. This means reliance on the Trusted Third Party model in research is likely to increase over time. It is still unclear how this effects health and social care data, especially as they are not covered by the Digital Economy Act.[4] If researchers do not have access to personal identifiers, quality assurance of the linkage will become more challenging.

The findings in this paper will offer some insight into the quality of linkage between ONS birth and HES birth records and be of use to other researchers using the same linkage. As the quality and completeness of HES improved over time, it is likely that the quality of linkage will also have improved as a consequence, but the signs that the improvement in quality may not have been sustained is worrying.

Recommendations for future practice and research

The findings from this study have demonstrated a need for Trusted Third Parties to evaluate linkage methods and publish findings for researchers to use prior to beginning statistical analysis. In cases where this isn't possible, a number of studies have shown that datasets can be linked using de-identified datasets and produce generalisable cohorts, therefore, future work could explore ways to quality assure linkage without using personal identifiers. This will increase the likelihood of easier data access, potentially reducing the time required to complete the linkage and cleaning of routinely collected datasets. Finally, we did not assess whether the quality of linkage for multiples is comparable with live, singleton, NHS hospital births although quality assurance of the linkage of multiple births to mothers' delivery records suggested that it was better than had been initially assumed.[6].

For peer review only - http://bmjopen.bmj.com/site/about/guidelines.xhtml

Conclusions

By linking together administrative datasets, such as birth registration, birth notification and hospital admission data, it is possible to create more complete datasets about births which can then be linked to other administrative datasets to look at longer term outcomes.[6] If data are missing or there are coding errors and inconsistences, the resulting datasets can often be of poor quality. It is therefore essential that the quality of linkage is assessed prior to analysis. The work presented in this study provides a guide to steps taken to quality assure the linkage of births in England and may be of use to other researchers working with similar datasets. The findings will be particularly useful for researchers working with the same dataset but without access to personal identifiers.

For peer review only

Funding

This work was funded by the Medical Research Council (MRC): MR/M01228X/1. The funder had no input into the study design, data analysis, interpretation of results, or writing of the manuscript. VC and MQ had full access to all the data in the study and final responsibility for the decision to submit for publication.

Contributors

VC, MAQ and AJM were involved in the design of the study with input from Rod Gibson (Data management consultant) and Nirupa Dattani (Data analyst at City, University of London). RG and ND were involved in the cleaning of the ONS births dataset. VC performed the data manipulation and analysis. VC, AJM and MAQ were all involved in the interpretation of the data. VC was responsible for the initial draft of the manuscript. AJM and MAQ reviewed and contributed to drafts of the manuscript, and all authors have reviewed the final version.

Conflicts of interest

No conflicts of interest

Acknowledgements

We are grateful to the following organisations and individuals who were involved in this study: The MRC for funding this project (MR/M01228X/1); The Office for National Statistics (ONS) for providing access to data and hosting it within the Safe Research Setting (SRS); NHS Digital for linking and providing access to the data; Nirupa Dattani for providing her expertise on record linkage and data management; Rod Gibson, for his help cleaning the data; and Gillian Harper for her original work on the quality assurance of linkage of the mother's HES delivery records.

This work contains statistical data from ONS, which is Crown Copyright. The use of the ONS statistical data in this work does not imply the endorsement of the ONS in relation to the interpretation or analysis of the statistical data. This work uses research datasets, which may not exactly reproduce National Statistics aggregates.

Approvals

Ethics approval for this study was granted by the Health Research Authority Research Ethics Committee (South West – Frenchay; REC reference *Ethics* 15/SW/0294)

For peer review only - http://bmjopen.bmj.com/site/about/guidelines.xhtml

The TIGAR study used data linked as part of a previous study led by City, University of London.[6] For that study Permission to use patient-identifiable data without consent under Regulation 5 of the Health Service (Control of Patient Information) Regulations 2002 (‘section 251 support’) was initially granted by the Patient Information Advisory Group PIAG 2-10(g)/2005. Renewals and amendments and a second permission, CAG 9-08(b)2014, under Regulation 5 of the Health Service (Control of Patient Information) Regulations 2002 (or ‘same legislation’) were granted by the Secretary of State for Health and the Health Research Authority following advice from the CAG. A second permission CAG 9-08(b)2014 to use patient-identifiable data without consent under Regulation 5 of the Health Service (Control of Patient Information) Regulations 2002 and create a research database held at the ONS for analyses relating to inequalities in the outcome of pregnancy and to inform maternity service users about the outcome of midwifery, obstetric and neonatal care was granted by the Health Research Authority. For the TIGAR study, permission was granted by the Secretary of State for Health and the Health Research Authority following advice from the CAG (CAG reference 15/CAG/0196). Permission from the Health and Social Care Information Centre for the work described in this article was included in Data Sharing Agreement NIC-273840-N0N0 N. Permission for the subsequent analyses in the TIGAR study were included in Data Sharing Agreement.

Data Sharing Statement

The authors do not have permission to supply data or identifiable information to third parties, including other researchers, but the team at City, University of London has permission under Regulation 5 of the Health Service (Control of Patient Information) Regulations 2002 to analyse patient-identifiable data for England and Wales without consent and create a research database that could be accessed by other researchers using the SRS at the ONS. The TIGAR team has permission under Regulation 5 of the Health Service (Control of Patient Information) Regulations 2002 to analyse these. Anyone wishing to access the linked datasets for research purposes should apply via the CAG to the Health Research Authority to access patient-identifiable data without consent and then to the ONS and NHS Digital. In the first instance, enquiries about access to the data should be addressed to Alison Macfarlane.

For peer review only - http://bmjopen.bmj.com/site/about/guidelines.xhtml

References

1. Jorm, L. Routinely collected data as a strategic resource for research: priorities for methods and workforce. *Public Heal. Res. Pract.* **25**, 1–5 (2015).
2. Künn, S. The challenges of linking survey and administrative data. *IZA World Labor* (2015). doi:10.15185/izawol.214
3. House of Commons. *Digital Economy Act. The National Archives* (2017).
4. Bell, J. *et al.* Lawful disclosure of administrative data for research purposes in the UK. *J. Data Prot. Priv.* **2**, 264–277 (2019).
5. Dattani, N. & Macfarlane, A. Linkage of Maternity Hospital Episode Statistics data to birth registration and notification records for births in England 2005-2014: methods. A population-based birth cohort study. *BMJ Open* **8**, 1–7 (2018).
6. Macfarlane, A. *et al.* Births and their outcomes by time, day and year: a retrospective birth cohort data linkage study. *Heal. Serv. Deliv. Res.* **7**, (2019).
7. Harper, G. Linkage of Maternity Hospital Episode Statistics data to birth registration and notification records for births in England 2005-2014: Quality assurance of linkage of routine data for singleton and multiple births. *BMJ Open* **8**, (2018).
8. ONS. *Birth statistics: Review of the Registrar General on births and patterns of family building in England and Wales, 2006*. (2007).
9. StataCorp. Stata Statistical Software: Release 14. (2015).
10. Hilder, L., Moser, K., Dattani, N. & Macfarlane, A. Pilot linkage of NHS Numbers for Babies data with birth registrations. *Heal. Stat. Q.* 25–33 (2007).
11. Herbert, A., Wijlaars, L., Zylbersztejn, A., Cromwell, D. & Hardelid, P. Data Resource Profile: Hospital Episode Statistics Admitted Patient Care (HES APC). *Int. J. Epidemiol.* **46**, 1093-1093i (2017).
12. HSCIC. *Methodology for creation of the HES Patient ID (HESID)*. (2014).
13. NHS DIGITAL. HES Data Dictionary - Admitted Patient. *HSCIC* (2018). Available at: file:///Users/victoriacoathup/Downloads/DD-APC-V11.pdf. (Accessed: 6th June 2018)

For peer review only - http://bmjopen.bmj.com/site/about/guidelines.xhtml

14. Herbert, A., Wijlaars, L., Zylbersztejn, A., Cromwell, D. & Hardelid, P. Data Resource Profile: Hospital Episode Statistics Admitted Patient Care (HES APC). *Int. J. Epidemiol.* **46**, 1093-1093i (2017).

15. Hagger-Johnson, G. *et al.* Identifying possible false matches in anonymized hospital administrative data without patient identifiers. *Health Serv. Res.* **50**, 1162–1178 (2015).

16. Paixão, E. S. *et al.* Evaluation of record linkage of two large administrative databases in a middle income country: Stillbirths and notifications of dengue during pregnancy in Brazil. *BMC Med. Inform. Decis. Mak.* **17**, 1–9 (2017).

17. Harron, K., Gilbert, R., Cromwell, D. & Van Der Meulen, J. Linking data for mothers and babies in de-identified electronic health data. *PLoS One* **11**, 1–18 (2016).

18. Murray, J. *et al.* Quality of routine hospital birth records and the feasibility of their use for creating birth cohorts. *J. Public Heal. (United Kingdom)* **35**, 298–307 (2013).

19. Ghosh, R. E. *et al.* Routinely collected English birth data sets: Comparisons and recommendations for reproductive epidemiology. *Arch. Dis. Child. Fetal Neonatal Ed.* **101**, F451–F457 (2016).

20. Harper, G. Linkage of Maternity Hospital Episode Statistics data to birth registration and notification records for births in England 2005-2014: Quality assurance of linkage of routine data for singleton and multiple births. *BMJ Open* **8**, (2018).

For peer review only

Figure Legends

Figure 1. Flowchart of study population

Figure S1. Overview of data sources

For peer review only

For peer review only - http://bmjopen.bmj.com/site/about/guidelines.xhtml

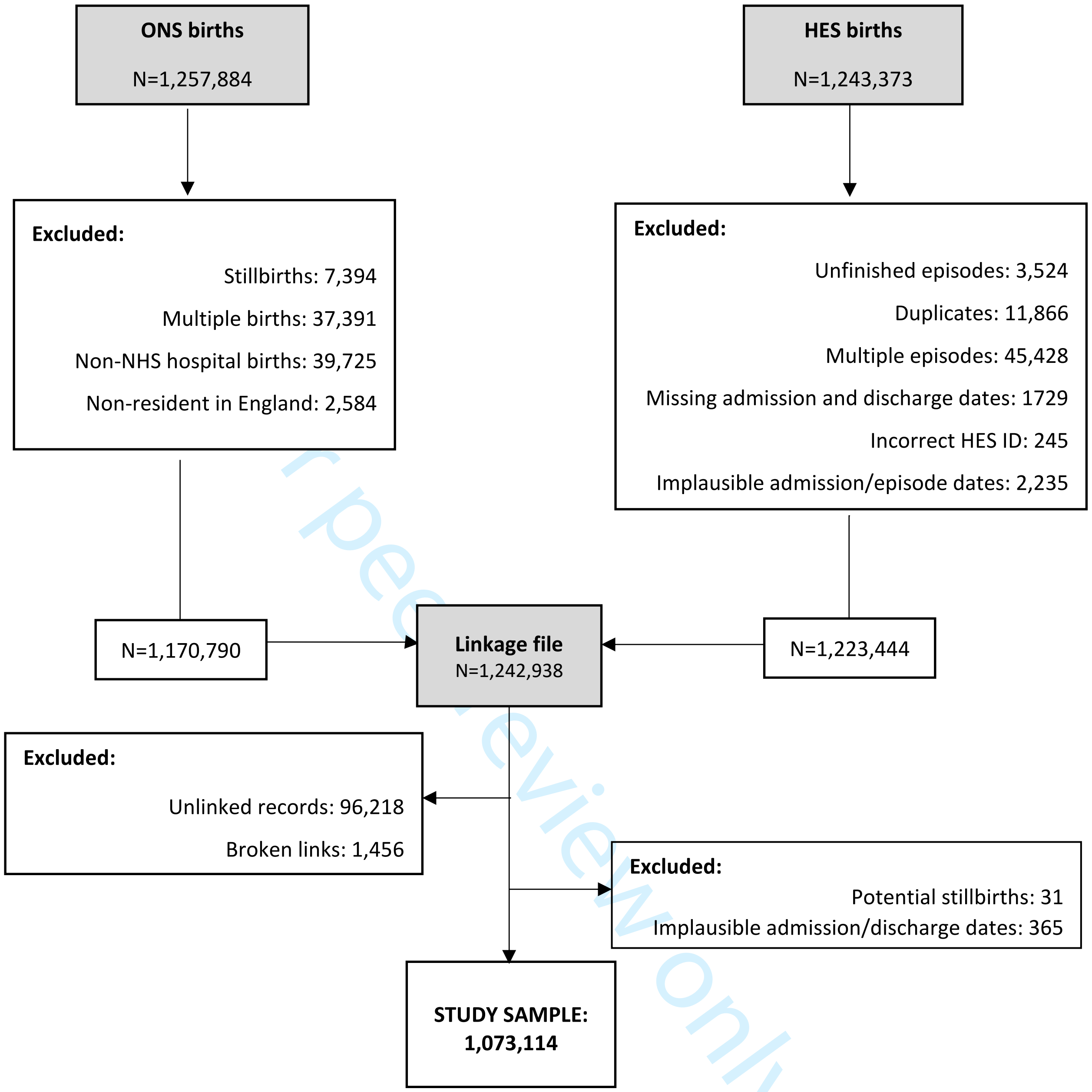

For peer review only - http://bmjopen.bmj.com/site/about/guidelines.xhtml

Supplementary information

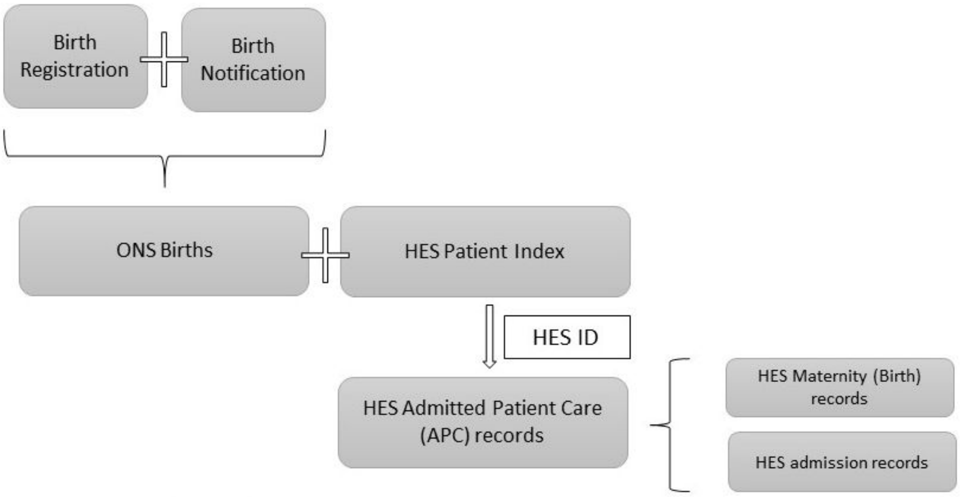

HES = Hospital Episode Statistics
ONS = Office for National Statistics
NHS Digital performed linkage between ONS Births and HES Patient Index

Figure S1. Overview of data sources

List of variables within the 'baby tail':

1. Anaesthetic given during labour or delivery (delpren)
2. Anaesthetic given post-labour or delivery (delposn)
3. Antenatal days of stay (antedur) (derived from other HES fields)
4. Baby's age in days (neodur) (derived from other HES fields)
5. Birth order (birorder)
6. Birth weight (birweit)
7. Delivery place change reason (delchang)
8. Delivery method (delmeth)
9. Delivery place (actual) (delplace)
10. Delivery place (intended) (delinten)
11. First antenatal assessment date (anasdate)
12. Gestation in weeks at first antenatal assessment (anagest)
13. Length of gestation (gestat)
14. Birth status (birstat)
15. Labour/delivery onset method (delonset)
16. Neonatel level of care (neocare)
17. Number of babies (numbaby)
18. Resuscitation method (biresus)
19. Status of person conducting delivery (delstat)

For peer review only - http://bmjopen.bmj.com/site/about/guidelines.xhtml

Definitions of Episode, Spell and Continuous Inpatient Stay (CIP)

HES inpatient admissions are structured as ‘episodes’ of care, with an episode defined as a period of care under one consultant or midwife. Multiple episodes are referred to as a ‘spell’ and represent an uninterrupted period of care within one hospital. A new spell is generated when the patient is transferred to another hospital to continue care. A continuous inpatient stay (CIP) may consist of one episode or multiple episodes and spells, and ends when the patient is discharged from an NHS hospital. Hospital episodes are primarily collected for financial reimbursement, and therefore, the datasets are divided into financial years, beginning 1st April and ending 31st March. Episodes are labelled as ‘finished’ once the patient is discharged from hospital. However, if an episode begins in one financial year and ends during the next, two episodes will be generated – one in the financial year the episode begins and one in the financial year that the episode ends – therefore, the first episode will remain as ‘unfinished’.

- Unfinished episodes identified using episode type (EPITYPE = 1)

- Transfers identified using admission method, admission source and discharge destination (ADMIMETH = 81; ADMISORC = 49/50/51/52/53/87; or DISDEST = 49/50/51/52/53/84).

For peer review only

Table S1. NHS Digital algorithm used to link ONS Births with HES birth records

Match rank	Variables				Total		2005		2006	
	NHS number	Date of birth	Sex	Postcode	% Linked	% Broken	% Linked	% Broken	% Linked	% Broken
1	E	E	E	E	*80.43*	*28.23*	*75.68*	*24.61*	*85.01*	*34.45*
2	E	E	E	-	*19.20*	*9.41*	*24.02*	*8.76*	*14.52*	*10.42*
3	E	P°	E	E	*0.02*	*1.31*	*0.01*	*1.35*	*0.02*	*1.59*
4	E	P°	E	-	*0.01*	*1.03*	*0.01*	*1.12*	*0.01*	*0.71*
5	E	-	-	E	*0.02*	*2.61*	*0.02*	*1.57*	*0.03*	*4.59*
6	M	E*	E	E^	*0.31*	*53.50*	*0.22*	*58.76*	*0.40*	*44.52*
7	M	E*	E	E	*0.01*	*1.10*	*0.01*	*1.35*	*0.01*	*0.68*
8	E	-	-	-	*0.02*	*2.82*	*0.03*	*2.47*	*0.01*	*3.13*
Number of NHS births					1,073,114	1,456	525,826	890	100	566

E=Exact match, P=Partial match, M=No match, due to missing data
°Two components of data match or DOB matches, when two components are swapped
**Date of birth not 1st January*
^Postcode not on 'ignore' list

For peer review only

Table S2. Sources of variables for quality assurance

Variable	Source	Rule
Baby's date of birth	BR & BN	Use BR unless BR = missing, then use BN
Mother's date of birth	BR & BN	Use BR unless BR = missing, then use BN
Birth weight	BR & BN	Use BR unless BR = missing, then use BN
Gestational age	BR & BN	use BN as BR reported for still births only
Sex	BR & BN	Use BR unless BR = missing, then use BN
Hospital of birth	BR & BN	Use BR unless BR = missing, then use BN

BR = Birth Registration
BN = Birth Notification (NN4B)

For peer review only

For peer review only - http://bmjopen.bmj.com/site/about/guidelines.xhtml

Table S3. Data preparation rules for quality assurance variables

Variable	Variable name: HES	Variable name: BR/BN	Rule	Additional checks
Baby's date of birth	DOBBABY	DOB/DOBZ	Set '01Jan1801' to missing. Make sure all dates are in format 'DDMMMYYYY' e.g. '01Jan2005'.	Check dates are not <01/01/05 or > 31/12/06
Mother's date of birth	MOTDOB	DOBM/DOBMZ	Set '01Jan1801' to missing. Make sure all dates are in format 'DDMMMYYYY' e.g. '01Jan2005'	
Postcode	HOMEADD	PCDRBZ (BN)	Transform var into format of 'XXX XXX', by removing any additional punctuation and transforming any lower case letters to upper case	
Birth weight	BIRWEIT	BTHWGTZ (BN)	Set '99', '9999', <400 and ≥7000 to missing	Check all hospitals report birth weight in g. If it looks like kg, then convert to equivalent in g.
Gestational age	GESTAT	GESTZ (BN)	Set '99' to missing	Check all hospital report gestational age in weeks, if not, convert from days or months to weeks
Sex	SEXBABY	SEXZ (BN)	Transform M to 1 and F to 2 if string variable. Set '0', '3' or '9' to missing.	
Hospital of birth	PROCODE	Location Code (derived from CESTRESS/ORGCODEZ)	Remove punctuation. Transform any lower case letters to upper case	

HES = Hospital Episode Statistics
BR = Birth Registration, BN= Birth Notification (NN4B)

For peer review only

Table S4. Percentages of data missing from each dataset for key variables used to evaluate linkage of data about live, singleton births in NHS hospitals to women living in England

	ONS Births			HES Births		
**Variable**	**Total**	**2005**	**2006**	**Total**	**2005**	**2006**
NHS number	0.1	0.1	0.1	6.9	6.5	7.4
Baby’s date of birth	0.0	0.0	0.0	0.0	0.0	0.0
Mother's date of birth	0.0	0.0	0.0	42.7	42.9	42.4
Mother's postcode	0.0	0.0	0.0	60.4	76.2	45.0
Hospital location code	0.0	0.0	0.0	0.0	0.0	0.0
Birthweight	0.1	0.1	0.1	44.4	43.8	45.1
Gestational age	0.8	0.8	0.8	49.1	45.7	52.6
Sex	0.0	0.0	0.0	0.0	0.0	0.0

For peer review only

For peer review only - http://bmjopen.bmj.com/site/about/guidelines.xhtml

Steps taken to deal with multiple HES birth records with the same HESID:

1. EPISTAT is 1 = unfinished episode: exclude all (for details of HES variables, see *NHS Digital Data Dictionary)*
2. HESID and EPIKEY exact match = duplicate record: keep any and exclude rest
3. HESID, DOB, Sex, hospital code, EPIORDER, ADMIDATE, DISDATE, EPISTART, EPIEND, DIAG_01 – DIAG_20, OPCS_01-OPCS_24, OPDATE_01-OPDATE_24 exact match and MDOB and POSTCODE either exact match or missing match = duplicate record: keep any and exclude rest
4. Two or more key variables (date of birth, Hospital code, mother's date of birth, postcode, birthweight) differ completely = unclear which record contains correct information: exclude all
5. Remainder are considered multiple episodes part of same spell = create new record using all available information from multiple episodes: keep one record and save other episodes in separate file
6. Dataset should now contain one HES birth record per baby

For peer review only

For peer review only - http://bmjopen.bmj.com/site/about/guidelines.xhtml

For peer review only

For peer review only - http://bmjopen.bmj.com/site/about/guidelines.xhtml

The RECORD statement – checklist of items, extended from the STROBE statement, that should be reported in observational studies using routinely collected health data.

	Item No.	STROBE items	Location in manuscript where items are reported	RECORD items	Location in manuscript where items are reported
**Title and abstract**					
	1	(a) Indicate the study's design with a commonly used term in the title or the abstract (b) Provide in the abstract an informative and balanced summary of what was done and what was found	Study design is included in the title and abstract	RECORD 1.1: The type of data used should be specified in the title or abstract. When possible, the name of the databases used should be included.<br><br>RECORD 1.2: If applicable, the geographic region and timeframe within which the study took place should be reported in the title or abstract.<br><br>RECORD 1.3: If linkage between databases was conducted for the study, this should be clearly stated in the title or abstract.	Type of data included in the title. Databases included in abstract<br><br>Region in title, region and timeframe mentioned in abstract<br><br>Stated in both the title and abstract
**Introduction**					
Background rationale	2	Explain the scientific background and rationale for the investigation being reported	Introduction		
Objectives	3	State specific objectives, including any prespecified hypotheses	At the end of the introduction		
**Methods**					
Study Design	4	Present key elements of study design early in the paper	Design and study population mentioned on page 3 at beginning of methods		

For peer review only

Setting	5	Describe the setting, locations, and relevant dates, including periods of recruitment, exposure, follow-up, and data collection	Described in study population section in methods		
Participants	6	*(a) Cohort study* - Give the eligibility criteria, and the sources and methods of selection of participants. Describe methods of follow-up<br>*Case-control study* - Give the eligibility criteria, and the sources and methods of case ascertainment and control selection. Give the rationale for the choice of cases and controls<br>*Cross-sectional study* - Give the eligibility criteria, and the sources and methods of selection of participants<br><br>*(b) Cohort study* - For matched studies, give matching criteria and number of exposed and unexposed<br>*Case-control study* - For matched studies, give matching criteria and the number of controls per case	Study population section within methods<br><br>N/A	RECORD 6.1: The methods of study population selection (such as codes or algorithms used to identify subjects) should be listed in detail. If this is not possible, an explanation should be provided.<br><br>RECORD 6.2: Any validation studies of the codes or algorithms used to select the population should be referenced. If validation was conducted for this study and not published elsewhere, detailed methods and results should be provided.<br><br>RECORD 6.3: If the study involved linkage of databases, consider use of a flow diagram or other graphical display to demonstrate the data linkage process, including the number of individuals with linked data at each stage.	Methods and supplementary information<br><br>N/A<br><br>Figure 1
Variables	7	Clearly define all outcomes, exposures, predictors, potential confounders, and effect modifiers. Give diagnostic criteria, if applicable.	N/A	RECORD 7.1: A complete list of codes and algorithms used to classify exposures, outcomes, confounders, and effect modifiers should be provided. If these cannot be reported, an explanation should be provided.	Supplementary information and appropriate references to sources of codes
Data sources/ measurement	8	For each variable of interest, give sources of data and details of methods of assessment (measurement).	Methods and supplementary information – but only key variables		

For peer review only

		Describe comparability of assessment methods if there is more than one group	with more than one source		
Bias	9	Describe any efforts to address potential sources of bias	Methods and supplementary information		
Study size	10	Explain how the study size was arrived at	Figure 1		
Quantitative variables	11	Explain how quantitative variables were handled in the analyses. If applicable, describe which groupings were chosen, and why	Outcomes, exposures and statistical analysis sections of methods		
Statistical methods	12	(a) Describe all statistical methods, including those used to control for confounding<br>(b) Describe any methods used to examine subgroups and interactions<br>(c) Explain how missing data were addressed<br>(d) *Cohort study* - If applicable, explain how loss to follow-up was addressed<br>*Case-control study* - If applicable, explain how matching of cases and controls was addressed<br>*Cross-sectional study* - If applicable, describe analytical methods taking account of sampling strategy<br>(e) Describe any sensitivity analyses	Assessment of linkage bias section in methods		
Data access and cleaning methods		..		RECORD 12.1: Authors should describe the extent to which the investigators had access to the database	Author contributions and Supplementary information

For peer review only

				population used to create the study population.<br><br>RECORD 12.2: Authors should provide information on the data cleaning methods used in the study.	
Linkage		..		RECORD 12.3: State whether the study included person-level, institutional-level, or other data linkage across two or more databases. The methods of linkage and methods of linkage quality evaluation should be provided.	Methods
**Results**					
Participants	13	(a) Report the numbers of individuals at each stage of the study (*e.g.*, numbers potentially eligible, examined for eligibility, confirmed eligible, included in the study, completing follow-up, and analysed)<br>(b) Give reasons for non-participation at each stage.<br>(c) Consider use of a flow diagram	Figure 1	RECORD 13.1: Describe in detail the selection of the persons included in the study (*i.e.,* study population selection) including filtering based on data quality, data availability and linkage. The selection of included persons can be described in the text and/or by means of the study flow diagram.	Figure 1
Descriptive data	14	(a) Give characteristics of study participants (*e.g.*, demographic, clinical, social) and information on exposures and potential confounders<br>(b) Indicate the number of participants with missing data for each variable of interest<br>(c) *Cohort study* - summarise follow-up time (*e.g.*, average and total amount)	Table 4 in results section		

For peer review only

Outcome data	15	*Cohort study* - Report numbers of outcome events or summary measures over time<br>*Case-control study* - Report numbers in each exposure category, or summary measures of exposure<br>*Cross-sectional study* - Report numbers of outcome events or summary measures	Table 4 in results section		
Main results	16	(a) Give unadjusted estimates and, if applicable, confounder-adjusted estimates and their precision (e.g., 95% confidence interval). Make clear which confounders were adjusted for and why they were included<br>(b) Report category boundaries when continuous variables were categorized<br>(c) If relevant, consider translating estimates of relative risk into absolute risk for a meaningful time period	N/A		
Other analyses	17	Report other analyses done—e.g., analyses of subgroups and interactions, and sensitivity analyses	N/A		
**Discussion**					
Key results	18	Summarise key results with reference to study objectives	First paragraph of discussion		
Limitations	19	Discuss limitations of the study, taking into account sources of potential bias or imprecision. Discuss both direction and magnitude of any potential bias	Strengths and limitations section of discussion	RECORD 19.1: Discuss the implications of using data that were not created or collected to answer the specific research question(s). Include discussion of misclassification bias, unmeasured confounding, missing data, and changing eligibility over	Strengths and limitations section of discussion

For peer review only

				time, as they pertain to the study being reported.	
Interpretation	20	Give a cautious overall interpretation of results considering objectives, limitations, multiplicity of analyses, results from similar studies, and other relevant evidence	Interpretation of findings section within the discussion		
Generalisability	21	Discuss the generalisability (external validity) of the study results	Interpretation of findings section within the discussion		
**Other Information**					
Funding	22	Give the source of funding and the role of the funders for the present study and, if applicable, for the original study on which the present article is based	Funding included at end of manuscript		
Accessibility of protocol, raw data, and programming code		..		RECORD 22.1: Authors should provide information on how to access any supplemental information such as the study protocol, raw data, or programming code.	Supplementary information

*Reference: Benchimol EI, Smeeth L, Guttmann A, Harron K, Moher D, Petersen I, Sørensen HT, von Elm E, Langan SM, the RECORD Working Committee. The REporting of studies Conducted using Observational Routinely-collected health Data (RECORD) Statement. *PLoS Medicine* 2015; in press.

*Checklist is protected under Creative Commons Attribution (CC BY) license.

For peer review only - http://bmjopen.bmj.com/site/about/guidelines.xhtml
